# Focus prosody in Qassimi Arabic: acoustic analysis and computational modeling

**DOI:** 10.3389/fnhum.2026.1928391

**Published:** 2026-10-07

**Authors:** Muhammad Swaileh A. Alzaidi, Yi Xu, Buthainah M. Al Thowaini, Mohammad A. S. Bani Younes

**Affiliations:** 1 Department of English, College of Language Sciences, King Saud University, Riyadh, Saudi Arabia; 2 Department of Human Communication Sciences, University College London, London, United Kingdom; 3 Department of English Language and Literature, Faculty of Foreign Languages, Al al-Bayt University, Mafraq, Jordan

**Keywords:** acoustic phonetics, computational modeling, focus, intonation, PENTA, PENTAtrainer, post-focus compression, Qassimi Arabic

## Abstract

This study investigates focus intonation in Qassimi Arabic (QA) with two aims: to examine focus-related prosodic changes in the focused word and surrounding regions, and to identify the functional factors shaping intonation and their relative contributions. The analysis considers maximum, minimum, and mean *F*_0_, excursion size, duration, and intensity. The data comprise 1,800 utterances produced by ten native speakers in neutral-focus, information-focus, and contrastive-focus contexts. The acoustic results show that focus is realized primarily through enhancement of the focused word, especially through *F*_0_-register raising and, in some positions, increased pitch excursion. These effects are strongest in sentence-initial and sentence-penultimate positions, while sentence-final focus shows more limited register raising. The pre-focus region remains largely stable. In the broad post-focus region, information and contrastive focus are followed by significant intensity attenuation but no consistent changes in *F*_0_, excursion size, or duration. However, localized analysis of the post-focus word following sentence-penultimate focus reveals reduced excursion size after contrastive focus, but not after information focus. The results therefore indicate cue- and position-specific post-focus modification rather than general post-focus *F*_0_ compression. No consistent acoustic distinction emerges between information and contrastive focus across measures and positions. PENTAtrainer modeling reproduced major properties of the observed *F*_0_ contours, including on-focus prominence, declination, phrase-final lowering, and condition-dependent post-focus modification. Function-removal analyses showed that prosodic-word structure and discourse focus contributed most strongly, whereas syllable weight contributed least. Overall, the findings support a multi-functional account of Qassimi Arabic focus prosody and the applicability of the PENTA framework to Arabic intonation.

## 1 Introduction

### 1.1 Prosodic focus

Focus is an important aspect of prosody in speech that conveys additional information beyond the lexical meanings of a sentence (Halliday, 1967; Chafe, 1976; Lambrecht, 1994). As shown in (1.1), the question *Who watched the game?* is a request for the missing information to be provided in an answer, and the reply *[Peter] watched the game* not only provides the information, *Peter*, but also highlights the information by putting prosodic focus on *Peter*. This is known as information focus. In (2.2), the reply *[Peter] watched the game* is a correction to the question *Who watched the game? John?*. And this is also done by not only providing the correct information, but also putting a prosodic focus on *Peter*. This is known as contrastive focus.

(1) 1. Who watched the game?
      2. [Peter] watched the game.

(2) 1. Who watched the game? John?
      2. [Peter] watched the game.

Additionally, focus varies in scope. When the entire proposition introduces new information, as in (3.1), it constitutes neutral or broad focus (NF) (Ladd, 2008). By contrast, narrow focus highlights a specific constituent, as in information focus (1.1) and contrastive focus (2.2). This distinction is important for understanding cross-linguistic variation in the prosodic realization of focus (Alzaidi et al., 2023, 2025).

(3) 1. What happened?
      2. [Peter watched the game.]

The acoustics of prosodic focus has been extensively researched for many languages, and an important finding is that focus is marked not just locally on the focused item itself, but globally on pre-focus, on-focus as well as post-focus words (Cooper et al., 1985; Xu, 2011a; Xu and Xu, 2005). Relative to neutral focus, pre-focused words are either raised (Ipek, 2011; Alzaidi, 2022), or lowered (Chahal, 2001; Yeou et al., 2007) in pitch, and sometimes in intensity (Ardali and Xu, 2012; Alzamil and Hellmuth, 2022; Alzaidi et al., 2025) and duration (Jun and Lee, 1998; Alzaidi et al., 2023). On-focus words show increased pitch, intensity and syllable duration (Cooper et al., 1985; Eady and Cooper, 1986; Xu, 1999; Breen et al., 2010; O'Rourke, 2012). In post-focus words, both pitch and intensity are lowered (Cooper et al., 1985; Xu, 1999; Chen et al., 2009; Ipek, 2011; Xu et al., 2012), but no consistent changes in syllable duration (Cooper et al., 1985). Post-focus compression (PFC), which is one of the three types of modulations, is the most consistent in languages with clear focus marking (Eady and Cooper, 1986; Xu, 2011a). On the other hand, PFC is found to be non-universal, because it is absent in many languages (Zerbian et al., 2010; Xu et al., 2012; Downing and Rialland, 2016). Interestingly, the cross-language distribution of PFC seems to follow language family lines. It is present in Indo-European, Altaic, Uralic, Dravidian languages, but absent in most other language families, with the exception of mixed distributions in two language families, namely, Afroasiatic (Hebrew, Arabic) and Sino-Tibetan (Xu et al., 2010; Xu, 2019). This distribution pattern has led to a proposal of the single origin of PFC hypothesis (Xu et al., 2010), which posits that PFC originated from the Fertile Crescent 11,000 years ago and spread with language expansion with the proliferation of wheat and barley and cattle-based agriculture (Diamond and Bellwood, 2003). This hypothesis has been under scrutiny, and one of the obvious methods is to test the validity of the language-family-based distribution of PFC. Therefore, this study aims to examine whether Qassimi Arabic (QA) shows post-focus compression. In doing so, it also contributes to the broader work on how PFC is distributed across Arabic dialects (Alzaidi et al., 2023, 2025, in press).

This view of focus as a separate prosodic function differs from the Autosegmental-Metrical approach (AM) to intonation (Pierrehumbert, 1980; Gussenhoven, 1984; Ladd, 2008). Within AM theory, focus is not treated as a phonological category in its own right. Rather, it is projected by tonal units that are phonological, including pitch accents, phrase accents, and boundary tones (Pierrehumbert, 1980; Pierrehumbert and Hirschberg, 1990; Ladd, 2008). AM theory views speech prosody as composed of a single linear sequence of phonological intonational units. Although these phonological units are subject to pitch range expansion and compression, those modifications are viewed as phonetically gradient rather phonological. The consequence of this conceptualization is that, being phonetic, the focus-related pitch range changes are not considered as contrastive beyond the theory-endorsed phonological tonal units.

A further issue with the AM account is its treatment of nuclear accent. AM theory assumes that every intonational phrase must contain a nuclear accent. For this reason, sentences with no clear focus position are often treated as having broad-focus. In such cases, the nuclear accent is assumed to fall on the final word by default, or on its stressed syllable (Ladd, 2008). The problem with this argument is that such obligatory default sentence-final nuclear accent would make it phonologically indistinct from a sentence final focus that is functionally motivated. This would undermine the justification for experimentally comparing the prosody of sentence final focus to that of a control condition where no position-specific focus is present.

Additionally, AM theory assumes that the function of focus is to mark new information, which has been contradicted by evidence from neural studies (Wang et al., 2017). Independently controlled ERP studies show that focused words elicit a larger P2 and greater positivity than unfocused words, whereas words carrying new information elicit a larger N400 and a smaller late positive complex than given words, indicating that focus and newness are subserved by dissociable cognitive processes: the former reflecting attention allocation and immediate integration of the highlighted constituent, the latter reflecting difficulty of information integration and memory retrieval (Chen et al., 2014). Convergent evidence comes from fMRI, which shows that prosodic focus marking recruits a general-purpose attention network during spoken language comprehension (Kristensen et al., 2013), and from further behavioral and neural evidence linking focus, as distinct from mere newness, specifically to attentional engagement during discourse processing (Chen et al., 2012).

### 1.2 The PENTA framework

An alternative to the AM theory is the Parallel Encoding and Target Approximation (PENTA) model (Xu, 2004, 2005). PENTA assumes that surface prosodic forms are the outcome of parallel encoding (PE) of multiple layers of communicative functions realized through the articulatory process of target approximation (TA). In this framework, multiple communicative functions can be encoded simultaneously by jointly determining the target approximation parameters of a linear sequence of articulatory intervals, as illustrated in Figure 1.

**Figure 1:**
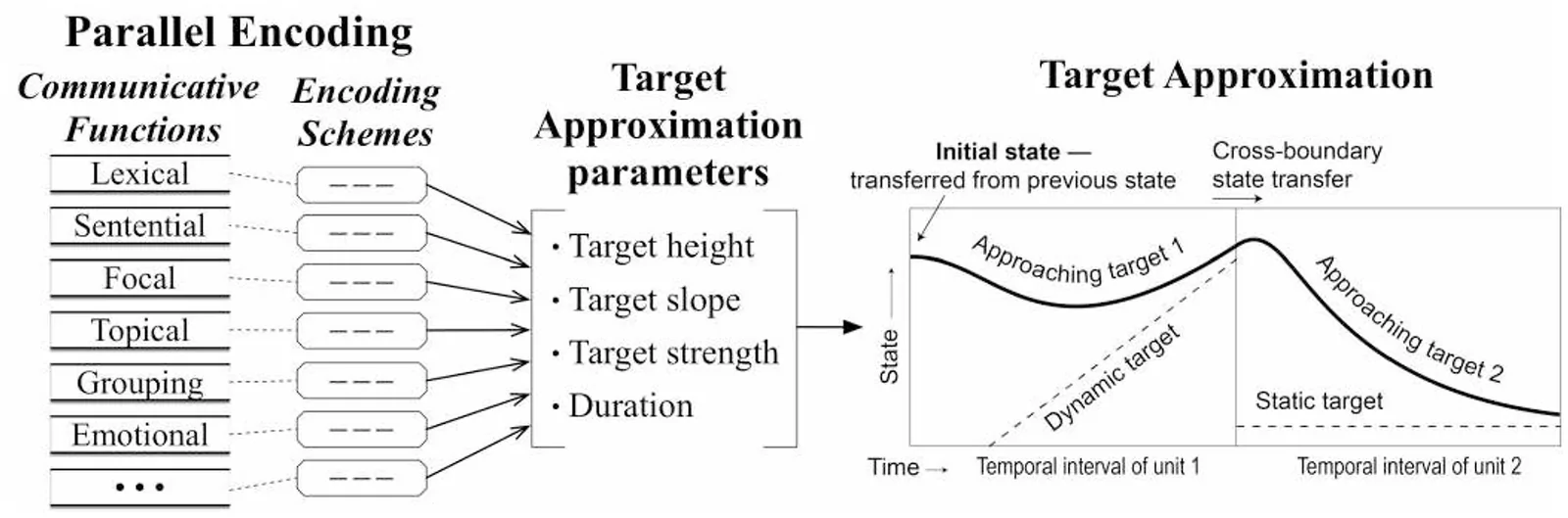
A schematic view of the PENTA framework. The model shows how different communicative functions are encoded in parallel. These functions guide target approximation and help generate the continuous *F*_0_ contour. Adapted from Xu and Wang (2001), Xu (2005), and Xu and Liu (2012).

As shown in Figure 1, multiple communicative functions (block 1 from left) are encoded in speech in parallel without a hierarchical order. They each have their respective encoding schemes (block 2) that contribute jointly to the target approximation parameters–target height, target slope, target strength, and interval duration (block 3). These multi-functional parameters then control the articulatory process of sequential target approximation, whereby the vocal system continuously approximates successive pitch targets, each associated with a specific time interval, to generate a sequence of smoothly connected *F*_0_ contours. The framework therefore postulates explicitly how multiple communicative functions can be simultaneously encoded in speech production.

In practice, the time intervals used under PENTA often correspond to the syllable, which is in accordance with the synchronization model of the syllable (Xu and Liu, 2006; Xu, 2024). This model assumes that every syllable in a language is associated with an underlying pitch target, whose articulation starts in synchrony with the onset of both the initial consonant and the vowel, and terminates at the end of the syllable. Following this approach, intonation is formed by the articulatory approximation of syllable-bound pitch targets whose specifications come from combined contributions of multiple layers of communicative functions. This conceptualization has been quantified into PENTAtrainer, a semiautomatic intonation modeling tool to be detailed later in Section 3.1. The modeling tool enables the examination of hypothetical communicative functions by including or excluding them in the model optimization process to assess how well they contribute to the generation of intonational contours that can be compared to *F*_0_ contours produced by native speakers (Xu and Prom-On, 2014; Alzaidi et al., 2023). PENTAtrainer is particularly suitable for Qassimi Arabic because the dialect has a quantity-sensitive stress system and systematic interactions among focus, syllable weight, stress position, and prosodic-word structure, all of which can be represented as parallel functional layers and evaluated through function-removal modeling (discussed later in Section 1.4).

The function-first principle of PENTA also allows it to justify the need to compare any functionally motivated position-specific focus, including sentence-final focus, to a control condition where there is no position-specific focus in the sentence. Such control condition is usually referred to as neutral focus, rather than as broad focus which implies, a priori, lack of contrastive distinction between default and functionally triggered sentence-final focus.

This treatment of neutral focus as a baseline, rather than as a genuine “no focus” condition, also underlies how the pre-focus, on-focus, and post-focus regions are defined later in this study, both in the acoustic analyses (Section 2.1.4) and in the PENTAtrainer functional annotation (Section 3.1); the rationale is elaborated in those sections.

A distinction that has often been considered important is that between information focus (IF) and contrastive focus (CF) (Gussenhoven, 2002; Féry and Ishihara, 2016), as mentioned near the beginning of the introduction. Empirical research, however, has shown mixed evidence on the contractiveness of this distinction. Many studies were not able to find significant differences between the two focus types (Sityaev and House, 2003; Hanssen et al., 2008; Hwang, 2012). Some other studies reported slight differences between them (He et al., 2011; Sahkai et al., 2013; Alzaidi et al., 2025). For Arabic, Alzaidi et al. (in press), Alzaidi et al. (2023) and Alzaidi et al. (2025) did find significant difference between the two focus types, although it was attributed to the elicitation of incredulity associated with contrastive focus in the study.

### 1.3 The case of Arabic

Arabic, as an Afro-Asiatic language, holds an important position in the research of focus. It is one of the major languages on the path of the farming-language expansion from the Fertile Crescent (Diamond and Bellwood, 2003). Previous research on focus prosody in Arabic dialects has reported considerable variation across dialects. For example, studies on Lebanese, Egyptian, Emirati, Hijazi, and other Arabic varieties have shown that focus is realized with PFC (Chahal and Hellmuth, 2014; Alzaidi et al., 2019, 2023, in press; Alzamil and Hellmuth, 2022). However, Makkan Arabic is found to lack PFC (Alzaidi, 2022). Studies also report different findings on whether information focus and contrastive focus are prosodically distinct. For Hijazi Arabic, Alzaidi et al. (2019) showed that contrastive focus was more prominent than information focus in the on-focus region. It had a larger pitch excursion, higher *F*_0_, longer duration, and greater intensity. Subsequent studies, however, have found considerable variations across Arabic dialects. In Emirati Arabic, the two focus types differed mainly in pitch excursion across sentence positions. They also differed in mean intensity in sentence-initial and sentence-penultimate positions (Alzaidi et al., 2023). Iraqi Arabic shows a similar pattern. The differences were limited, but contrastive focus had slightly stronger intensity and clearer effects on the surrounding *F*_0_ contour than information focus (Alzaidi et al., 2025). By contrast, Makkan Arabic showed no systematic prosodic difference between the two focus types (Alzaidi, 2022). Together, these findings suggest that prosodic marking of focus type varies across Arabic dialects. However, some of the reported cross-dialectal differences may also reflect methodological variation across studies, including differences in elicitation procedures, focus definitions, sentence materials, acoustic measures, speaker samples, and statistical analyses. They also point to the need for further work. The present study addresses this issue by examining focus realization in Qassimi Arabic.

The substantial cross-dialectal variability reported in Arabic focus research also poses a challenge for computational accounts of intonation. If dialects differ in the acoustic realization of focus and in the functional role of individual prosodic cues, computational models must be capable of capturing not only general prosodic structure but also dialect-specific patterns of focus encoding. Such variation highlights the need for quantitative approaches that can evaluate the relative contribution of multiple acoustic parameters and assess how effectively focus-related distinctions can be represented within a unified modeling framework.

Although Arabic computational linguistics and speech technologies have expanded considerably in recent decades, computational modeling of Arabic intonation and communicative prosody remains comparatively limited, particularly for dialectal Arabic varieties (Habash, 2010; Ditters, 2013; Khalifa et al., 2016). Much Arabic computational research has focused on applications such as speech recognition, machine translation, morphological analysis, sentiment analysis, and dialect identification (Habash and Sadat, 2006; Elfardy and Diab, 2013; Pasha et al., 2014; Alzaidi et al., 2024). Comparatively less attention has been devoted to prosody-sensitive modeling and the functional representation of intonation. One notable exception is the work of Brown and Hellmuth (2022), who demonstrated that both segmental and prosodic features contribute to computational Arabic dialect classification. Using the Y-ACCDIST framework, they modeled dialectal variation through sentence-level *F*_0_ contour representations and showed that prosodic information alone achieved above-chance classification performance, indicating that dialect-specific intonational patterns are present across Arabic varieties. Their results further suggested that prosodic contour realization is influenced not only by sentence type but also by information structure and discourse context. However, their study approached prosody primarily from the perspective of dialect discrimination and sociophonetic variation rather than computational modeling of communicative functions such as focus.

A more direct application of computational modeling to Arabic intonation was done in Alzaidi et al. (2023), which employed PENTAtrainer to model focus intonation in Emirati Arabic. The modeling work achieved a high correspondence between observed and predicted *F*_0_ contours. Importantly, it was shown that beside focus, functions like stress, prosodic word, etc. also contributed to the shaping of sentential *F*_0_ contours. These findings highlight the value of computational approaches for quantifying the relative contribution of different prosodic factors and for uncovering aspects of intonational structure that may not be readily apparent from acoustic analyses alone.

In light of these findings, the present study extends previous phonetic and computational research by investigating focus realization in Qassimi Arabic. The study has two primary objectives. First, it examines whether focus is associated with systematic prosodic variation across on-focus, pre-focus, and post-focus regions in terms of *F*_0_, intensity, and duration. Second, it employs a PENTA-based modeling framework to explore the functional factors that jointly contribute to the formation of intonation in Qassimi Arabic, including focus, stress, syllable weight, position of word-level stress, and prosodic word, with the aim to determine their relative importance in shaping *F*_0_ contours.

### 1.4 Qassimi Arabic

Qassimi Arabic (QA) is a Najdi dialect spoken in Al-Qassim, a region in central Saudi Arabia (Figure 2). Within the Najdi dialect continuum, QA belongs to the Shammari group and is considered a mixed northern-central Najdi variety (Ingham, 1994; Johnstone, 1967). It belongs to a cluster of dialects often characterized as relatively conservative due to their historical geographic isolation and comparatively limited contact with other Arabic varieties (Ingham, 1994; Holes, 1995; Alnuqaydan, 2023).

**Figure 2:**
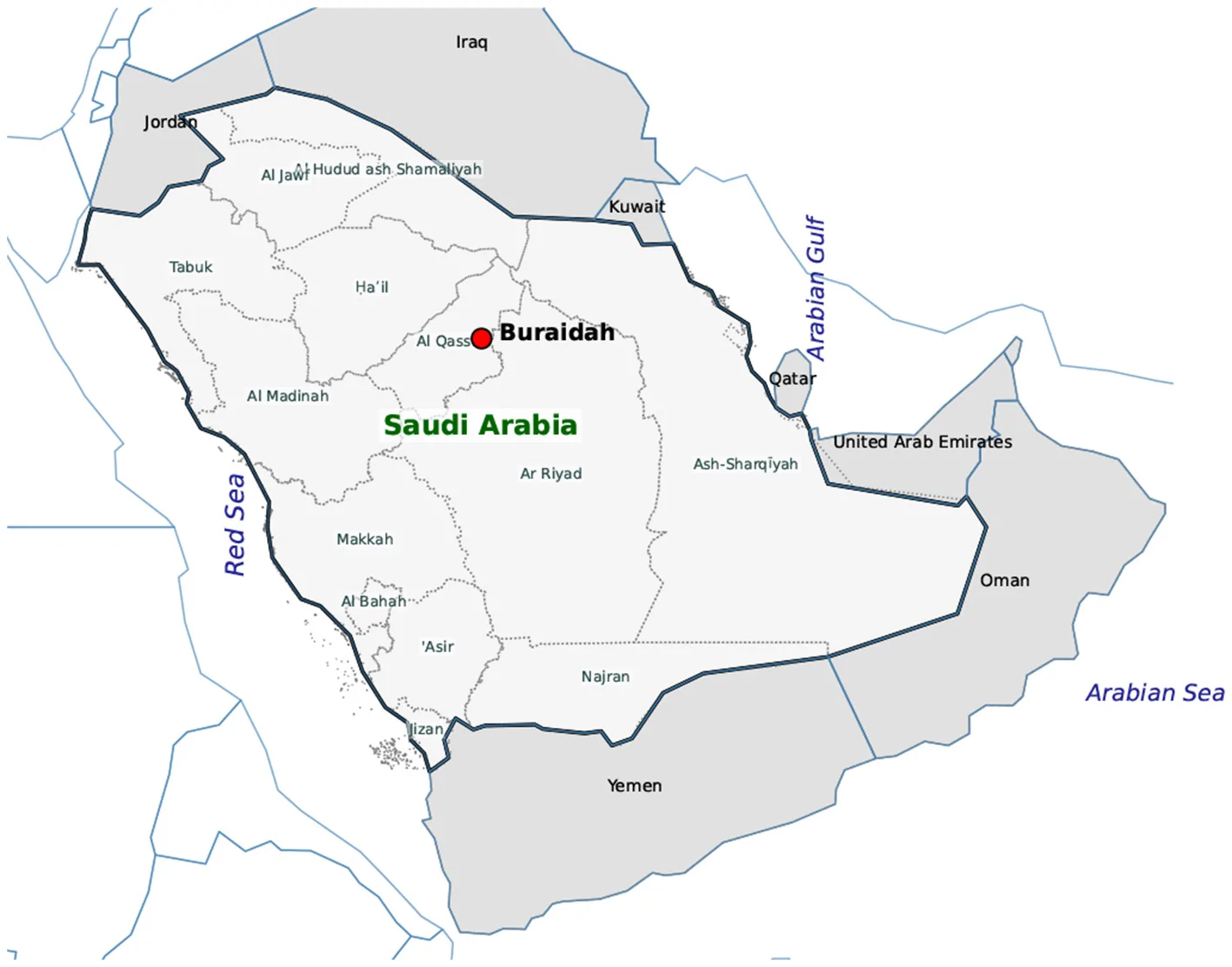
Map of Saudi Arabia showing Najd, Al-Qassim, and the data-collection site in Buraidah.

While it shares many features with other Najdi dialects (Alqahtani, 2014), QA is phonetically and phonologically distinct in several ways (Al Motairi, 2015). For example, it differs from several neighboring Najdi varieties in exhibiting a historical merger between the interdental emphatic fricative /ð^ʔ^/ and the emphatic dental stop /d^ʔ^/, preserving only the fricative realization (Ingham, 1994; Al Motairi, 2015; Alnuqaydan, 2023). With regards to the syllable structure and prosodic-word organization, QA syllables are maximally bimoraic and that the dialect systematically restricts trimoraic structures (see Al Motairi, 2015 for more details). Unlike several neighboring Najdi dialects that tolerate non-final superheavy syllables of the shape CVVC and CVCC, QA strongly disfavors such structures and frequently repairs them through vowel epenthesis (Alqahtani, 2014; Al Motairi, 2015)

Consistent with broader Najdi Arabic patterns, QA exhibits a quantity-sensitive default-to-left stress system in which stress assignment is determined by the interaction of syllable weight and lexical syllable position (Al Motairi, 2015; Alrashed, 2018). More specifically, primary stress falls on the rightmost heavy syllable, while in the absence of heavy syllables stress defaults to the initial syllable. In QA, syllables of the shape CVVC, CVCC, and CVV are treated as heavy regardless of their position within the lexical word, whereas CVC syllables attract stress only in non-final position. Stress is therefore largely predictable and metrically governed rather than lexically contrastive, reflecting systematic interactions between syllable structure, moraic weight, and prosodic-word organization. Acoustic analyses by Alnuqaydan (2023) demonstrate that stressed syllables in QA are consistently associated with longer duration, greater intensity, higher *F*_0_, and higher F1 values relative to unstressed syllables. His study further shows that stress interacts closely with a range of phonological processes, including vowel epenthesis, syllable restructuring, and the avoidance of illicit triconsonantal clusters, particularly in contexts involving superheavy syllables and stress placement near the right edge of the word. Collectively, these patterns suggest that stress, syllable weight, lexical syllable position, and prosodic-word organization may interact closely in shaping the prosodic system of QA.

Like many spoken Arabic dialects, QA permits limited constituent reordering for discourse-related purposes. Nevertheless, canonical SVO declaratives remain highly frequent in conversational speech, as exemplified in (4.1) (Alzaidi, 2014; Alzaidi et al., 2023; Alzaidi, 2024).

(4) 1. Sarah ʔiktebat ʔad-dars
          Sarah write-past.3sg.fem the-lesson
         ‘Sarah wrote the lesson.'

(5) 1. What happened?
      2. Sarah ʔiktebat ʔad-dars
          Sarah write-past.3sg.fem the-lesson
          ‘Sarah wrote the lesson.'

In addition, QA also permits alternative constituent orders such as Verb-Subject-Object (VSO) (6.1) and Object-Verb-Subject (OVS) (7.1), each associated with distinct discourse-related interpretations. VSO constructions are commonly associated with event-oriented or predicate-prominent readings, whereas OVS structures may be used to increase the discourse salience or contrastive interpretation of the object constituent. Unlike Modern Standard Arabic, where contrastive focus may be expressed through syntactic constructions such as constituent fronting or cleft-like structures, QA, like many spoken Arabic dialects, commonly realizes both information focus and contrastive focus *in situ*, with the intended interpretation derived primarily from prosody and discourse context.

QA allows *in-situ* realization of information focus (8, 9) and contrastive focus (10, 11). In many cases, the same canonical SVO declarative structure can be maintained across neutral focus, information-focus, and contrastive-focus contexts, with the intended interpretation recoverable primarily through prosodic modulation and discourse context rather than overt syntactic displacement.

(6) 1. ʔiktebat Sarah ʔad-dars
          write-past.3sg.fem Sarah the-lesson
          ‘Sarah wrote the lesson.'

(7) 1. ʔad-dars ʔiktebat Sarah
          the-lesson write-past.3sg.fem Sarah
          ‘Sarah wrote the lesson.'

(8) 1. Who wrote the lesson?                    *Information focus on subject*
      2. Sarah ʔiktebat ʔad-dars
          Sarah write-past.3sg.fem the-lesson
         ‘Sarah wrote the lesson.'

(9) 1. What did Sarah write?                 *Information focus on object*
      2. Sarah ʔiktebat ʔad-dars
          Sarah write-past.3sg.fem the-lesson
         ‘Sarah wrote the lesson.'

(10) 1. Who wrote the lesson? Manal?   *Contrastive focus on subject*
        2. Sarah ʔiktebat ʔad-dars
            Sarah write-past.3sg.fem the-lesson
           ‘Sarah wrote the lesson.'

(11) 1. What did Sarah write? The letter?     *Contrastive focus on object*
        2. Sarah ʔiktebat ʔad-dars
            Sarah write-past.3sg.fem the-lesson
           ‘Sarah wrote the lesson.'

QA's preference for *in-situ* focus realization within canonical declarative structures makes it particularly well-suited for investigating how prosodic structure contributes to the acoustic realization of information structure. These properties provide a controlled environment for examining how focus-related prosodic variation is acoustically realized in the absence of obligatory syntactic displacement. Accordingly, QA offers an effective testing ground for investigating how focus-related prosodic functions can be acoustically analyzed and computationally modeled under syntactically controlled conditions.

### 1.5 Research questions

Although previous studies have documented focus-related prosodic variation in several Arabic dialects, less is known about how focus is realized in Qassimi Arabic and how it contributes to the computational representation of intonation. The present study therefore addresses the following research questions:

1. How is focus realized prosodically in Qassimi Arabic? Specifically:
  (a) Does focus influence *F*_0_, intensity, and duration in on-focus, pre-focus, and post-focus regions?
  (b) Do information focus and contrastive focus differ in their acoustic realization?

  2. Can the prosodic effects of focus in Qassimi Arabic be captured through computational modeling of intonation? Specifically:
  (a) To what extent can a PENTA-based model account for the observed *F*_0_ contours of Qassimi Arabic speech?
  (b)What functional factors contribute to the computational modeling of intonation in Qassimi Arabic, and what is their relative importance?

## 2 Production experiment

### 2.1 Method

#### 2.1.1 Materials

Similar to previous studies on Arabic dialects (Alzaidi, 2022; Alzaidi et al., 2023, 2025), this study used short declarative sentences in the production experiment. The sentences examined acoustic measurements in three positions: sentence-initial, sentence-penultimate, and sentence-final. The words were selected to contain sonorant sounds and no coda consonants. This made fundamental frequency (*F*_0_) alignment more accurate and allowed reliable acoustic measurements. Three sentence groups (12) were prepared. Each group contained four sentences. The underlined words (Malikah, Muneerah, Dima, and Lina) were the target words. They carried the focus conditions in the experiment. The target words differed in word length, ranging from two to three syllables. They also differed in lexical stress, with either word-initial or word-penultimate stress. Four focus conditions were tested: neutral focus, sentence-initial focus, sentence-penultimate focus, and sentence-final focus. The syllable boundaries are indicated by a dot, and the stressed syllables are in bold.

(12) 1. Ma.l**ī**.kah/Mu.n**ī**.rah/D**ī**.ma/L**ī**.na   **ḥa**.mat     May.**mū**.nah min Na.**wāl**.
            Malikah/Muneerah/Dima/Lina    protected Maymuunah from Nawal.
            ‘Malikah/Muneerah/Dima/Lina    protected     Maymuunah from Nawal.'
             4 words × 3 foci × 5 repetitions = 60
        2. May.**mū**.nah **jā**.bat     Ma.l**ī**.kah/Mu.n**ī**.rah/D**ī**.ma/L**ī**.na li Na.**wāl**.
             Maymuunah  brought   Malikah/Muneerah/Dima/Lina to Nawal.
            ‘Maymuunah   brought   Malikah/Muneerah/Dima/Lina to Nawal.'
             4 words × 3 foci × 5 repetitions = 60
         3. May.**mū**.nah     **wad**.dat     Na.**wāl**     li Ma.l**ī**.kah/Mu.n**ī**.rah/D**ī**.ma/L**ī**.na.
             Maymuunah     took     Nawal     to     Malikah/Muneerah/Dima/Lina.‘Maymuunah     took     Nawal     to
             Malikah/Muneerah/Dima/Lina.'
             4 words × 3 foci × 5 repetitions = 60

The target sentences were embedded in specific question-answer paradigms. Sentences were produced under three focus conditions, as exemplified in (13), (14), and (15). The focus conditions were elicited by providing participants with prompt questions designed to direct attention to specific key words (first sentences in (13), (14), and (15). This method, successfully implemented in prior studies (Cooper et al., 1985; Xu, 1999; Chahal, 2001; Xu and Xu, 2005; Xu, 2011b; Alzaidi et al., 2019; Alzamil and Hellmuth, 2022; Alzaidi et al., 2025), ensured consistency in eliciting the intended focus conditions.

(13) 1. waiš ʔassālfah?
            what the-story
          ‘What is the story?'
       2. Malīkah ḥamat Maymūnah min Nawāl. *Neutral focus*
           Malikah protect-PAST.3SG.FEM Maymuunah from Nawal
          ‘Malikah protected Maymuunah from Nawal.'

(14) 1. mīn ḥama Maymūnah min Nawāl?
            who protected Maymuunah from Nawal
           ‘Who protected Maymuunah from Nawal?'
        2. Malī*kah* ḥamat Maymūnah min Nawāl. *Information focus*
            Malikah protect-PAST.3SG.FEM Maymuunah from Nawal
           ‘Malikah protected Maymuunah from Nawal.'

(15) 1. mīn ḥama Maymūnah min Nawāl? Jawāhir?
            who protected Maymuunah from Nawal Jawahir
           ‘Who protected Maymuunah from Nawal? Jawahir?'
        2. Malī*kah* ḥamat Maymūnah min Nawāl. *Contrastive focus*
            Malikah protect-PAST.3SG.FEM Maymuunah from Nawal
           ‘Malikah protected Maymuunah from Nawal.'

Each question–answer pair was preceded by an anecdote that provided the appropriate discourse context. One anecdote at a time was presented on the computer screen for the subject to read silently. Once the subject had finished reading the anecdote, they were asked to read the target sentence as an answer to the prompt question. Both the question and the target answer were displayed on the screen. A sample anecdote is provided in (16).

(16) 1. Malīkah wa Maymūnah wa Nawāl ẖawāt. Nawāl tebi taḍrib
            Maymūnah, lākin Malīkah ḥamatha.
            Malikah and Maymuunah and Nawal sisters. Nawal wanted
            hit Maymuunah but Malikah protected her.
           ‘Malikah, Maymuunah, and Nawal are sisters. Nawal
            wanted to hit Maymuunah, but Malikah protected her.'

#### 2.1.2 Subjects

Ten native speakers of Qassimi Arabic, aged 21–25, voluntarily participated in the study. All were female and were born and raised in Buraidah in the Al-Qassim region. None reported any speech or hearing disorders, and all spoke Qassimi Arabic fluently, without any noticeable accent differing from the variety spoken in Buraidah. Before taking part, the participants were informed about the purpose and procedures of the study, the voluntary nature of their participation, and their right to withdraw at any stage without penalty. All participants provided written informed consent for the recording and analysis of their speech data. Their personal information was kept confidential, and the data were anonymized for analysis and reporting.

#### 2.1.3 Recording procedure

Recording took place in a sound-treated booth at the Linguistics Laboratory in the Department of English Language at King Saud University, Riyadh, Saudi Arabia. Participants were seated in front of a MacBook Pro displaying test materials presented via PowerPoint slides. A head-worn microphone, connected to the laptop through a MicPort Pro USB preamplifier, captured the utterances, which were recorded in Audacity(R) (version 3.2.3) (Audacity Team, MuseCY SM Ltd. Limassol, Cyprus) at a 44,100 Hz sampling rate and 16-bit resolution (Audacity Team, 2022).

Similar to previous studies (Alzaidi et al., 2023, 2025), the participants first read a short anecdote silently. The anecdote reflected the natural linguistic patterns of Qassimi Arabic. After that, they answered a comprehension question about a specific detail in the anecdote. The question was presented through a loudspeaker. It had been recorded by a 23-year-old female native speaker of Qassimi Arabic. The recorded prompt questions were used only to elicit the target responses and were not included in the acoustic-analysis files. The same loudspeaker and playback-volume setting were used for all participants and recording sessions to maintain consistent prompt presentation. The sentences were presented in a random order. The participants were asked to speak naturally and maintain a normal speech rate. They were also instructed to avoid unnecessary pauses and disfluencies. Thirty five minutes was the average time of the recording sessions.

#### 2.1.4 Acoustic measurements and statistical analyses

The tokens were manually labeled in Praat (Boersma and Weenink, 2023). Each stressed syllable was defined as extending from the onset closure to the end of the coda. In open syllables, where there was no coda, the stressed syllable ended at the vowel offset (Alzaidi et al., 2019; Alzaidi, 2022; Alzaidi et al., 2023, 2025). After manual labeling, the acoustic measurements listed in Item 17 were extracted with ProsodyPro, a Praat script used for large-scale prosodic analysis (Xu, 2013).

(17) Acoustic parameters

1. Excursion size (st): Pitch range in semitones between the maximum and minimum *F*_0_.
2. Maximum *F*_0_ (Hz): Highest *F*_0_.
3. Minimum *F*_0_ (Hz): Lowest *F*_0_.
4. Mean *F*_0_ (Hz): Average *F*_0_ value across the syllable.
5. Mean intensity (dB): The average intensity of the stressed syllable.
6. Duration (ms): The length of the stressed syllable.

The acoustic measurements were analyzed using linear mixed-effects models implemented in the lme4 package in R (Bates et al., 2015; R Core Team, 2019). Separate models were fitted for each sentence position and acoustic measure. In each model, focus condition (neutral focus, information focus, and contrastive focus) was entered as a fixed effect, while speaker and sentence were included as crossed random grouping factors with by-focus random slopes. For each acoustic measure, the significance of focus was evaluated by comparing a full model containing the fixed effect of focus with an otherwise identical null model using a likelihood-ratio test under maximum-likelihood estimation. When the omnibus test was significant, Tukey-adjusted pairwise comparisons were performed using the multcomp package (Hothorn et al., 2008). Model singularity and convergence were examined for all fitted models. The full R code is provided in Appendix A, and Appendix B presents boxplots for all acoustic measurements.

As explained in Section 1.2, the pre-focus and post-focus regions measured here correspond to the same word positions across all three focus conditions, including neutral focus; a “pre-focus” or “post-focus” label therefore reflects the absence of local focus-driven modification at that position, not the presence of a focused constituent elsewhere in the neutral-focus sentence.

### 2.2 Results

#### 2.2.1 The Global *F*_0_ Curve

Before examining individual acoustic measures, a qualitative assessment of focus effects is conducted using SS-ANOVA plots of time-normalized *F*_0_ contours (Figures 3–5). These plots allow visualization of global intonational patterns across focus conditions and facilitate the identification of potential focus-related modifications in on-focus, pre-focus, and post-focus regions. Inspection of the contours reveals systematic differences across focus conditions, particularly in the realization of the focused constituent and the prosodic behavior of following material. The most salient patterns are described below.

**Figure 3:**
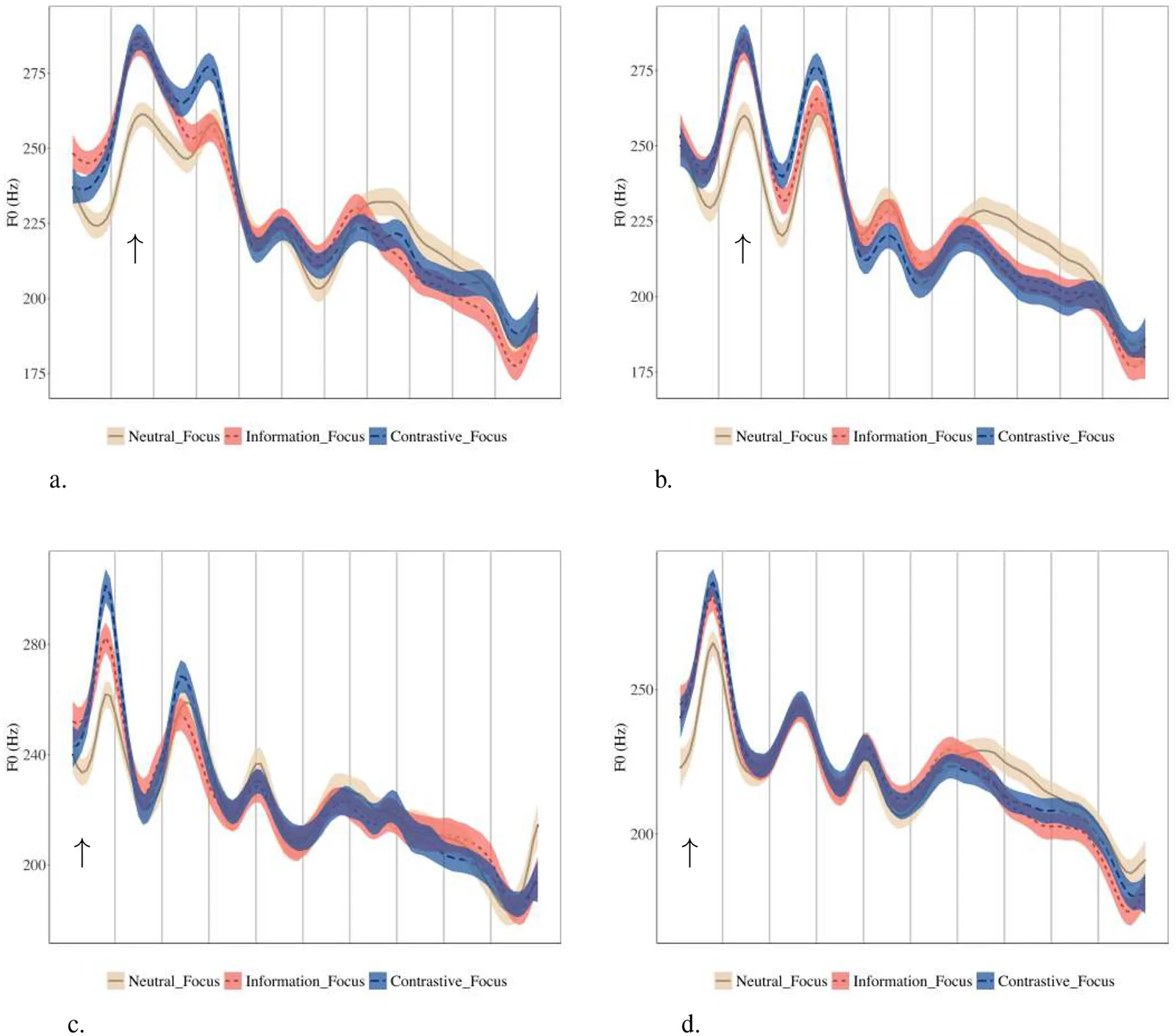
SS-ANOVA plots of time-normalized *F*_0_ contours for sentence-initial focus across the four target-word variants. Solid lines represent estimated mean *F*_0_ trajectories, and the surrounding ribbons represent 95% confidence intervals. Vertical lines mark syllable boundaries. Stressed syllables are shown in boldface, focused words are underlined, and upward arrows indicate the stressed syllables of the focused lexical items. Separation between confidence ribbons is treated as descriptive evidence of contour divergence rather than as a substitute for the mixed-effects analyses reported in Section 2.2.2. **(a)** /ma.l**ī**.kah ḥa.mat may.**mu**.nah min na.**wāl**/. **(b)** /mu.n**ī**.rah ḥa.mat may.**mu**.nah min na.**wāl**/. **(c)** /d**ī**.ma ḥa.mat may.**mu**.nah min na.**wāl**/. **(d)** /l**ī**.na ḥa.mat may.**mu**.nah min na.**wāl**/.

**Figure 4:**
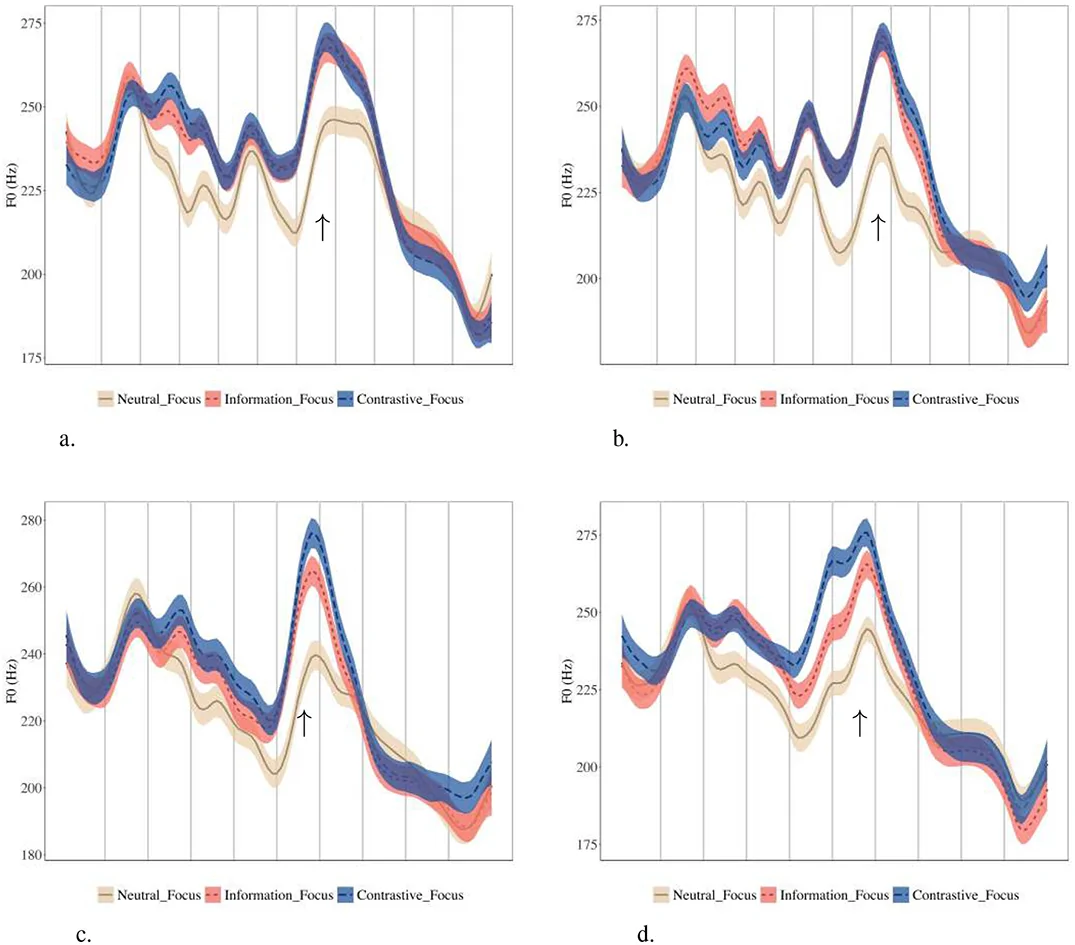
SS-ANOVA plots of time-normalized *F*_0_ contours for sentence-penultimate focus across the four target-word variants. Solid lines represent estimated mean *F*_0_ trajectories, and the surrounding ribbons represent 95% confidence intervals. Vertical lines mark syllable boundaries. Stressed syllables are shown in boldface, focused words are underlined, and upward arrows indicate the stressed syllables of the focused lexical items. Separation between confidence ribbons is treated as descriptive evidence of contour divergence rather than as a substitute for the mixed-effects analyses reported in Section 2.2.2. **(a)** /may.**mu**.nah ʒ**ā**.bat ma.l**ī**.kah li na.**wāl**/. **(b)** /may.**mu**.nah ʒ**ā**.bat mu.n**ī**.rah li na.**wāl**/. **(c)** /may.**mu**.nah ʒ**ā**.bat d**ī**.ma li na.**wāl**/. **(d)** /may.**mu**.nah ʒ**ā**.bat l**ī**.na li na.**wāl**/.

**Figure 5:**
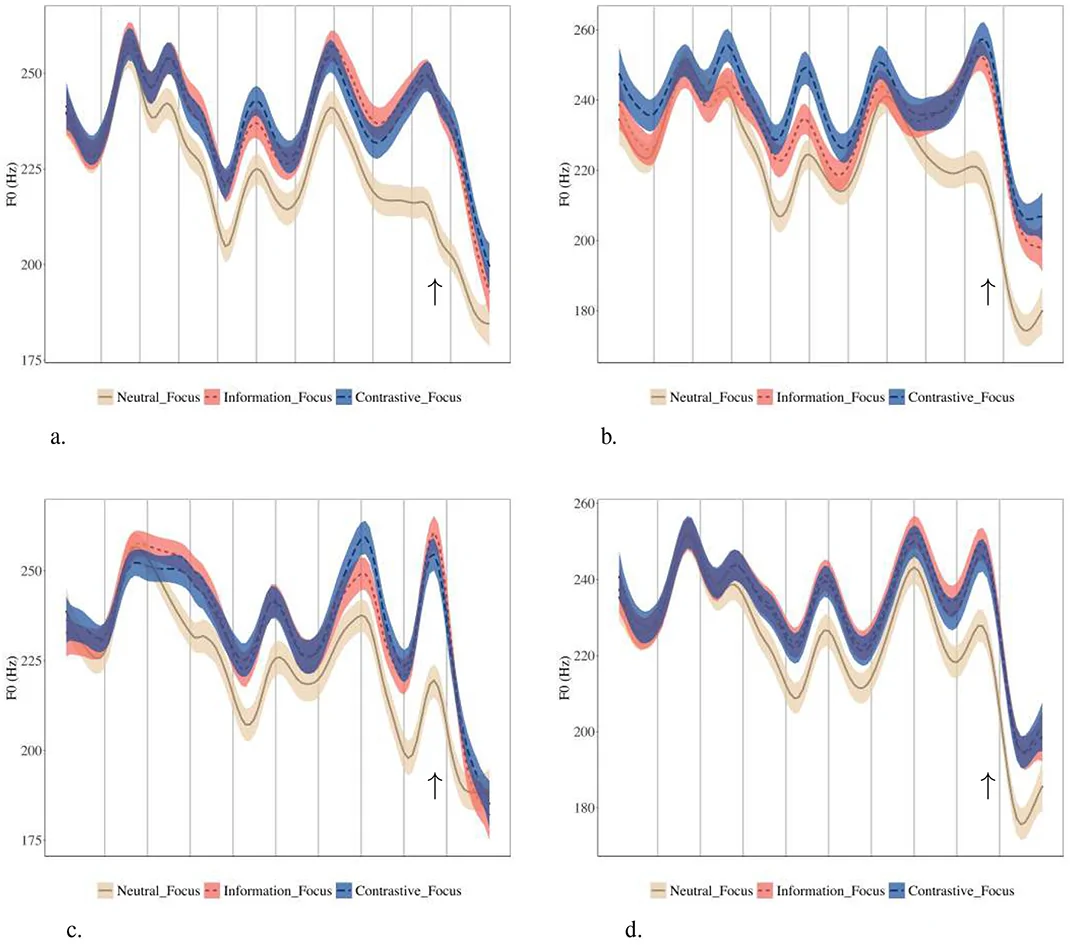
SS-ANOVA plots of time-normalized *F*_0_ contours for sentence-final focus across the four target-word variants. Solid lines represent estimated mean *F*_0_ trajectories, and the surrounding ribbons represent 95% confidence intervals. Vertical lines mark syllable boundaries. Stressed syllables are shown in boldface, focused words are underlined, and upward arrows indicate the stressed syllables of the focused lexical items. Separation between confidence ribbons is treated as descriptive evidence of contour divergence rather than as a substitute for the mixed-effects analyses reported in Section 2.2.2. **(a)** /may.**mu**.nah **wad**.dat na.**wāl** li ma.l**ī.**kah/. **(b)** /may.**mu**.nah **wad**.dat na.**wāl** li mu.n**ī**.rah/. **(c)** /may.**mu**.nah **wad**.dat na.**wāl** li d**ī**.ma/. **(d)** /may.**mu**.nah **wad**.dat na.**wāl** li l**ī**.na/.

In the on-focus region, the *F*_0_ contours differ across focus conditions. Both information focus and contrastive focus raise *F*_0_ on the focused word. In some cases, contrastive focus shows higher peaks than information focus, as shown in Figures 3c, 4c, d. The non-overlapping confidence intervals suggest that this increase is a reliable marker of prosodic prominence.

In the post-focus region, the time-normalized contours show visually narrower *F*_0_ movement following the focused word in some panels. This apparent narrowing is most visible when the stressed syllable of the penultimate content word serves as the post-focus anchor, as seen in Figures 3a–d. In contrast, the pre-focus region shows little evidence of systematic modification. The *F*_0_ contours remain largely unaffected. This apparent asymmetry between relatively stable pre-focus contours and localized post-focus modification suggests that focus-related adjustments in Qassimi Arabic are primarily realized through enhancement of the focused constituent and modification of following material rather than through anticipatory changes in preceding regions. Since these observations are based on smoothed group contours, they are treated as exploratory visual patterns rather than as statistical confirmation of post-focus compression. Their interpretation is therefore evaluated against the mixed-effects analyses reported below.

#### 2.2.2 Quantitative results

##### 2.2.2.1 On-focus region

Table 1 presents descriptive statistics, including means and standard deviations, for the six acoustic parameters listed in 17 across the three focus conditions, together with the corresponding linear mixed-effects model results. Overall, focus is realized primarily through modifications of *F*_0_, whereas intensity and duration show comparatively weak effects. For most pitch-related measures, focused words have higher values than their neutral-focus counterparts. Contrastive focus often shows the highest values, although this pattern is not consistent across all measures and sentence positions.^1^

**Table 1:** Mean on-focus acoustic measurements across focus conditions and results from the linear mixed-effects models.

Measurements	NF	IF	CF
Initial focus			
Excursion size (st.)	*M* = 4.31 (2.56)	*M* = 5.59 (3.04)	*M* = 6.02 (3.27)
χ^2^(2) = 6.67, ***p*** **= 0.036**			
Maximum *F0* (Hz)	*M* = 274.88 (47.36)	*M* = 307.60 (48.30)	*M* = 318.80 (57.06)
χ^2^(2) = 7.72, ***p*** **= 0.021**			
Mean *F0* (Hz)	*M* = 243.62 (38.26)	*M* = 262.59 (40.48)	*M* = 265.51 (47.35)
χ^2^(2) = 4.37, *p* = 0.112			
Minimum *F0* (Hz)	*M* = 213.81 (30.60)	*M* = 223.13 (34.59)	*M* = 224.97 (34.79)
χ^2^(2) = 2.93, *p* = 0.231			
Mean intensity (dB)	*M* = 83.03 (2.92)	*M* = 83.25 (3.29)	*M* = 83.10 (3.48)
χ^2^(2) = 0.46, *p* = 0.794			
Duration (ms)	*M* = 216.08 (58.48)	*M* = 224.56 (59.46)	*M* = 216.04 (55.86)
χ^2^(2) = 2.06, *p* = 0.357			
Penultimate focus			
Excursion size (st.)	*M* = 3.34 (3.04)	*M* = 4.13 (2.47)	*M* = 4.93 (3.23)
χ^2^(2) = 5.49, *p* = 0.064			
Maximum *F0* (Hz)	*M* = 253.98 (51.00)	*M* = 286.77 (43.48)	*M* = 299.01 (54.52)
χ^2^(2) = 8.93, ***p*** **= 0.012**			
Mean *F0* (Hz)	*M* = 233.10 (36.29)	*M* = 262.90 (34.83)	*M* = 269.57 (40.58)
χ^2^(2) = 11.68, ***p*** **= 0.003**			
Minimum *F0* (Hz)	*M* = 208.58 (31.35)	*M* = 225.31 (28.88)	*M* = 224.27 (33.52)
χ^2^(2) = 10.87, ***p*** **= 0.004**			
Mean intensity (dB)	*M* = 80.12 (3.48)	*M* = 81.36 (3.96)	*M* = 81.36 (4.02)
χ^2^(2) = 2.86, *p* = 0.239			
Duration (ms)	*M* = 176.78 (44.95)	*M* = 189.27 (43.47)	*M* = 200.82 (54.81)
χ^2^(2) = 5.82, *p* = 0.055			
Final focus			
Excursion size (st.)	*M* = 4.28 (3.71)	*M* = 4.32 (2.33)	*M* = 4.44 (2.94)
χ^2^(2) = 0.14, *p* = 0.934			
Maximum *F0* (Hz)	*M* = 232.43 (44.02)	*M* = 272.14 (47.55)	*M* = 271.38 (49.52)
χ^2^(2) = 5.60, *p* = 0.061			
Mean *F0* (Hz)	*M* = 212.20 (39.94)	*M* = 248.44 (35.57)	*M* = 247.91 (35.69)
χ^2^(2) = 6.34, ***p*** **= 0.042**			
Minimum *F0* (Hz)	*M* = 183.33 (37.77)	*M* = 210.45 (23.85)	*M* = 209.23 (28.33)
χ^2^(2) = 7.29, ***p*** **= 0.026**			
Mean intensity (dB)	*M* = 78.26 (3.86)	*M* = 81.34 (4.07)	*M* = 81.07 (4.42)
χ^2^(2) = 5.94, *p* = 0.051			
Duration (ms)	*M* = 193.88 (43.58)	*M* = 206.43 (50.75)	*M* = 207.63 (50.16)
χ^2^(2) = 4.69, *p* = 0.096			

Significant *p*-values are shown in bold. Standard deviations are given in parentheses.

The clearest effects on pitch range and pitch height occur in the initial and penultimate positions. Excursion size increases from neutral focus to information focus and contrastive focus in the initial position, where the effect of focus is significant [χ^2^(2) = 6.67, *p* = 0.036]. A similar numerical tendency occurs in the penultimate position, but the omnibus effect does not reach significance [χ^2^(2) = 5.49, *p* = 0.064]. No corresponding effect is found in the final position [χ^2^(2) = 0.14, *p* = 0.934]. Maximum *F*_0_ also differs significantly across focus conditions in the initial [χ^2^(2) = 7.72, *p*=0.021] and penultimate positions [χ^2^(2) = 8.93, *p* = 0.012], whereas the effect in the final position falls short of significance [χ^2^(2) = 5.60, *p* = 0.061]. These findings indicate that focus raises pitch peaks and expands pitch range most clearly in non-final positions.

Mean *F*_0_ shows a position-dependent pattern. In the sentence-initial position, mean *F*_0_ is numerically higher under information and contrastive focus than under neutral focus, but the omnibus effect is not significant [χ^2^(2) = 4.37, *p* = 0.112]. Significant effects are found in both the penultimate position [χ^2^(2) = 11.68, *p* = 0.003] and the final position [χ^2^(2) = 6.34, *p* = 0.042]. Minimum *F*_0_ also differs significantly across focus conditions in the penultimate [χ^2^(2) = 10.87, *p* = 0.004] and final positions [χ^2^(2) = 7.29, *p* = 0.026], but not in the initial position [χ^2^(2) = 2.93, *p* = 0.231]. Together, these results show that focus affects not only pitch peaks but also the overall pitch register, particularly in penultimate and final positions.

By contrast, intensity and duration provide weaker evidence of focus marking. Mean intensity does not show a significant effect in the initial [χ^2^(2) = 0.46, *p* = 0.794] or penultimate position [χ^2^(2) = 2.86, *p* = 0.239], and the effect in the final position narrowly misses significance [χ^2^(2) = 5.94, *p* = 0.051]. Duration is likewise non-significant in the initial [χ^2^(2) = 2.06, *p* = 0.357], penultimate [χ^2^(2) = 5.82, *p* = 0.055], and final positions [χ^2^(2) = 4.69, *p* = 0.096].

The *post-hoc* pairwise comparisons in Table 2 show that the significant omnibus effects arise primarily from differences between the focused conditions and neutral focus. Most direct comparisons between information focus and contrastive focus do not reach statistical significance, although contrastive focus has a significantly higher maximum *F*_0_ than information focus in the penultimate position.

**Table 2:** Tukey-adjusted pairwise comparisons for significant focus effects on the stressed syllables of focused words in the initial, penultimate, and final positions.

Measurement	Contrast	Estimate	SE	95% CI	Adjusted *p*
Initial focus					
Excursion size (st.)	IF–NF	1.28	0.46	[0.21, 2.35]	**0.014**
Excursion size (st.)	CF–NF	1.70	0.56	[0.42, 2.98]	**0.006**
Excursion size (st.)	CF–IF	0.42	0.28	[–0.24, 1.07]	0.284
Maximum *F0* (Hz)	IF–NF	32.72	9.72	[10.95, 54.50]	**0.002**
Maximum *F0* (Hz)	CF–NF	43.92	12.58	[13.55, 69.91]	**0.002**
Maximum *F0* (Hz)	CF–IF	11.2	4.46	[–0.98, 18.99]	0.083
Penultimate focus					
Maximum *F0* (Hz)	IF–NF	32.78	11.51	[6.33, 59.24]	**0.011**
Maximum *F0* (Hz)	CF–NF	45.03	12.62	[16.00, 74.06]	**< 0.001**
Maximum *F0* (Hz)	CF–IF	12.24	5.24	[0.20, 24.29]	**0.045**
Mean *F0* (Hz)	IF–NF	29.79	7.08	[13.44, 46.15]	**< 0.001**
Mean *F0* (Hz)	CF–NF	36.46	7.75	[18.56, 54.37]	**< 0.001**
Mean *F0* (Hz)	CF–IF	6.67	3.77	[–2.03, 15.37]	0.168
Minimum *F0* (Hz)	IF–NF	16.73	4.62	[5.93, 27.54]	**< 0.001**
Minimum *F0* (Hz)	CF–NF	15.69	3.76	[6.92, 24.47]	**< 0.001**
Minimum *F0* (Hz)	CF–IF	–1.04	3.78	[–9.88, 7.80]	0.959
Final focus					
Mean *F0* (Hz)	IF–NF	36.24	13.69	[5.49, 66.99]	**0.017**
Mean *F0* (Hz)	CF–NF	35.71	15.24	[1.48, 69.94]	**0.039**
Mean *F0* (Hz)	CF–IF	–0.53	3.21	[–7.74, 6.68]	0.982
Minimum *F0* (Hz)	IF–NF	27.12	8.84	[6.99, 47.25]	**0.005**
Minimum *F0* (Hz)	CF–NF	25.90	9.56	[4.13, 47.66]	**0.015**
Minimum *F0* (Hz)	CF–IF	–1.22	2.70	[–7.36, 4.93]	0.882

Estimates represent the first condition minus the second condition. Confidence intervals and *p*-values are simultaneously adjusted using the single-step procedure. Only acoustic measures with a significant omnibus effect of focus are included. NF, neutral focus; IF, information focus; CF, contrastive focus. Bold values indicate statistically significant *p*-values (*p* < 0.05).

In the initial position, both information focus and contrastive focus have significantly larger excursion sizes than neutral focus. The estimated increases are 1.28 st for information focus relative to neutral focus [95% CI [0.21, 2.35], adjusted *p* = 0.014] and 1.70 st for contrastive focus relative to neutral focus [95% CI [0.42, 2.98], adjusted *p* = 0.006]. Both focused conditions also have significantly higher maximum *F*_0_ values than neutral focus. Information focus exceeds neutral focus by 32.72 Hz [95% CI [10.95, 54.50], adjusted *p* = 0.002], while contrastive focus exceeds neutral focus by 41.73 Hz [95% CI [13.55, 69.91], adjusted *p* = 0.002]. The differences between information focus and contrastive focus are not significant for either excursion size or maximum *F*_0_.

The strongest and most extensive effects occur in the penultimate position. Both information focus and contrastive focus have significantly higher maximum *F*_0_, mean *F*_0_, and minimum *F*_0_ values than neutral focus. For maximum *F*_0_, information focus exceeds neutral focus by 32.78 Hz [95% CI [6.33, 59.24], adjusted *p* = 0.011], and contrastive focus exceeds neutral focus by 45.03 Hz [95% CI [16.00, 74.06], adjusted *p* < 0.001]. Contrastive focus also has a significantly higher maximum *F*_0_ than information focus, with an estimated difference of 12.24 Hz [95% CI [0.20, 24.29], adjusted *p* = 0.045]. For mean *F*_0_, both information focus and contrastive focus are significantly higher than neutral focus, with estimated differences of 29.79 Hz and 36.46 Hz, respectively. Similarly, minimum *F*_0_ is significantly higher under both information focus and contrastive focus than under neutral focus. However, information focus and contrastive focus do not differ significantly in mean or minimum *F*_0_.

The final position shows a more restricted pattern. Significant differences occur only for mean *F*_0_ and minimum *F*_0_. Both information focus and contrastive focus have significantly higher mean *F*_0_ values than neutral focus. The estimated differences are 36.24 Hz for information focus [95% CI [5.49, 66.99], adjusted *p* = 0.017] and 35.71 Hz for contrastive focus [95% CI [1.48, 69.94], adjusted *p* = 0.039]. Both focused conditions also have significantly higher minimum *F*_0_ values than neutral focus. Information focus exceeds neutral focus by 27.12 Hz [95% CI [6.99, 47.25], adjusted *p* = 0.005], and contrastive focus exceeds neutral focus by 25.90 Hz [95% CI [4.13, 47.66], adjusted *p* = 0.015]. Information focus and contrastive focus do not differ significantly in either mean or minimum *F*_0_.

Overall, the pairwise comparisons show that focus marking in the on-focus region is realized primarily through raising the *F*_0_ contour relative to neutral focus. Pitch-range expansion is also evident in the initial position, where both focused conditions have larger excursion sizes than neutral focus. The most extensive effects occur in the penultimate position, where focus affects maximum, mean, and minimum *F*_0_. The final position shows a narrower pattern involving mean and minimum *F*_0_ only. Most direct comparisons between information focus and contrastive focus do not reach statistical significance; however, contrastive focus has a significantly higher maximum *F*_0_ than information focus in the penultimate position. The analyses therefore detected a measure-specific difference between the two focus types, but they did not detect a consistent distinction across measures and sentence positions.

##### 2.2.2.2 Post-focus region

The post-focus region was analyzed in two ways. The first analysis included the stressed syllables of all post-focus content words. This showed the general post-focus pattern. The second analysis focused only on the stressed syllable of the sentence-penultimate word. This word was selected because the SS-ANOVA results showed that it was the main site of post-focus effects in the sentence-initial focus condition (see Section 2.2.1). These two analyses therefore provide both a broad and a local view of post-focus prosody.^2^

Table 3 presents the descriptive statistics and linear mixed-effects model results for the post-focus regions following sentence-initial and sentence-penultimate focus. In the post-focus region following sentence-initial focus, focus condition significantly affects mean intensity [χ^2^(2) = 6.31, *p* = 0.043]. Mean intensity is highest under neutral focus (*M* = 77.22 dB, SD = 2.93) and lower under information focus (*M* = 74.59 dB, SD = 4.94) and contrastive focus (*M* = 74.24 dB, SD = 4.88). No significant effects are found for excursion size [χ^2^(2) = 1.25, *p* = 0.536], maximum *F*_0_ [χ^2^(2) = 2.44, *p* = 0.295], mean *F*_0_ [χ^2^(2) = 3.35, *p* = 0.187], minimum *F*_0_ [χ^2^(2) = 3.92, *p* = 0.141], or duration [χ^2^(2) = 1.43, *p* = 0.488].

**Table 3:** Mean post-focus acoustic measurements across focus conditions and results from the linear mixed-effects models.

Measurements	NF	IF	CF
Initial focus			
Excursion size (st.)	*M* = 3.97 (2.49)	*M* = 3.66 (2.65)	*M* = 3.58 (2.55)
χ^2^(2) = 1.25, *p* = 0.536			
Maximum *F0* (Hz)	*M* = 244.71 (40.13)	*M* = 241.75 (47.10)	*M* = 249.09 (54.63)
χ^2^(2) = 2.44, *p* = 0.295			
Mean *F0* (Hz)	*M* = 216.99 (26.64)	*M* = 214.92 (31.32)	*M* = 219.70 (35.06)
χ^2^(2) = 3.35, *p* = 0.187			
Minimum *F0* (Hz)	*M* = 194.82 (24.16)	*M* = 194.11 (26.83)	*M* = 198.00 (25.61)
χ^2^(2) = 3.92, *p* = 0.141			
Mean intensity (dB)	*M* = 77.22 (2.93)	*M* = 74.59 (4.94)	*M* = 74.24 (4.88)
χ^2^(2) = 6.31, ***p*** **= 0.043**			
Duration (ms)	*M* = 200.13 (38.78)	*M* = 195.22 (41.10)	*M* = 195.54 (40.49)
χ^2^(2) = 1.43, *p* = 0.488			
Penultimate focus			
Excursion size (st.)	*M* = 7.00 (6.30)	*M* = 7.16 (6.29)	*M* = 6.25 (5.82)
χ^2^(2) = 0.70, *p* = 0.706			
Maximum *F0* (Hz)	*M* = 233.26 (87.80)	*M* = 230.12 (79.45)	*M* = 231.42 (75.67)
χ^2^(2) = 0.30, *p* = 0.862			
Mean *F0* (Hz)	*M* = 192.32 (43.25)	*M* = 192.30 (38.11)	*M* = 196.54 (36.24)
χ^2^(2) = 1.05, *p* = 0.591			
Minimum *F0* (Hz)	*M* = 155.59 (46.50)	*M* = 152.48 (44.07)	*M* = 161.90 (44.33)
χ^2^(2) = 1.64, *p* = 0.439			
Mean intensity (dB)	*M* = 73.91 (4.14)	*M* = 72.08 (5.81)	*M* = 71.50 (5.12)
χ^2^(2) = 9.83, ***p*** **= 0.007**			
Duration (ms)	*M* = 317.25 (90.49)	*M* = 311.83 (82.20)	*M* = 309.58 (81.55)
χ^2^(2) = 0.90, *p* = 0.637			

Significant *p*-values are shown in bold. Standard deviations are given in parentheses.

A similar pattern is found in the post-focus region following sentence-penultimate focus. Focus condition significantly affects mean intensity [χ^2^(2) = 9.83, *p* = 0.007)]. Mean intensity decreases from neutral focus (*M* = 73.91 dB, SD = 4.14) to information focus (*M*=72.08 dB, SD = 5.81) and contrastive focus (*M* = 71.50 dB, SD = 5.12). No significant effects are found for excursion size [χ^2^(2) = 0.70, *p* = 0.706], maximum *F*_0_ [χ^2^(2) = 0.30, *p* = 0.862], mean *F*_0_ [χ^2^(2) = 1.05, *p* = 0.591], minimum *F*_0_ [χ^2^(2) = 1.64, *p* = 0.439], or duration [χ^2^(2) = 0.90, *p* = 0.637]. Thus, the post-focus effect is restricted to intensity, with no statistically significant evidence that focus condition affects post-focus pitch or duration.

Table 4 presents the Tukey-adjusted pairwise comparisons for mean intensity in the post-focus region. Following sentence-initial focus, mean intensity is significantly lower under information focus than under neutral focus, with an estimated difference of −2.63 dB [SE = 0.99, 95% CI [−4.90, −0.36], adjusted *p* = 0.019]. Mean intensity is also significantly lower under contrastive focus than under neutral focus, with an estimated difference of −2.97 dB [SE = 1.02, 95% CI [−5.30, −0.65], adjusted *p* = 0.008]. Information focus and contrastive focus do not differ significantly from each other [estimate = −0.34 dB, SE = 0.31, 95% CI [−1.04, 0.36], adjusted *p* = 0.478].

**Table 4:** Tukey-adjusted pairwise comparisons for significant focus effects in the post-focus regions following initial and penultimate focus.

Measurement	Contrast	Estimate	SE	95% CI	Adjusted *p*
Initial focus					
Mean intensity (dB)	IF–NF	–2.63	0.99	[–4.90, –0.36]	**0.019**
Mean intensity (dB)	CF–NF	–2.97	1.02	[–5.30, –0.65]	**0.008**
Mean intensity (dB)	CF–IF	–0.34	0.31	[–1.04, 0.36]	0.478
Penultimate focus					
Mean intensity (dB)	IF–NF	–1.82	0.79	[–3.63, –0.02]	**0.047**
Mean intensity (dB)	CF–NF	–2.41	0.69	[–3.99, –0.83]	**0.001**
Mean intensity (dB)	CF–IF	–0.58	0.36	[–1.40, 0.23]	0.211

Estimates represent the first condition minus the second condition. Confidence intervals and *p*-values are simultaneously adjusted using the single-step procedure. Only acoustic measures with a significant omnibus effect of focus are included. NF, neutral focus; IF, information focus; CF, contrastive focus. Bold values indicate statistically significant *p*-values (*p* < 0.05).

Following sentence-penultimate focus, mean intensity is likewise significantly lower under information focus than under neutral focus, with an estimated difference of −1.82 dB [SE = 0.79, 95% CI [−3.63, −0.02], adjusted *p* = 0.047]. Contrastive focus also has significantly lower post-focus intensity than neutral focus, with an estimated difference of −2.41 dB [SE = 0.69, 95% CI [−3.99, −0.83], adjusted *p* = 0.001]. The difference between contrastive focus and information focus is not significant [estimate = −0.58 dB, SE = 0.36, 95% CI [−1.40, 0.23], adjusted *p* = 0.211].

Overall, the *post-hoc* comparisons show a consistent reduction in post-focus intensity following both information focus and contrastive focus relative to neutral focus. However, the pairwise analyses did not detect a significant difference between the two focused conditions. Because none of the post-focus *F*_0_ measures shows a significant omnibus effect, these results support post-focus intensity reduction rather than post-focus *F*_0_ compression.

Table 5 presents the second post-focus analysis, which focuses on the stressed syllable of the sentence-penultimate post-focus word. Focus condition has a significant effect on excursion size [χ^2^(2) = 7.32, *p* = 0.026]. Excursion size decreases across the three focus conditions, with the largest value under neutral focus (*M* = 2.30 st, SD = 2.04), a slightly smaller value under information focus (*M* = 2.05 st, SD = 2.31), and the smallest value under contrastive focus (*M* = 1.50 st, SD = 1.38).

**Table 5:** Mean acoustic measurements on the stressed syllable of the sentence-penultimate post-focus word across focus conditions and results from the linear mixed-effects models.

Measurements	NF	IF	CF
Excursion size (st.)	*M* = 2.30 (2.04)	*M* = 2.05 (2.31)	*M* = 1.50 (1.38)
χ^2^(2) = 7.32, ***p*** **= 0.026**			
Maximum *F0* (Hz)	*M* = 234.70 (36.35)	*M* = 233.97 (44.94)	*M* = 228.90 (45.04)
χ^2^(2) = 2.79, *p* = 0.248			
Mean *F0* (Hz)	*M* = 218.95 (31.28)	*M* = 222.01 (34.57)	*M* = 218.42 (36.77)
χ^2^(2) = 2.53, *p* = 0.282			
Minimum *F0* (Hz)	*M* = 205.50 (29.49)	*M* = 207.05 (31.44)	*M* = 208.08 (27.71)
χ^2^(2) = 0.25, *p* = 0.885			
Mean intensity (dB)	*M* = 79.37 (3.28)	*M* = 76.46 (5.09)	*M* = 75.95 (5.01)
χ^2^(2) = 5.14, *p* = 0.077			
Duration (ms)	*M* = 175.95 (34.71)	*M* = 169.59 (34.65)	*M* = 169.21 (35.19)
χ^2^(2) = 1.41, *p* = 0.494			

Significant *p*-values are shown in bold. Standard deviations are given in parentheses.

Mean intensity shows a similar downward trend from neutral focus (*M* = 79.37 dB, SD = 3.28) to information focus (*M* = 76.46 dB, SD = 5.09) and contrastive focus (*M* = 75.95 dB, SD = 5.01), although the effect does not reach statistical significance [χ^2^(2) = 5.14, *p* = 0.077]. Likewise, no significant effects are found for maximum *F*_0_ [χ^2^(2) = 2.79, *p* = 0.248], mean *F*_0_ [χ^2^(2) = 2.53, *p* = 0.282], minimum *F*_0_ [χ^2^(2) = 0.25, *p* = 0.885], or duration [χ^2^(2) = 1.41, *p* = 0.494].

Table 6 presents the Tukey-adjusted pairwise comparisons for excursion size. Information focus does not differ significantly from neutral focus [estimate = −0.25 st, SE = 0.28, 95% CI [−0.90, 0.40], adjusted *p* = 0.637]. By contrast, contrastive focus shows a significantly smaller excursion size than neutral focus [estimate = −0.80 st, SE = 0.29, 95% CI [−1.48, −0.12], adjusted *p* = 0.016]. Excursion size is also significantly smaller under contrastive focus than under information focus [estimate = −0.55 st, SE = 0.20, 95% CI [−1.02, −0.09], adjusted *p* = 0.015].

**Table 6:** Tukey-adjusted pairwise comparisons for the significant focus effect on excursion size in the stressed syllable of the sentence-penultimate post-focus word.

Measurement	Contrast	Estimate	SE	95% CI	Adjusted *p*
Excursion size (st.)	IF–NF	–0.25	0.28	[–0.90, 0.40]	0.637
Excursion size (st.)	CF–NF	–0.80	0.29	[–1.48, –0.12]	**0.016**
Excursion size (st.)	CF–IF	–0.55	0.20	[–1.02, –0.09]	**0.015**

Estimates represent the first condition minus the second condition. Confidence intervals and *p*-values are simultaneously adjusted using the single-step procedure. NF, neutral focus; IF, information focus; CF, contrastive focus. Bold values indicate statistically significant *p*-values (*p* < 0.05).

These comparisons indicate that the significant omnibus effect is driven primarily by the reduced excursion size under contrastive focus. Information focus does not differ significantly from the neutral-focus baseline, whereas contrastive focus exhibits a localized reduction in pitch excursion at the sentence-penultimate post-focus word.

The post-focus analyses provide strong evidence for post-focus intensity attenuation across the broader post-focus domain but only limited evidence for localized modification of *F*_0_-related measures. When all post-focus content words are considered, mean intensity is significantly reduced following both information focus and contrastive focus, whereas none of the *F*_0_-related measures differs significantly across focus conditions. In the second analysis, which focuses exclusively on the stressed syllable of the sentence-penultimate post-focus word, excursion size shows a significant effect of focus. The *post-hoc* comparisons indicate that this effect is driven by a reduced excursion size under contrastive focus relative to both neutral focus and information focus, while information focus does not differ significantly from the neutral baseline. Thus, the localized analysis provides evidence for a contrastive-focus-specific reduction in pitch excursion rather than a general post-focus pitch-range compression following narrow focus. Overall, the results suggest that post-focus realization in Qassimi Arabic is characterized primarily by intensity attenuation, with only limited and position-specific modification of pitch excursion.

##### 2.2.2.3 Pre-focus region

Table 7 presents the descriptive statistics and linear mixed-effects model results for the pre-focus region. In the pre-focus region preceding sentence-penultimate focus, focus condition has a significant effect only on mean intensity [χ^2^(2) = 10.73, *p* = 0.005]. Mean intensity is highest under neutral focus (*M* = 82.00 dB, SD = 1.94), followed by information focus (*M* = 81.00 dB, SD = 2.97), and is lowest under contrastive focus (*M* = 80.48 dB, SD = 2.94).

**Table 7:** Mean pre-focus acoustic measurements across focus conditions and results from the linear mixed-effects models.

Measurements	NF	IF	CF
Penultimate focus			
Excursion size (st.)	*M* = 3.38 (1.73)	*M* = 2.94 (1.23)	*M* = 2.95 (1.33)
χ^2^(2) = 2.00, *p* = 0.367			
Maximum *F0* (Hz)	*M* = 257.91 (39.51)	*M* = 262.46 (32.91)	*M* = 261.00 (35.10)
χ^2^(2) = 0.92, *p* = 0.631			
Mean *F0* (Hz)	*M* = 237.33 (32.07)	*M* = 242.90 (28.01)	*M* = 241.12 (28.05)
χ^2^(2) = 1.37, *p* = 0.503			
Minimum *F0* (Hz)	*M* = 211.78 (24.72)	*M* = 220.67 (21.70)	*M* = 218.94 (20.62)
χ^2^(2) = 4.57, *p* = 0.102			
Mean intensity (dB)	*M* = 82.00 (1.94)	*M* = 81.00 (2.97)	*M* = 80.48 (2.94)
χ^2^(2) = 10.73, ***p*** **= 0.005**			
Duration (ms)	*M* = 188.79 (31.91)	*M* = 180.36 (34.31)	*M* = 182.32 (38.46)
χ^2^(2) = 3.14, *p* = 0.208			
Final focus			
Excursion size (st.)	*M* = 2.99 (1.85)	*M* = 3.02 (1.73)	*M* = 3.06 (1.70)
χ^2^(2) = 0.19, *p* = 0.910			
Maximum *F0* (Hz)	*M* = 255.08 (47.36)	*M* = 264.22 (48.02)	*M* = 264.98 (46.06)
χ^2^(2) = 2.28, *p* = 0.319			
Mean *F0* (Hz)	*M* = 235.64 (33.14)	*M* = 243.59 (34.20)	*M* = 244.17 (32.99)
χ^2^(2) = 2.32, *p* = 0.314			
Minimum *F0* (Hz)	*M* = 212.46 (23.26)	*M* = 219.34 (25.37)	*M* = 219.75 (24.28)
χ^2^(2) = 2.86, *p* = 0.239			
Mean intensity (dB)	*M* = 81.85 (2.16)	*M* = 80.95 (3.05)	*M* = 80.35 (3.82)
χ^2^(2) = 3.49, *p* = 0.175			
Duration (ms)	*M* = 186.56 (31.76)	*M* = 183.14 (32.41)	*M* = 185.58 (36.20)
χ^2^(2) = 1.00, *p* = 0.606			

Significant *p*-values are shown in bold. Standard deviations are given in parentheses.

No significant effects are found for excursion size [χ^2^(2) = 2.00, *p* = 0.367], maximum *F*_0_ [χ^2^(2) = 0.92, *p* = 0.631], mean *F*_0_ [χ^2^(2) = 1.37, *p* = 0.503], minimum *F*_0_ [χ^2^(2) = 4.57, *p* = 0.102], or duration [χ^2^(2) = 3.14, *p* = 0.208]. In the pre-focus region preceding sentence-final focus, focus condition does not have a significant effect on any acoustic measure: excursion size [χ^2^(2) = 0.19, *p* = 0.910], maximum *F*_0_ [χ^2^(2) = 2.28, *p* = 0.319], mean *F*_0_ [χ^2^(2) = 2.32, *p* = 0.314], minimum *F*_0_ [χ^2^(2) = 2.86, *p* = 0.239], mean intensity [χ^2^(2) = 3.49, *p* = 0.175], or duration [χ^2^(2) = 1.00, *p* = 0.606].

Table 8 presents the Tukey-adjusted pairwise comparisons for mean intensity in the pre-focus region preceding sentence-penultimate focus. Mean intensity is significantly lower under contrastive focus than under neutral focus [estimate = −1.52 dB, SE = 0.45, 95% CI [−2.56, −0.49], adjusted *p* = 0.002]. By contrast, information focus does not differ significantly from neutral focus [estimate = −1.00 dB, SE = 0.60, 95% CI [−2.37, 0.37], adjusted *p* = 0.193], and contrastive focus does not differ significantly from information focus [estimate = −0.52 dB, SE = 0.29, 95% CI [−1.20, 0.15], adjusted *p* = 0.156]. The significant omnibus effect is therefore driven primarily by the lower pre-focus intensity under contrastive focus relative to the neutral-focus condition.

**Table 8:** Tukey-adjusted pairwise comparisons for the significant focus effect on mean intensity in the pre-focus region preceding sentence-penultimate focus.

Measurement	Contrast	Estimate	SE	95% CI	Adjusted *p*
Mean intensity (dB)	IF–NF	–1.00	0.60	[–2.37, 0.37]	0.193
Mean intensity (dB)	CF–NF	–1.52	0.45	[–2.56, –0.49]	**0.002**
Mean intensity (dB)	CF–IF	–0.52	0.29	[–1.20, 0.15]	0.156

Estimates represent the first condition minus the second condition. Confidence intervals and *p*-values are simultaneously adjusted using the single-step procedure. NF, neutral focus; IF, information focus; CF, contrastive focus. Bold values indicate statistically significant *p*-values (*p* < 0.05).

### 2.3 Discussion

The primary aim of the quantitative analyses was to determine how focus is prosodically realized in Qassimi Arabic and whether information focus and contrastive focus differ acoustically. The results show that focus is encoded primarily through systematic manipulation of *F*_0_-related parameters. In the on-focus region, focused constituents generally exhibit higher pitch values than their neutral-focus counterparts, with pitch-excursion expansion occurring most clearly in the sentence-initial position. Maximum *F*_0_, mean *F*_0_, minimum *F*_0_, and excursion size emerge as the principal acoustic correlates of focus, whereas duration and intensity play more limited roles. These findings align Qassimi Arabic with numerous Arabic dialects and other languages in which focus is realized primarily through pitch-range expansion and pitch-register raising rather than through extensive temporal restructuring (Xu, 1999; Xu and Wang, 2001; Chen et al., 2009; Alzaidi et al., 2019, 2023, 2025).

The analyses further reveal clear positional asymmetries in focus realization. Sentence-penultimate focus displays the most extensive enhancement, with significant increases in maximum, mean, and minimum *F*_0_. Sentence-initial focus is characterized by significant increases in excursion size and maximum *F*_0_, whereas sentence-final focus shows a more restricted pattern involving significant increases in mean and minimum *F*_0_. This pattern suggests that utterance-final tonal constraints limit the realization of large pitch excursions and high pitch peaks while still allowing focused constituents to maintain prominence through an elevated pitch register. Such positional effects are consistent with previous findings from Hijazi, Makkan, Emirati, and Iraqi Arabic, where sentence position has likewise been shown to influence the phonetic realization of focus (Alzaidi et al., 2019; Alzaidi, 2022; Alzaidi et al., 2023, 2025).

A central motivation of the present study was to determine whether Qassimi Arabic exhibits post-focus compression (PFC), given the mixed findings reported across Arabic dialects. The results provide robust evidence of post-focus intensity attenuation but more restricted evidence of post-focus pitch-range modification. Across the broader post-focus domain, focus condition significantly affects mean intensity following both sentence-initial and sentence-penultimate focus. In both positions, post-focus intensity is significantly lower under information focus and contrastive focus than under neutral focus, while the two narrow-focus conditions do not differ significantly from each other. By contrast, *F*_0_ excursion size and maximum, mean, and minimum *F*_0_ show no significant broad effects of focus condition.

The localized analysis of the stressed syllable of the sentence-penultimate post-focus word reveals a more specific pattern. Excursion size differs significantly across focus conditions and is significantly smaller following contrastive focus than following both neutral focus and information focus. Information focus, however, does not differ significantly from neutral focus. This result therefore provides evidence of localized pitch-range reduction following contrastive focus, but not of a general compression pattern shared by both narrow-focus conditions.

These findings must also be distinguished from post-focus register lowering. Qassimi Arabic shows no significant broad reduction in post-focus maximum, mean, or minimum *F*_0_. Although compression of excursion size need not necessarily occur at a lowered pitch register, the present evidence is confined to one measure, one localized post-focus position, and one focus type. The results therefore do not demonstrate a broad or uniform lowering or compression of the post-focus *F*_0_ contour.

Accordingly, the present findings do not justify an unqualified classification of Qassimi Arabic as a +PFC variety. Instead, they reveal cue-specific, focus-type-specific, and position-specific post-focus modification. Post-focus intensity attenuation is consistent across information focus and contrastive focus in the broader post-focus domain, whereas localized pitch-excursion reduction occurs only following contrastive focus. Compared with the clearer post-focus *F*_0_ compression reported for Hijazi, Emirati, and Iraqi Arabic (Alzaidi et al., 2019, 2023, 2025), the Qassimi Arabic pattern is more restricted, while differing from Makkan Arabic, where post-focus compression appears largely absent (Alzaidi, 2022).

The pre-focus analyses reveal little evidence for systematic anticipatory modification before the focused constituent. No acoustic measure differs significantly across focus conditions before sentence-final focus. Before sentence-penultimate focus, the only significant effect involves mean intensity, which is significantly lower under contrastive focus than under neutral focus. Information focus does not differ significantly from either neutral focus or contrastive focus. Thus, the pre-focus findings provide evidence of a limited, contrastive-focus-specific intensity reduction rather than broad anticipatory restructuring across *F*_0_, duration, and intensity.

With regard to the distinction between information focus and contrastive focus, most direct comparisons between the two conditions do not reach statistical significance. This absence of significance is not interpreted as evidence that the two focus types are acoustically equivalent. Rather, under the present experimental conditions, the analyses did not detect a consistent acoustic distinction between them across measures and sentence positions. Two reliable, measure-specific differences were nevertheless observed: contrastive focus had a significantly higher maximum *F*_0_ than information focus in the sentence-penultimate on-focus position and a significantly smaller excursion size than information focus in the localized sentence-penultimate post-focus word. The findings therefore provide evidence of isolated acoustic differentiation but not of a consistent distinction across the acoustic parameters and positions examined. Given that the study involved ten female speakers from Buraidah and used a controlled question–answer elicitation paradigm, these results should be interpreted as characterizing the present sample and experimental conditions rather than establishing the absence of an information-focus–contrastive-focus distinction in Qassimi Arabic generally. Further research with larger and more diverse speaker samples and additional communicative contexts is required to determine the broader generality of this pattern.

## 3 Computational modeling

The preceding acoustic analyses identified the major acoustic cues to focus in Qassimi Arabic. However, they do not reveal how different prosodic functions interact to generate the full intonational contours or their relative contribution to the formation of the contours. The second part of this study is to examine how well *F*_0_ contours of Qassimi Arabic can be reproduced by a computational model based on the PENTA framework, and what are the likely communicative functions that contribute to the shaping of the *F*_0_ contours in the dialect.

### 3.1 PENTAtrainer—The modeling tool

Computational modeling is conducted using a new version of PENTAtrainer (Prom-on et al., 2012; Xu and Prom-On, 2014), a modeling tool based on the PENTA framework described in Section 1.2. PENTAtrainer is a Praat script that incorporates a learning component written in Java (original version) or Python (the new version). The workflow consisted of three stages: functional annotation, parameter learning, and *F*_0_ contour synthesis.

Following the standard PENTAtrainer architecture, the speech corpus was represented through layered functional annotation, pseudo-hierarchical representation, and syllable-edge synchronization (Xu and Prom-On, 2014). Five functional layers were included in the present study: Stress (stressed vs. unstressed syllables), Weight (light, heavy, and superheavy syllables), Syllable Position (initial, penultimate, and final), Syllable Position within the Prosodic Word (SPword: prosodic-word-initial, prosodic-word-medial, and prosodic-word-final), and Focus (pre-focus, on-focus, and post-focus). These layers were synchronized at syllable boundaries, allowing each syllable to be represented by a unique combination of functional categories.

For each unique combination of functional categories across the five annotation layers, PENTAtrainer estimated three qTA (stands for the quantitative Target Approximation model) parameters: target slope (*m*), target height (*b*), and target-approximation strength (λ) (Prom-On et al., 2009). Parameter optimization was performed using simulated annealing (Kirkpatrick et al., 1983; Xu and Prom-On, 2014), whereby parameter values were iteratively adjusted to minimize the discrepancy between observed and synthesized *F*_0_ contours.

Following parameter estimation, a synthesis module reconstructed complete *F*_0_ contours for each utterance from the learned pitch-target parameters. Model performance was evaluated by comparing synthesized and observed contours using root mean square error (RMSE) and Pearson's correlation coefficient (*r*). In addition to evaluating the full model, a series of function-removal analyses were conducted in which individual functional layers were excluded from the model. Changes in prediction accuracy resulting from the removal of a given function were used to assess its relative contribution to contour generation.

### 3.2 Functional annotation, training, and cross-validation design

The 12 base sentences (Section 2.1.1) were organized into 12 sentence groups, each consisting of the same lexical sentence produced under the three focus conditions (neutral, information, and contrastive focus). To evaluate the generalizability of the learned functional representations, the corpus was partitioned by sentence group. All realizations of a given sentence, including all speakers, repetitions, and focus conditions, were assigned to the same partition. This procedure prevented lexical overlap between training and testing data and ensured that model evaluation was conducted entirely on unseen sentence forms.

Model evaluation employed leave-one-sentence-group-out cross-validation across the 12 sentence groups, yielding 12 independent folds. In each fold, one sentence group served as the testing set (150 utterances), while the remaining 11 sentence groups constituted the training set (1,650 utterances). All utterances were annotated using the five functional tiers summarized in Table 9. Annotation was carried out independently for each tier following the procedures described in Sections 1.2 and 3.1. Figure 6 illustrates an example of the resulting multi-tier annotation structure.

**Table 9:** Functional annotation tiers and labels used for PENTAtrainer modeling.

Functional tier	Label set	Interpretation
Stress	S, U	Lexical stress categories distinguishing stressed syllables (S) from unstressed syllables (U).
Syllable Weight	R, H, L	Prosodic weight categories based on syllable structure: R (= superheavy) = CVVC/CVCC; H (= heavy) = CVV/CVC; L (= light) = CV.
Sposition	I, P, F	Position of the stressed syllable within the lexical word: word-initial (I), word-penultimate (P), and word-final (F).
SPword	SI, SM, SF	Position of the syllable within the prosodic word, where SI, SM, and SF denote prosodic-word-initial, prosodic-word-medial, and prosodic-word-final syllable positions, respectively.
Focus	PRE, ON, POS	Discourse-related focus regions corresponding to material preceding the focused constituent (PRE), the focused constituent itself (ON), and post-focus material following the focused constituent (POS).

**Figure 6:**
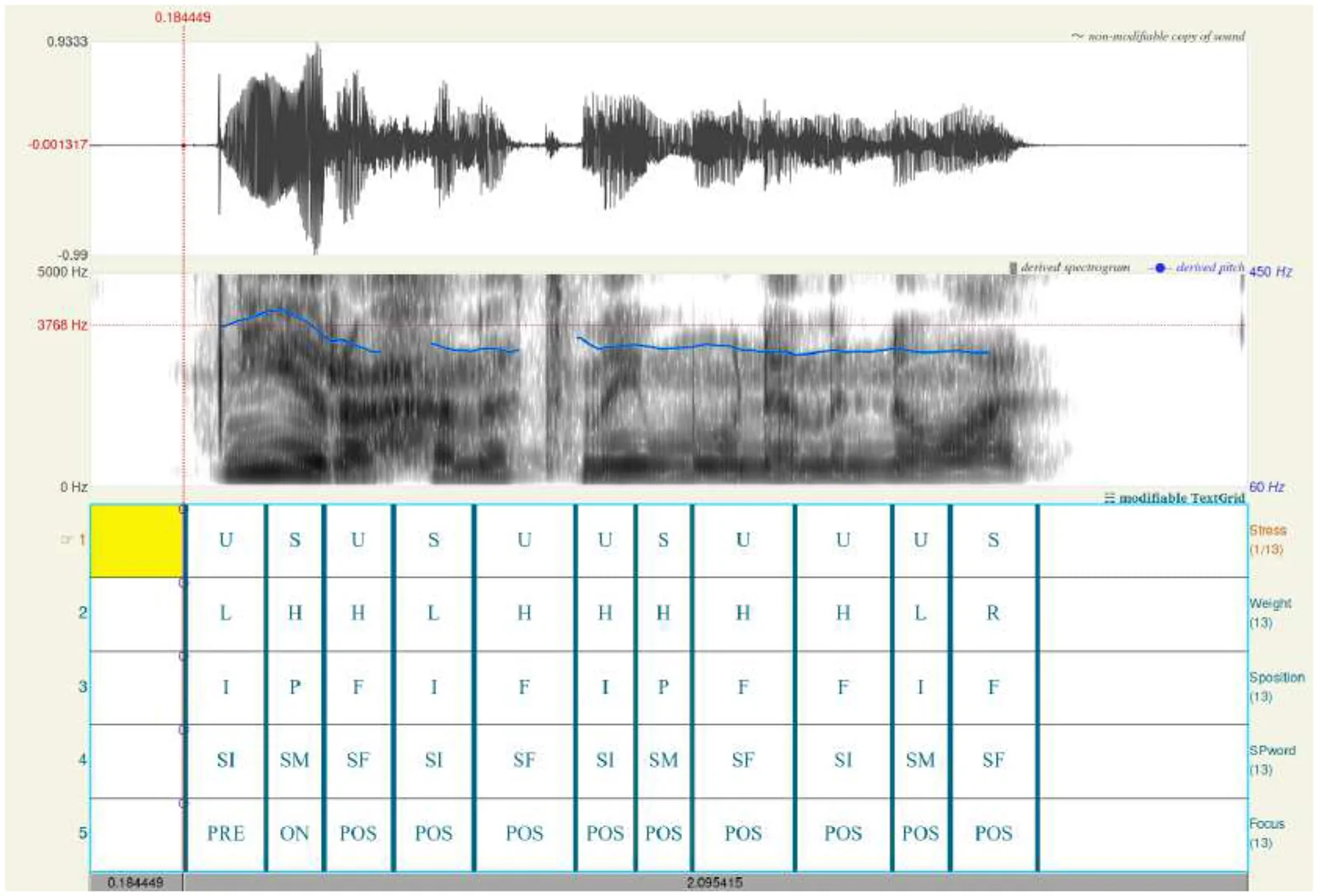
Example of the multi-tier functional annotation structure used for PENTAtrainer modeling. The waveform, spectrogram, and *F*_0_ contour are aligned with five independently annotated functional tiers: Stress, Weight, Sposition, SPword, and Focus. Each syllable is simultaneously associated with multiple communicative-functional categories, allowing the model to estimate shared *F*_0_ target parameters across interacting prosodic functions.

In the focus tier, PRE denotes the unmarked, default pitch-range state in which no local focus-driven adjustment—expansion or compression—occurs, in contrast to ON (expanded, focused) and POS (compressed, post-focal). Because a neutral-focus sentence contains no ON or POS region by definition, every syllable in it defaults to PRE, which represents the absence of focus-driven modifications. This approach is shown to be effective in previous modeling works (Prom-On et al., 2009; Xu and Prom-On, 2014; Chen and Xu, 2025).

Model training was performed separately for each cross-validation fold using the PENTAtrainer learning algorithm. All five functional tiers were included simultaneously during parameter estimation. Learning was conducted for 750 iterations, consisting of 450 iterations of global optimization followed by 300 iterations of fine-tuning. The same optimization settings were used across all folds to ensure comparability of parameter estimation. After training, the learned parameters were applied to the held-out sentence group to synthesize *F*_0_ contours for utterances not encountered during learning. Model performance was evaluated using root mean square error (RMSE) and Pearson's correlation coefficient (*r*). In addition to the full model, function-removal analyses were conducted to assess the relative contribution of individual functional layers to contour generation. Performance measures were computed separately for each fold and subsequently aggregated across folds.

### 3.3 Results

#### 3.3.1 Visual comparison of original and synthesized *F*_0_ contours

Figures 7–9 present mean contour comparisons for three utterances in which focus occurs in different sentence positions: sentence-initial focus in /*Dī.ma ḥa.mat May.mū.nah min Na.wāl*/, sentence-penultimate focus in /*May.mū.nah ja.bat Mi.nī.rah li Na.wāl*/, and sentence-final focus in /*May.mū.nah wad.dat Na.wāl li Lī.na*/. The original contours represent mean *F*_0_ values averaged across 50 utterances for each target sentence, while the synthesized contours were generated by the full-function PENTAtrainer model using the parameters learned from the corpus (Table 10). The examples are shown to facilitate qualitative inspection of contour shape and model deviations.

**Figure 7:**
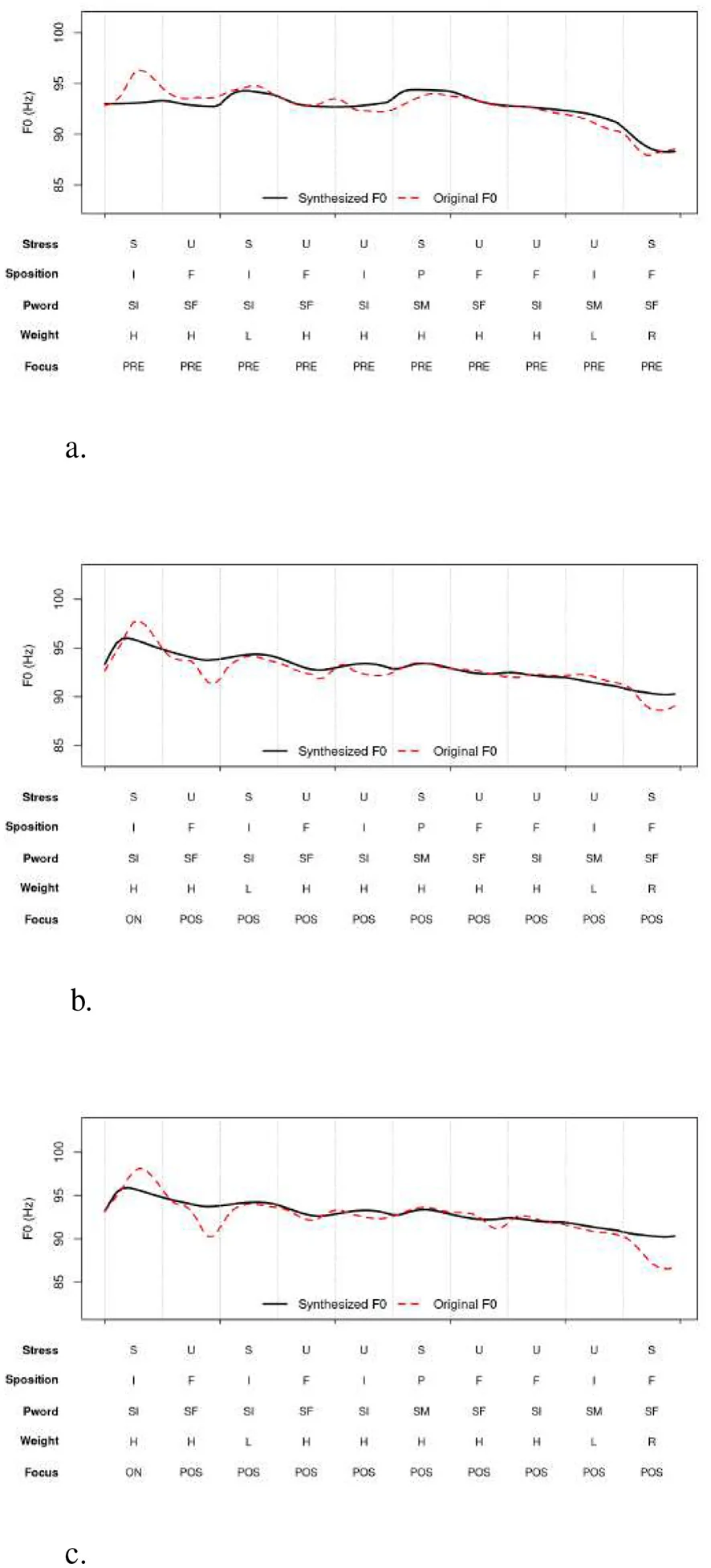
Observed and PENTAtrainer-synthesized mean *F*_0_ contours for a representative sentence with the target word in sentence-initial position. The panels show neutral focus (NF), information focus (IF), and contrastive focus (CF). The original contour is represented by the red dashed line, and the synthesized contour is represented by the black solid line. Each observed contour is averaged across 50 utterances, comprising ten speakers and five repetitions per speaker. The synthesized contour was generated from the parameters learned by the full PENTAtrainer model. Vertical dashed lines indicate interval boundaries, and the tiers below each contour show the functional annotations used in synthesis: Stress, SPosition, SPword, Weight, and Focus. **(a)** NF: /Dī.ma ḥa.mat May.mū.nah min Na.wāl/. **(b)** IF: /Dī.ma ḥa.mat May.mū.nah min Na.wāl/. **(c)** CF: /Dī.ma ḥa.mat May.mū.nah min Na.wāl/.

**Figure 8:**
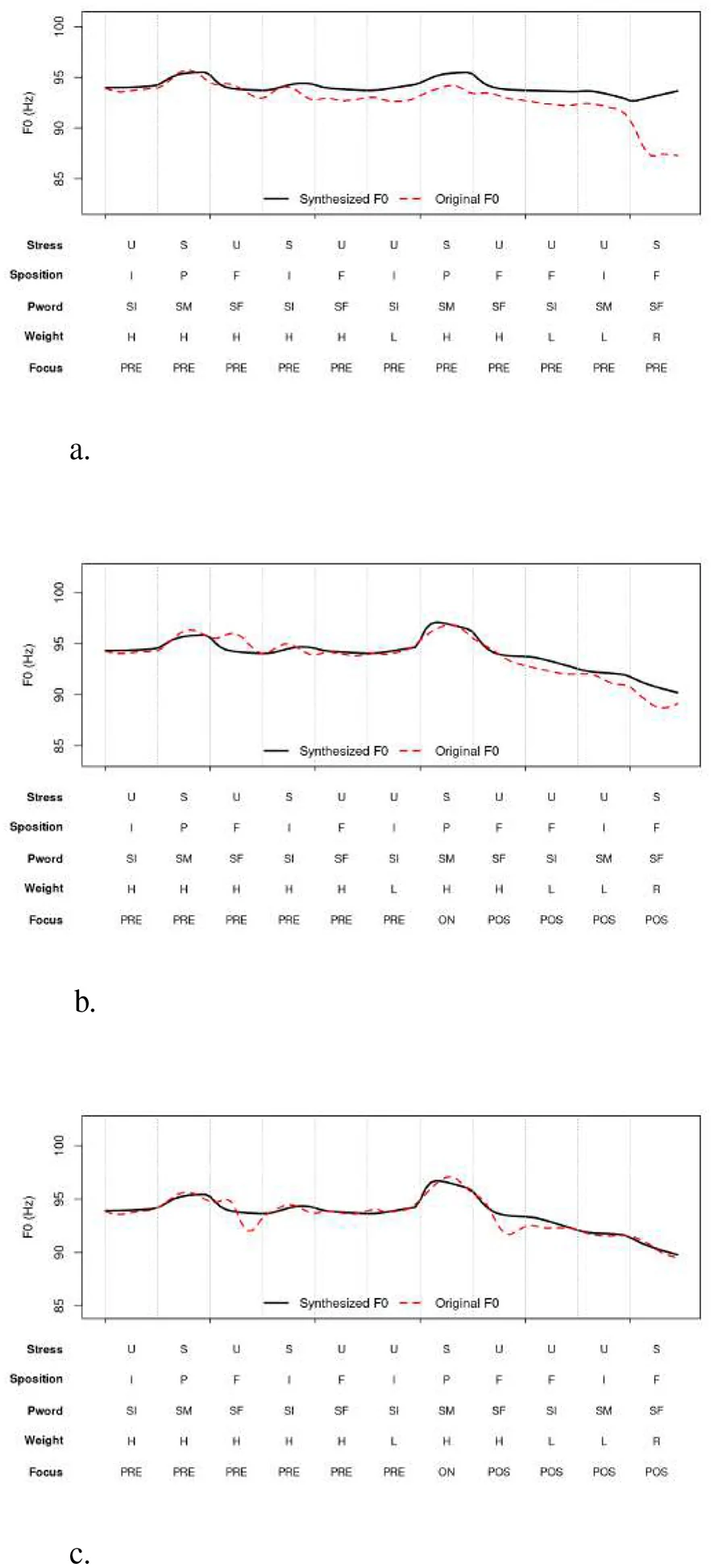
Observed and PENTAtrainer-synthesized mean *F*_0_ contours for a representative sentence with the target word in sentence-penultimate position. The panels show neutral focus (NF), information focus (IF), and contrastive focus (CF). The original contour is represented by the red dashed line, and the synthesized contour is represented by the black solid line. Each observed contour is averaged across 50 utterances, comprising ten speakers and five repetitions per speaker. The synthesized contour was generated from the parameters learned by the full PENTAtrainer model. Vertical dashed lines indicate interval boundaries, and the tiers below each contour show the functional annotations used in synthesis: Stress, SPosition, SPword, Weight, and Focus. **(a)** NF: /May.mū.nah ja.bat Mi.nī.rah li Na.wāl/. **(b)** IF: /May.mū.nah ja.bat Mi.nī.rah li Na.wāl/. **(c)** CF: /May.mū.nah ja.bat Mi.nī.rah li Na.wāl/.

**Figure 9:**
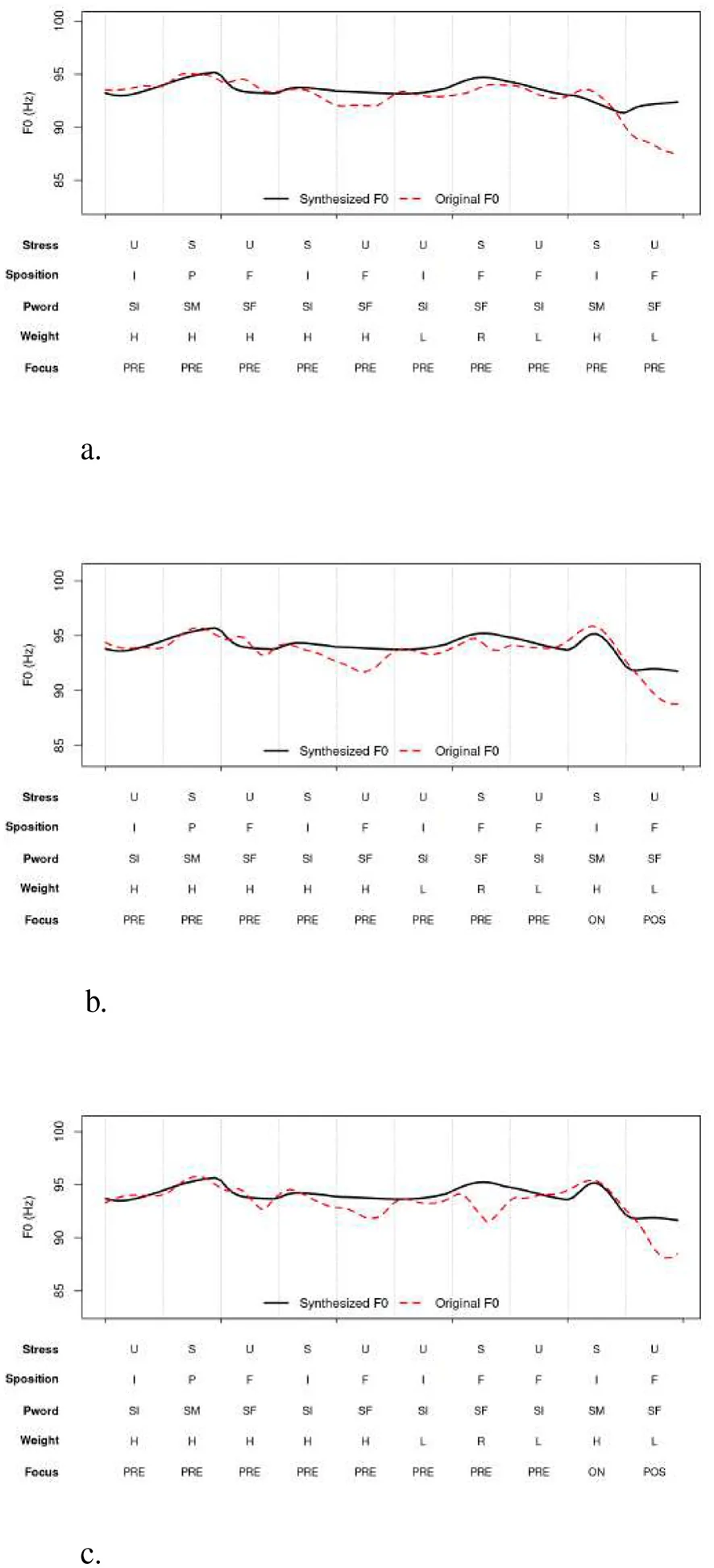
Observed and PENTAtrainer-synthesized mean *F*_0_ contours for a representative sentence with the target word in sentence-final position. The panels show neutral focus (NF), information focus (IF), and contrastive focus (CF). The original contour is represented by the red dashed line, and the synthesized contour is represented by the black solid line. Each observed contour is averaged across 50 utterances, comprising ten speakers and five repetitions per speaker. The synthesized contour was generated from the parameters learned by the full PENTAtrainer model. Vertical dashed lines indicate interval boundaries, and the tiers below each contour show the functional annotations used in synthesis: Stress, SPosition, SPword, Weight, and Focus. **(a)** NF: /May.mū.nah wad.dat Na.wāl li Lī.na/. **(b)** IF: /May.mū.nah wad.dat Na.wāl li Lī.na/. **(c)** CF: /May.mū.nah wad.dat Na.wāl li Lī.na/.

**Table 10:** Means and standard deviations of the PENTAtrainer parameter values across the twelve cross-validation folds.

Stress	Weight	Sposition	SPword	Focus	$\bar{m}$	*SD*(*m*)	$\bar{b}$	*SD*(*b*)	$\bar{l}$	*SD*(*l*)
S	H	I	SI	ON	–4.88	15.19	3.51	0.83	53.71	28.31
S	H	I	SM	ON	–23.41	10.87	–0.98	2.87	54.38	25.31
S	H	P	SM	ON	–37.17	23.19	3.88	2.68	43.33	29.88
U	H	F	SF	POS	1.10	13.99	–0.80	1.76	50.56	29.62
U	H	F	SI	POS	–7.36	41.22	–2.92	13.88	43.06	33.84
U	L	F	SF	POS	–2.81	20.09	–3.61	6.84	46.21	28.38
U	L	F	SI	POS	–13.61	47.75	–0.23	11.58	31.66	18.12
S	R	F	SF	POS	8.41	27.56	–3.90	8.36	43.47	35.10
U	H	I	SI	POS	5.20	37.35	5.43	11.16	31.31	29.73
S	H	P	SM	POS	–19.58	21.65	0.48	7.93	42.98	37.91
U	L	I	SM	POS	–20.12	30.58	1.55	6.29	52.49	36.26
S	L	I	SI	POS	–9.94	15.96	1.97	2.13	47.35	25.15
U	H	F	SF	PRE	–1.83	4.36	1.80	7.15	53.36	29.29
U	H	F	SI	PRE	–17.95	44.43	0.71	12.94	24.46	33.69
U	L	F	SF	PRE	14.65	23.63	–4.20	10.42	22.21	32.56
U	L	F	SI	PRE	–6.23	20.37	–0.06	6.28	23.91	23.05
S	R	F	SF	PRE	–1.91	16.19	0.52	5.89	21.92	26.83
U	H	I	SI	PRE	22.41	36.60	–2.82	14.86	25.92	27.25
S	H	I	SI	PRE	–0.11	23.84	0.61	5.14	47.82	33.57
S	H	I	SM	PRE	–12.38	50.00	1.32	12.74	35.95	39.29
S	H	P	SM	PRE	0.54	23.91	–4.40	12.59	29.05	29.97
U	L	I	SI	PRE	1.84	15.95	3.10	4.81	38.37	32.37
U	L	I	SM	PRE	–21.30	14.63	–0.65	1.79	51.98	32.74
S	L	I	SI	PRE	–17.17	36.24	0.42	4.24	48.74	33.55

*m* (st/s) is target slope; *b* is target height (st); and *l* is target strength (no unit). The values of these parameters reflect joint contributions of stress, syllable weight, stress position, syllable position within the prosodic word (SPword), and focus region. S and U denote stressed and unstressed syllables; L, H, and R denote light, heavy, and superheavy syllables; I, P, and F denote word-initial, word-penultimate, and word-final stress positions; SI, SM, and SF denote prosodic-word-initial, prosodic-word-medial, and prosodic-word-final syllable positions, respectively; and ON, PRE, and POS denote on-focus, pre-focus, and post-focus regions, respectively.

The figures reveal a close correspondence between the synthesized and original *F*_0_ contours across all focus conditions. In each panel, the synthesized contour reproduces the overall trajectory of the original contour, including the major pitch prominences associated with stressed syllables. In neutral focus (Figures 7a, 8a, 9a), the two contours follow a highly similar pattern throughout the utterance. In the focus conditions, the synthesized contours capture the localized *F*_0_ rise on the focused syllable, the relatively stable pitch patterns in the pre-focus regions, and the broad contour patterns in the post-focus regions. Overall, the figures show that the full-function PENTAtrainer model successfully reproduces the principal intonational patterns.

Table 10 shows the mean parameter values learned by PENTAtrainer across the twelve cross-validation folds together with their standard deviations. The final inventory consists of 24 parameter combinations distributed across the three focus regions. The standard deviations indicate that parameter stability varied across combinations and parameters, with some estimates showing greater cross-fold variability than others.

The on-focus region is represented by only three parameter combinations, all corresponding to stressed heavy syllables. Two combinations involve word-initial stress and one involves word-penultimate stress. This restricted inventory reflects the phonological characteristics of the focused words in the corpus, whose focus-bearing syllables were consistently stressed and heavy. Across all three combinations, the learned target slopes (*m*) are negative, target-height values (*b*) are relatively high (greater than or close to 0 st), and target-strength values (*l*) are relatively large. The standard deviations of *m* and *b* are relatively small compared with those in focus regions, suggesting relatively consistent target specifications in the on-focus region.

In contrast, the post-focus region contains nine parameter combinations and exhibits substantially greater structural diversity. Both stressed and unstressed syllables are represented, together with light, heavy, and superheavy syllable types. The learned target slopes range from negative to positive values, while target height (*b*) and target strength (*l*) also vary across parameter combinations. This variability indicates that the model learned distinct target specifications for different post-focus syllable categories rather than a single uniform representation. The broader inventory is consistent with the greater phonological diversity of syllables occurring after the focused constituent.

The pre-focus region constitutes the largest component of the learned inventory, with twelve parameter combinations. As in the post-focus region, both stressed and unstressed syllables are represented, together with all three weight categories. Considerable variation is observed in the learned values of target slope, target height, and target strength, indicating substantial differentiation among pre-focus syllable categories. The larger number of parameter combinations reflects the wider range of phonological and positional contexts that occur before the focused constituent. Taken together, the results show that the learned parameter inventory is highly constrained in the on-focus region but considerably more diverse in the pre-focus and post-focus regions, reflecting the distribution of prosodic contexts in the corpus.

#### 3.3.2 Functional-layer ablation analysis

Table 11 summarizes the performance of the full-function model and the ablation models using RMSE and Pearson's correlation coefficient (*r*), together with the corresponding changes relative to the full-function model. Lower RMSE values and higher correlation coefficients indicate closer agreement between the synthesized and original *F*_0_ contours. The full-function model achieved a mean RMSE of 2.9114 st and a mean correlation of 0.4279. The clearest deterioration occurred when both Focus and SPword were removed: this condition produced the only increase in RMSE relative to the full model (ΔRMSE = +0.0569) together with the largest reduction in correlation (Δ r = –0.1150). By contrast, removing Weight or Stress slightly improved both evaluation measures relative to the full model. The No Weight condition produced the lowest RMSE (2.8023 st) and highest correlation (0.4432), while the No Stress condition also yielded a lower RMSE (2.8067 st) and a slightly higher correlation (0.4322). Several other ablation conditions produced mixed effects, typically involving a modest reduction in RMSE accompanied by a reduction in correlation. The results therefore do not support a single ordinal hierarchy of positive functional contribution; instead, the effects of removing individual functional tiers are interpreted relative to the full-function model and across both evaluation metrics.

**Table 11:** Performance of the full-function and ablation models.

Condition	Mean RMSE	SD RMSE	Median RMSE	ΔRMSE	Mean *r*	SD *r*	Median *r*	*Δr*
Full-function model	2.9114	1.8119	2.3515	–	0.4279	0.3077	0.4735	–
No stress	2.8067	1.7180	2.2925	–0.1047	0.4322	0.3099	0.4863	+0.0043
No weight	2.8023	1.7475	2.2711	–0.1091	0.4432	0.2894	0.4922	+0.0153
No Sposition	2.8502	1.7444	2.3310	-0.0612	0.4082	0.3041	0.4597	-0.0197
No SPword	2.8566	1.7705	2.3293	–0.0548	0.3885	0.3057	0.4200	–0.0394
No focus	2.8428	1.7543	2.3221	–0.0686	0.4150	0.3117	0.4724	–0.0129
No stress + Sposition	2.8705	1.7520	2.3514	–0.0409	0.3830	0.3015	0.4353	–0.0449
No stress + weight	2.8330	1.7542	2.3004	–0.0784	0.4273	0.2809	0.4711	–0.0006
No weight + Sposition	2.8281	1.7288	2.2837	–0.0833	0.4165	0.2980	0.4466	–0.0114
No focus + SPword	2.9683	1.8035	2.4090	+0.0569	0.3129	0.2988	0.3423	–0.1150
No focus + stress	2.8687	1.7507	2.3334	–0.0427	0.3896	0.3121	0.4427	–0.0383

RMSE values are in semitones (st). ΔRMSE and Δ*r* indicate changes relative to the full-function model. Negative ΔRMSE indicates lower error, whereas positive Δ*r* indicates higher correlation. Because the two performance measures do not always change in the same direction, the ablation conditions are not interpreted as forming a single ordinal hierarchy of functional contribution.

Figures 10–17 present mean contour comparisons for the same three utterances, but with one or two functions removed from the PENTAtrainer model. The utterances represent sentence-initial, sentence-penultimate, and sentence-final focus. The original contours are averaged across 50 utterances for each target sentence, while the synthesized contours show the model output after excluding the relevant function or function pair. These comparisons illustrate how removal of particular functional layers affects reconstruction of the *F*_0_ contour.

**Figure 10:**
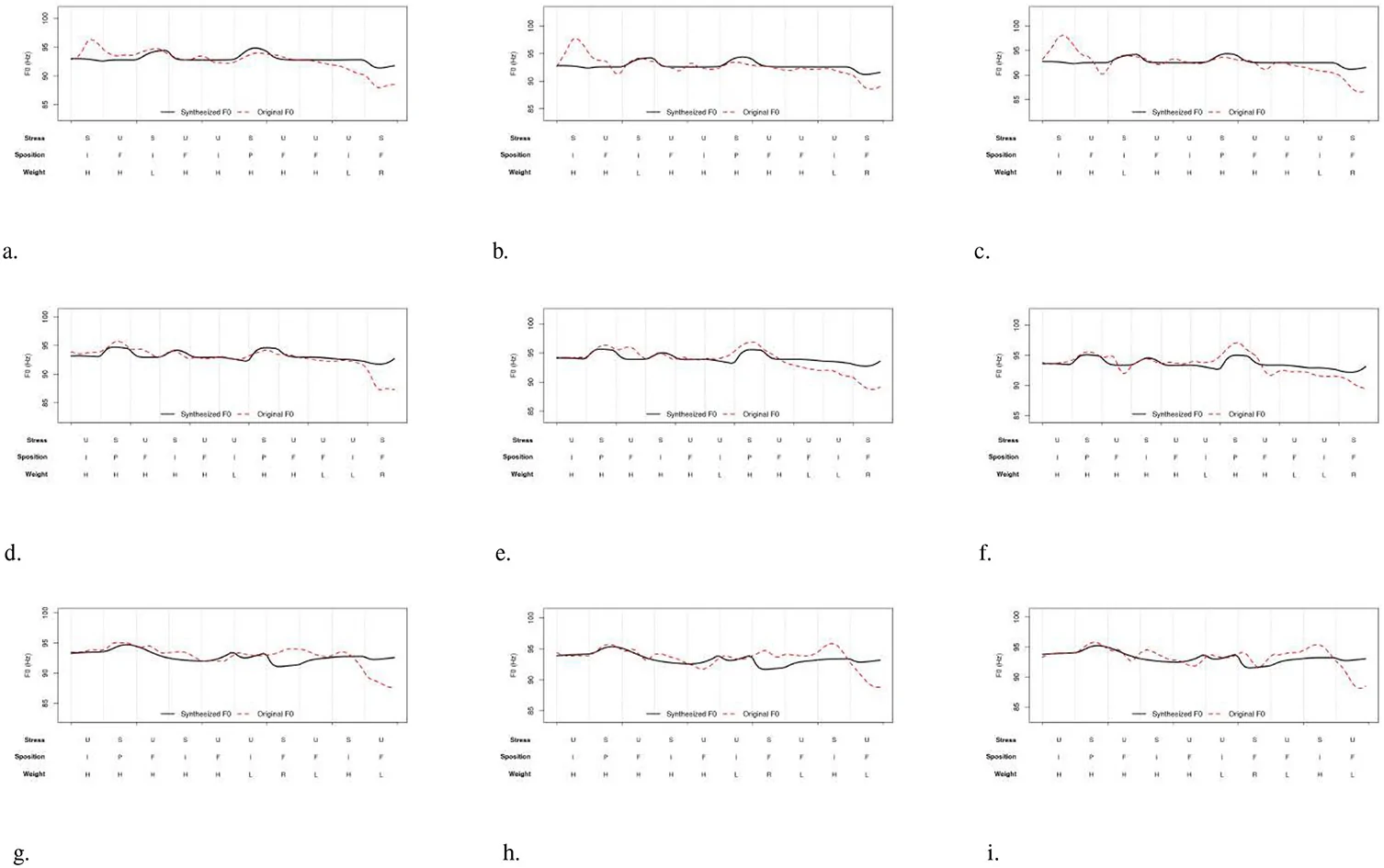
Mean *F*_0_ contours for the original (red dashed line) and synthesis (black solid line) are shown for **(a)** NF, **(b)** IF, and **(c)** CF. In each panel, the contour is averaged across 50 utterances, representing 10 speakers with five repetitions each for the relevant target sentence. The synthesized contour was produced from the learned PENTAtrainer parameters. The vertical dashed lines mark interval boundaries, and the tiers below the contours display the functional annotations used in synthesis: Pword, Weight, and Focus. **(a)** NF: /Dī.ma ḥa.mat May.mū.nah min Na.wāl/. **(b)** IF: /Dī.ma ḥa.mat May.mū.nah min Na.wāl/. **(c)** CF: /Dī.ma ḥa.mat May.mū.nah min Na.wāl/. **(d)** NF: /May.mū.nah ja.bat Mi.nī.rah li Na.wāl/. **(e)** IF: /May.mū.nah ja.bat Mi.nī.rah li Na.wāl/. **(f)** CF: /May.mū.nah ja.bat Mi.nī.rah li Na.wāl/. **(g)** NF: /May.mū.nah wad.dat Na.wāl li Lī.na/. **(h)** IF: /May.mū.nah wad.dat Na.wāl li Lī.na/. **(i)** CF: /May.mū.nah wad.dat Na.wāl li Lī.na/.

**Figure 11:**
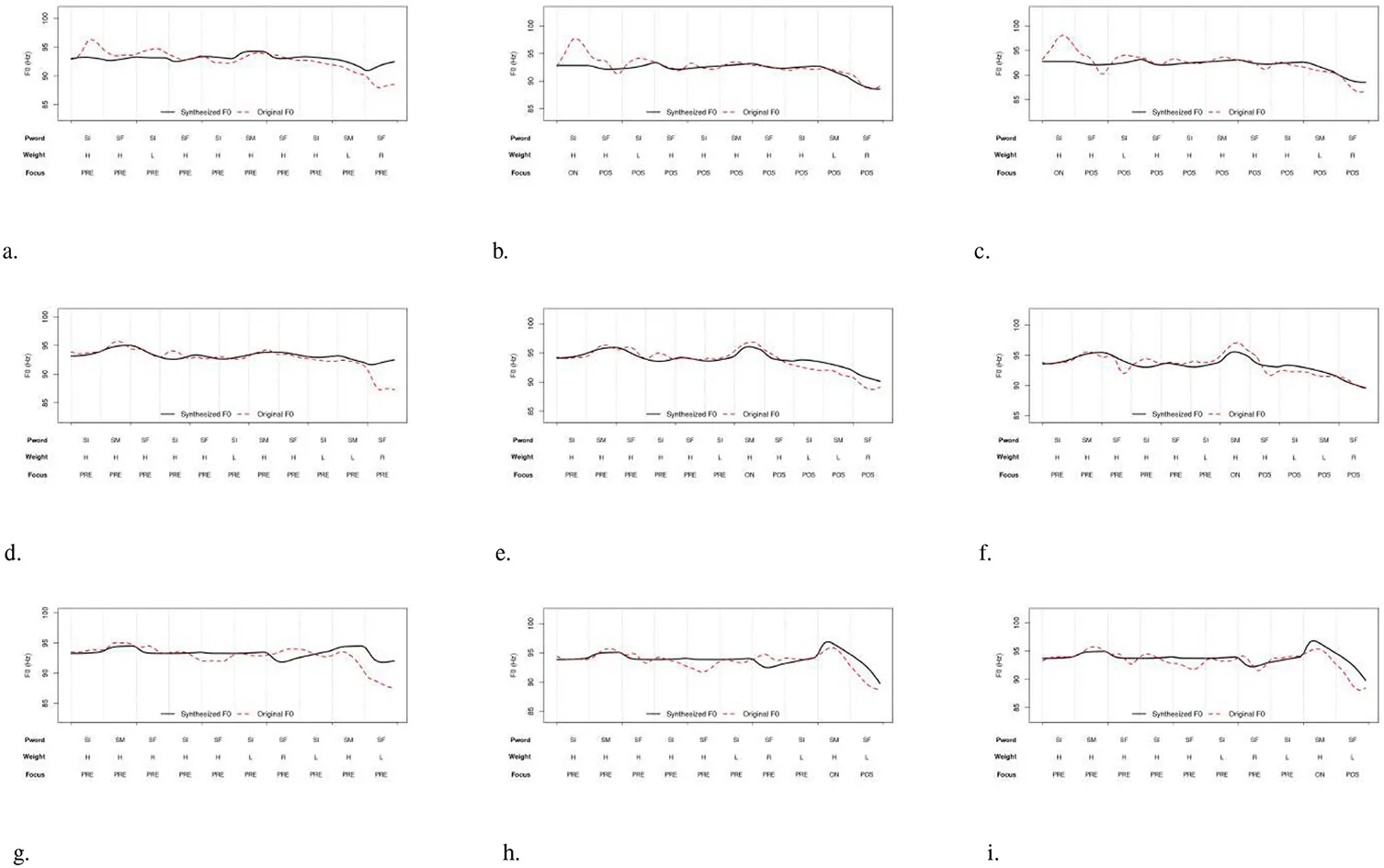
Mean *F*_0_ contours for the original (red dashed line) and synthesis (black solid line) are shown for **(a)** NF, **(b)** IF, and **(c)** CF. In each panel, the contour is averaged across 50 utterances, representing 10 speakers with five repetitions each for the relevant target sentence. The synthesized contour was produced from the learned PENTAtrainer parameters. The vertical dashed lines mark interval boundaries, and the tiers below the contours display the functional annotations used in synthesis: Pword, Weight, and Focus. **(a)** NF: /Dī.ma ḥa.mat May.mū.nah min Na.wāl/. **(b)** IF: /Dī.ma ḥa.mat May.mū.nah min Na.wāl/. **(c)** CF: /Dī.ma ḥa.mat May.mū.nah min Na.wāl/. **(d)** NF: /May.mū.nah ja.bat Mi.nī.rah li Na.wāl/. **(e)** IF: /May.mū.nah ja.bat Mi.nī.rah li Na.wāl/. **(f)** CF: /May.mū.nah ja.bat Mi.nī.rah li Na.wāl/. **(g)** NF: /May.mū.nah wad.dat Na.wāl li Lī.na/. **(h)** IF: /May.mū.nah wad.dat Na.wāl li Lī.na/. **(i)** CF: /May.mū.nah wad.dat Na.wāl li Lī.na/.

**Figure 12:**
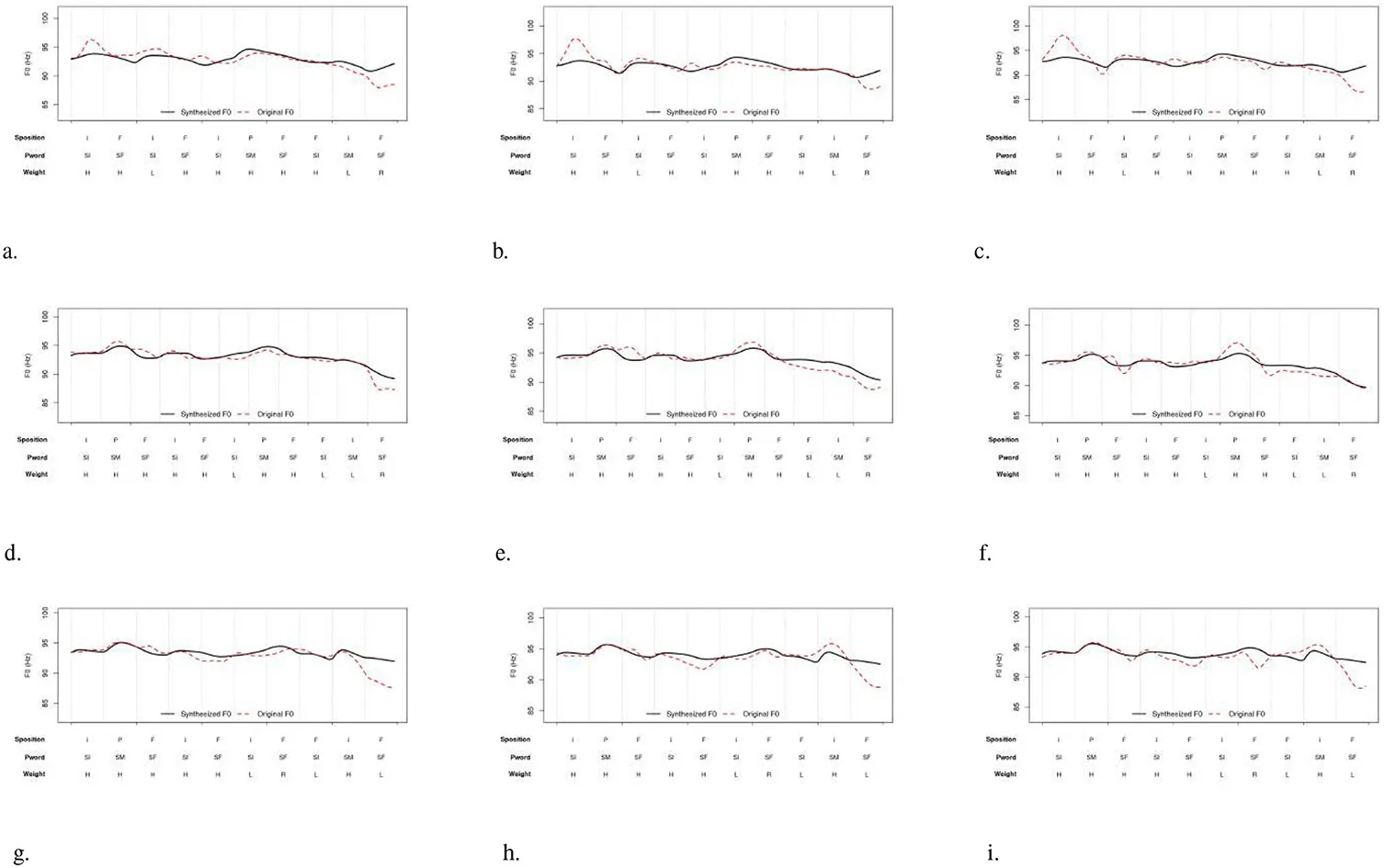
Mean *F*_0_ contours for the original (red dashed line) and synthesis (black solid line) are shown for **(a)** NF, **(b)** IF, and **(c)** CF. In each panel, the contour is averaged across 50 utterances, representing 10 speakers with five repetitions each for the relevant target sentence. The synthesized contour was produced from the learned PENTAtrainer parameters. The vertical dashed lines mark interval boundaries, and the tiers below the contours display the functional annotations used in synthesis: Sposition, SPword, and Weight. **(a)** NF: /Dī.ma ḥa.mat May.mū.nah min Na.wāl/. **(b)** IF: /Dī.ma ḥa.mat May.mū.nah min Na.wāl/. **(c)** CF: /Dī.ma ḥa.mat May.mū.nah min Na.wāl/. **(d)** NF: /May.mū.nah ja.bat Mi.nī.rah li Na.wāl/. **(e)** IF: /May.mū.nah ja.bat Mi.nī.rah li Na.wāl/. **(f)** CF: /May.mū.nah ja.bat Mi.nī.rah li Na.wāl/. **(g)** NF: /May.mū.nah wad.dat Na.wāl li Lī.na/. **(h)** IF: /May.mū.nah wad.dat Na.wāl li Lī.na/. **(i)** CF: /May.mū.nah wad.dat Na.wāl li Lī.na/.

**Figure 13:**
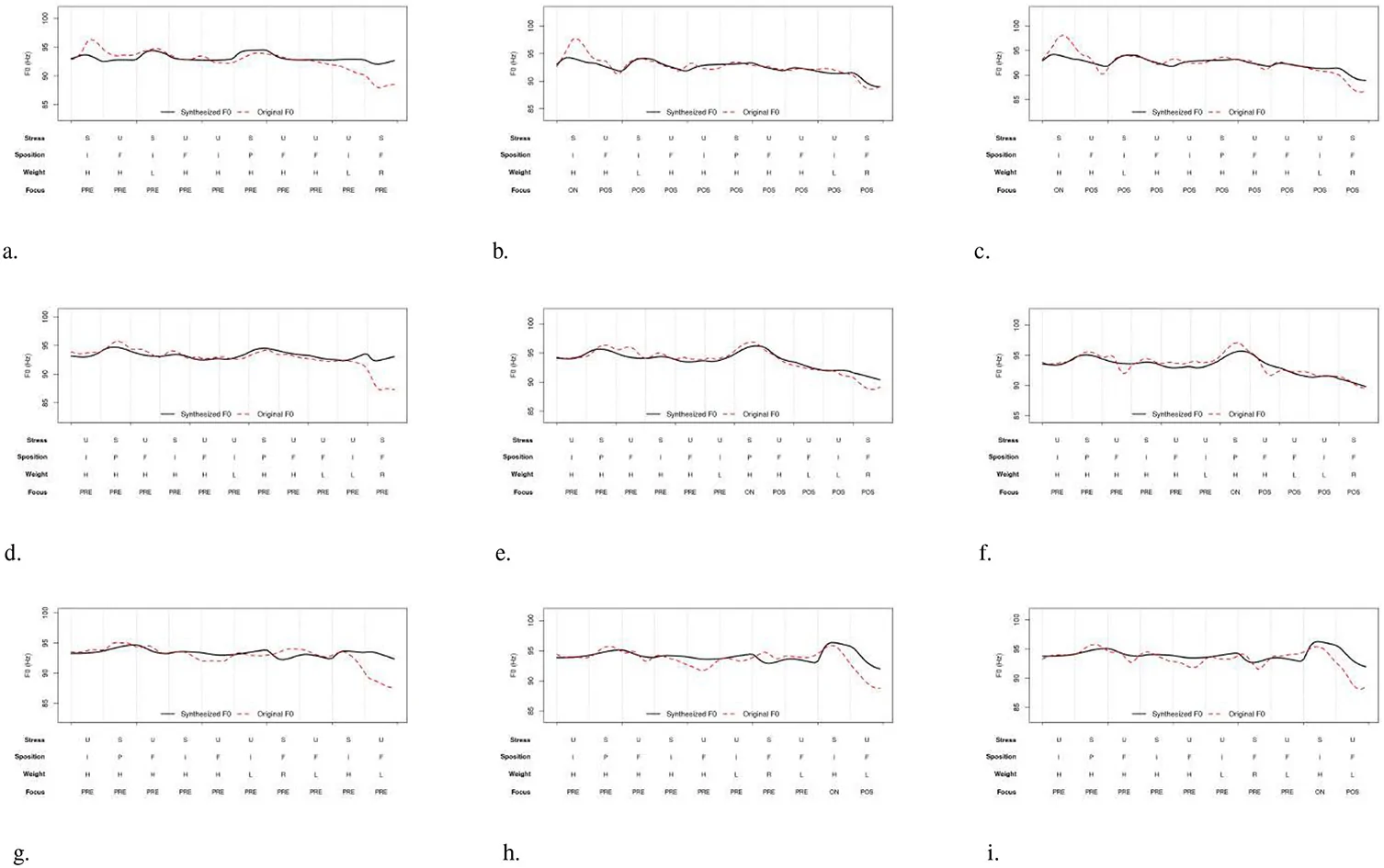
Mean *F*_0_ contours for the original (red dashed line) and synthesis (black solid line) are shown for **(a)** NF, **(b)** IF, and **(c)** CF. In each panel, the contour is averaged across 50 utterances, representing 10 speakers with five repetitions each for the relevant target sentence. The synthesized contour was produced from the learned PENTAtrainer parameters. The vertical dashed lines mark interval boundaries, and the tiers below the contours display the functional annotations used in synthesis: Stress, Sposition, Weight, and Focus. **(a)** NF: /Dī.ma ḥa.mat May.mū.nah min Na.wāl/. **(b)** IF: /Dī.ma ḥa.mat May.mū.nah min Na.wāl/. **(c)** CF: /Dī.ma ḥa.mat May.mū.nah min Na.wāl/. **(d)** NF: /May.mū.nah ja.bat Mi.nī.rah li Na.wāl/. **(e)** IF: /May.mū.nah ja.bat Mi.nī.rah li Na.wāl/. **(f)** CF: /May.mū.nah ja.bat Mi.nī.rah li Na.wāl/. **(g)** NF: /May.mū.nah wad.dat Na.wāl li Lī.na/. **(h)** IF: /May.mū.nah wad.dat Na.wāl li Lī.na/. **(i)** CF: /May.mū.nah wad.dat Na.wāl li Lī.na/.

**Figure 14:**
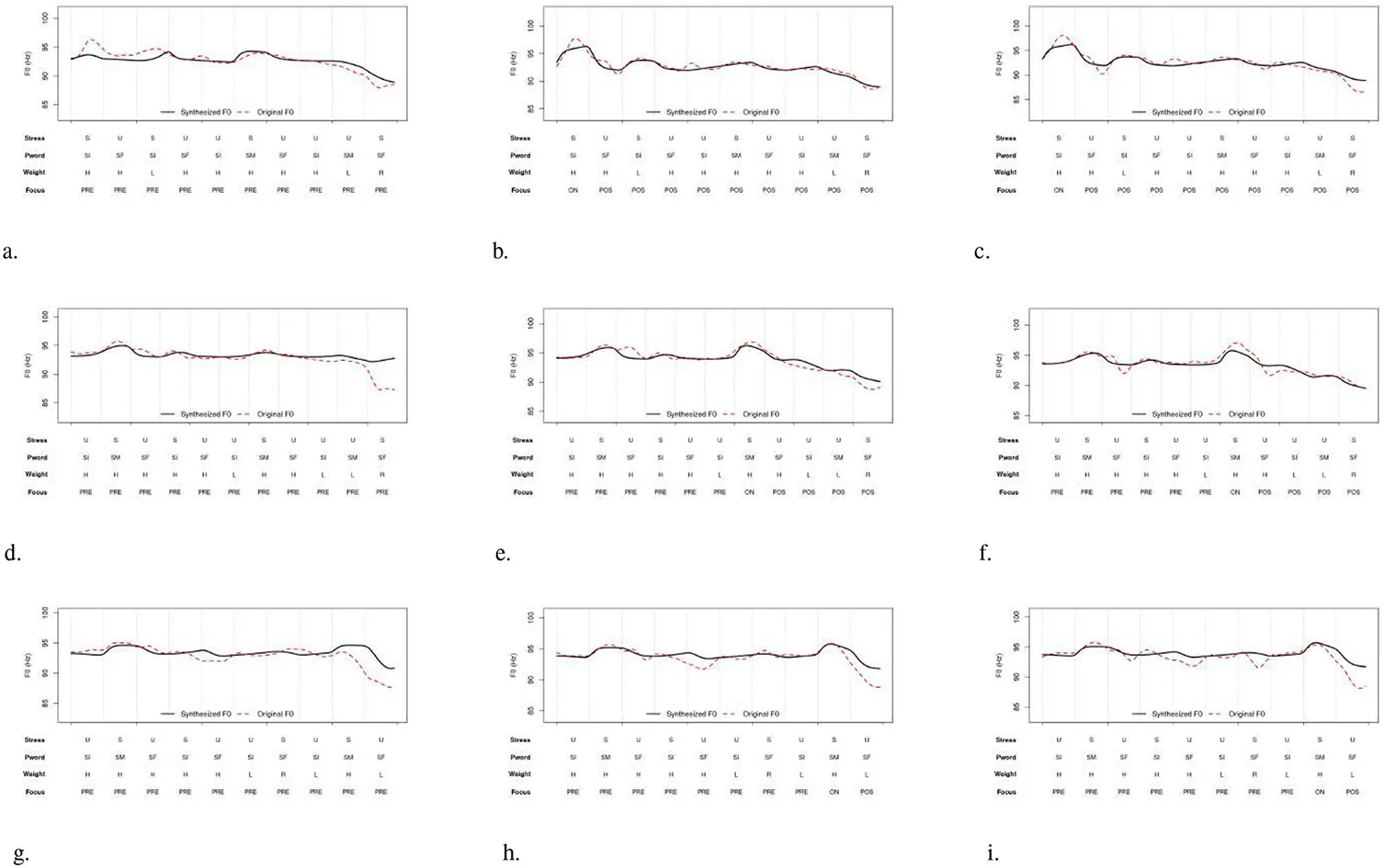
Mean *F*_0_ contours for the original (red dashed line) and synthesis (black solid line) are shown for **(a)** NF, **(b)** IF, and **(c)** CF. In each panel, the contour is averaged across 50 utterances, representing 10 speakers with five repetitions each for the relevant target sentence. The synthesized contour was produced from the learned PENTAtrainer parameters. The vertical dashed lines mark interval boundaries, and the tiers below the contours display the functional annotations used in synthesis: Stress, Pword = SPword, Weight, and Focus. **(a)** NF: /Dī.ma ḥa.mat May.mū.nah min Na.wāl/. **(b)** IF: /Dī.ma ḥa.mat May.mū.nah min Na.wāl/. **(c)** CF: /Dī.ma ḥa.mat May.mū.nah min Na.wāl/. **(d)** NF: /May.mū.nah ja.bat Mi.nī.rah li Na.wāl/. **(e)** IF: /May.mū.nah ja.bat Mi.nī.rah li Na.wāl/. **(f)** CF: /May.mū.nah ja.bat Mi.nī.rah li Na.wāl/. **(g)** NF: /May.mū.nah wad.dat Na.wāl li Lī.na/. **(h)** IF: /May.mū.nah wad.dat Na.wāl li Lī.na/. **(i)** CF: /May.mū.nah wad.dat Na.wāl li Lī.na/.

**Figure 15:**
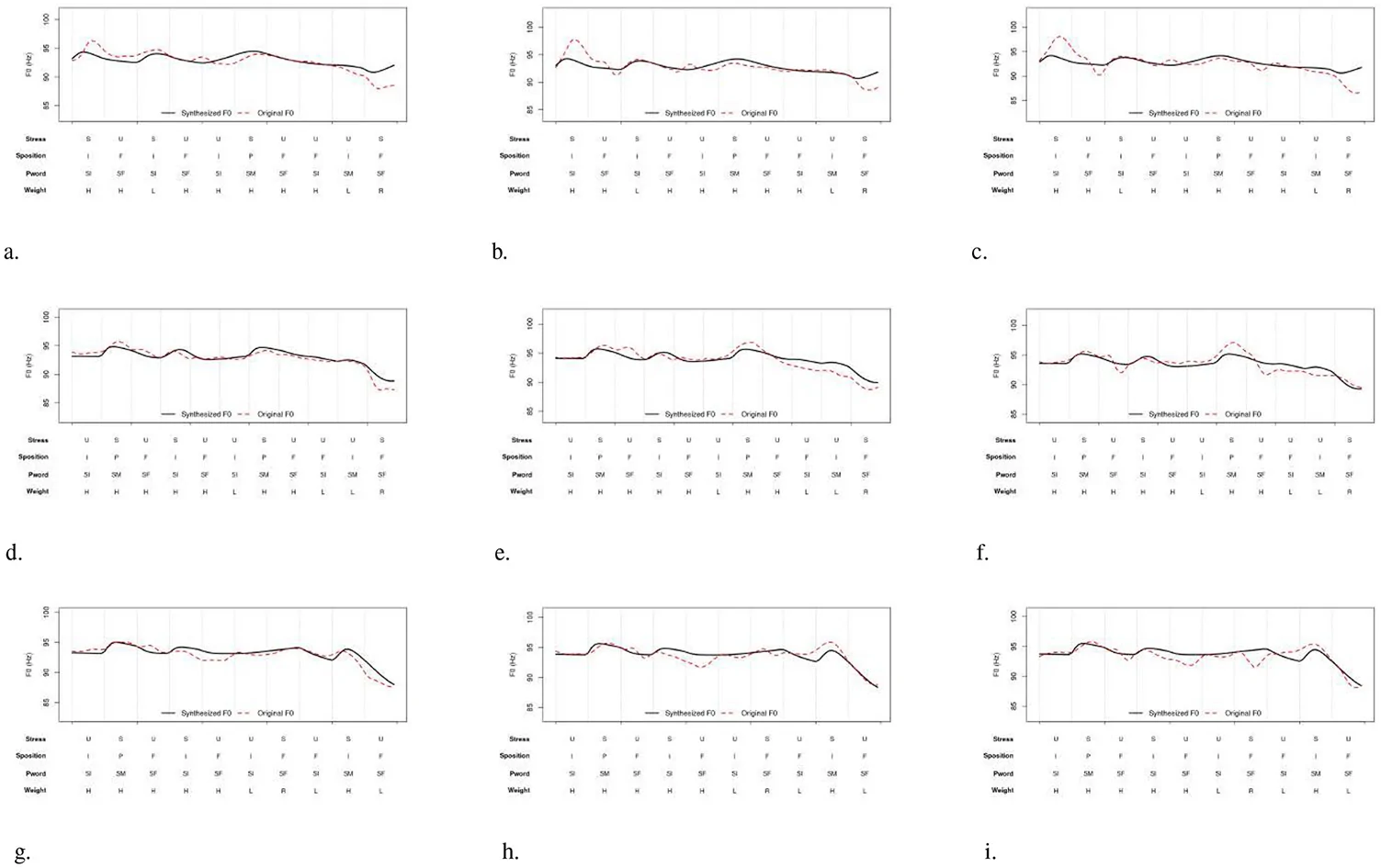
Mean *F*_0_ contours for the original (red dashed line) and synthesis (black solid line) are shown for **(a)** NF, **(b)** IF, and **(c)** CF. In each panel, the contour is averaged across 50 utterances, representing 10 speakers with five repetitions each for the relevant target sentence. The synthesized contour was produced from the learned PENTAtrainer parameters. The vertical dashed lines mark interval boundaries, and the tiers below the contours display the functional annotations used in synthesis: Stress, Sposition, Pword = SPword, Weight. **(a)** NF: /Dī.ma ḥa.mat May.mū.nah min Na.wāl/. **(b)** IF: /Dī.ma ḥa.mat May.mū.nah min Na.wāl/. **(c)** CF: /Dī.ma ḥa.mat May.mū.nah min Na.wāl/. **(d)** NF: /May.mū.nah ja.bat Mi.nī.rah li Na.wāl/. **(e)** IF: /May.mū.nah ja.bat Mi.nī.rah li Na.wāl/. **(f)** CF: /May.mū.nah ja.bat Mi.nī.rah li Na.wāl/. **(g)** NF: /May.mū.nah wad.dat Na.wāl li Lī.na/. **(h)** IF: /May.mū.nah wad.dat Na.wāl li Lī.na/. *(i)* CF: /May.mū.nah wad.dat Na.wāl li Lī.na/.

**Figure 16:**
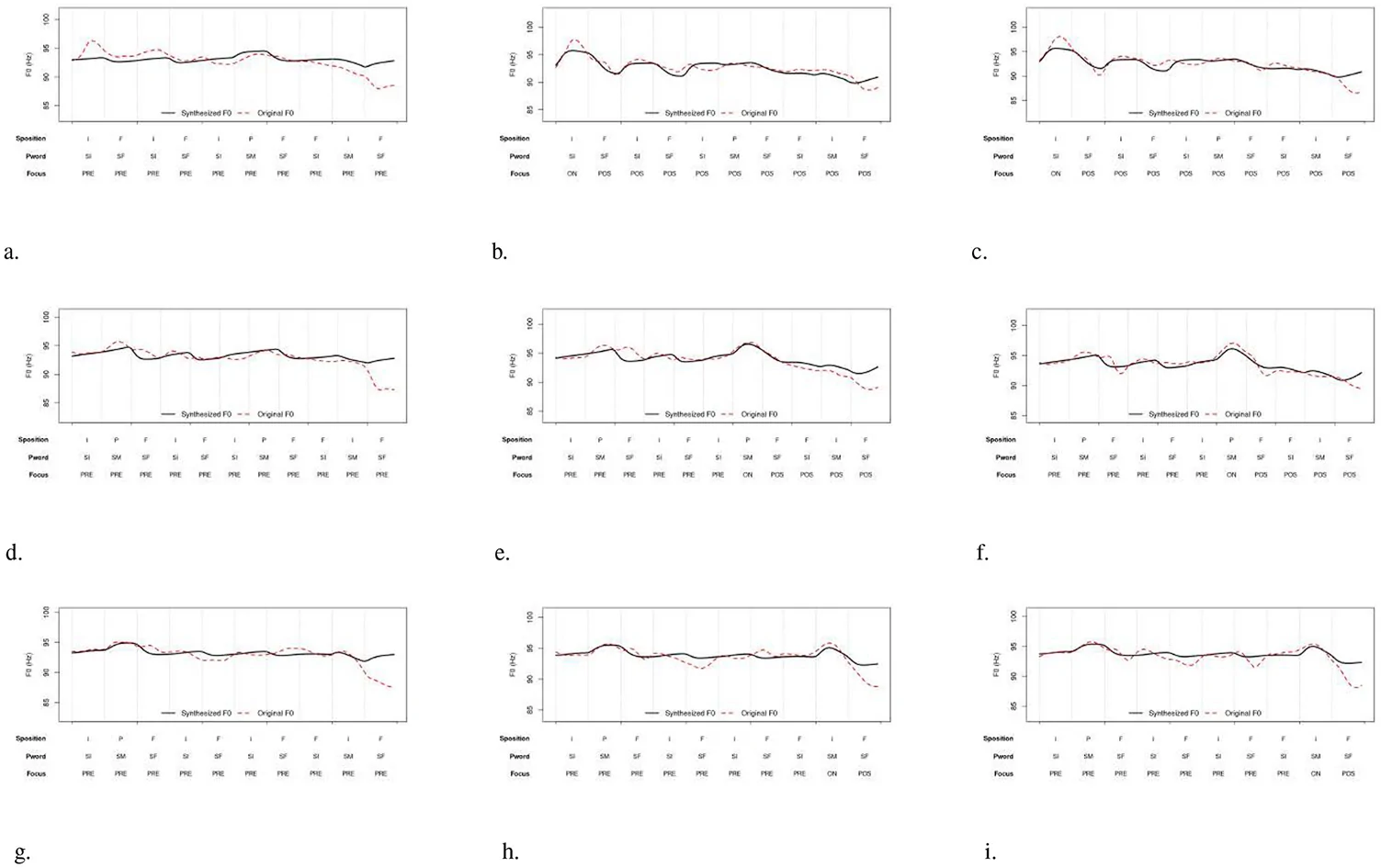
Mean *F*_0_ contours for the original (red dashed line) and synthesis (black solid line) are shown for **(a)** NF, **(b)** IF, and **(c)** CF. In each panel, the contour is averaged across 50 utterances, representing 10 speakers with five repetitions each for the relevant target sentence. The synthesized contour was produced from the learned PENTAtrainer parameters. The vertical dashed lines mark interval boundaries, and the tiers below the contours display the functional annotations used in synthesis: Sposition, Pword = SPword, and Focus. **(a)** NF: /Dī.ma ḥa.mat May.mū.nah min Na.wāl/. **(b)** IF: /Dī.ma ḥa.mat May.mū.nah min Na.wāl/. **(c)** CF: /Dī.ma ḥa.mat May.mū.nah min Na.wāl/. **(d)** NF: /May.mū.nah ja.bat Mi.nī.rah li Na.wāl/. **(e)** IF: /May.mū.nah ja.bat Mi.nī.rah li Na.wāl/. **(f)** CF: /May.mū.nah ja.bat Mi.nī.rah li Na.wāl/. **(g)** NF: /May.mū.nah wad.dat Na.wāl li Lī.na/. **(h)** IF: /May.mū.nah wad.dat Na.wāl li Lī.na/. **(i)** CF: /May.mū.nah wad.dat Na.wāl li Lī.na/.

**Figure 17:**
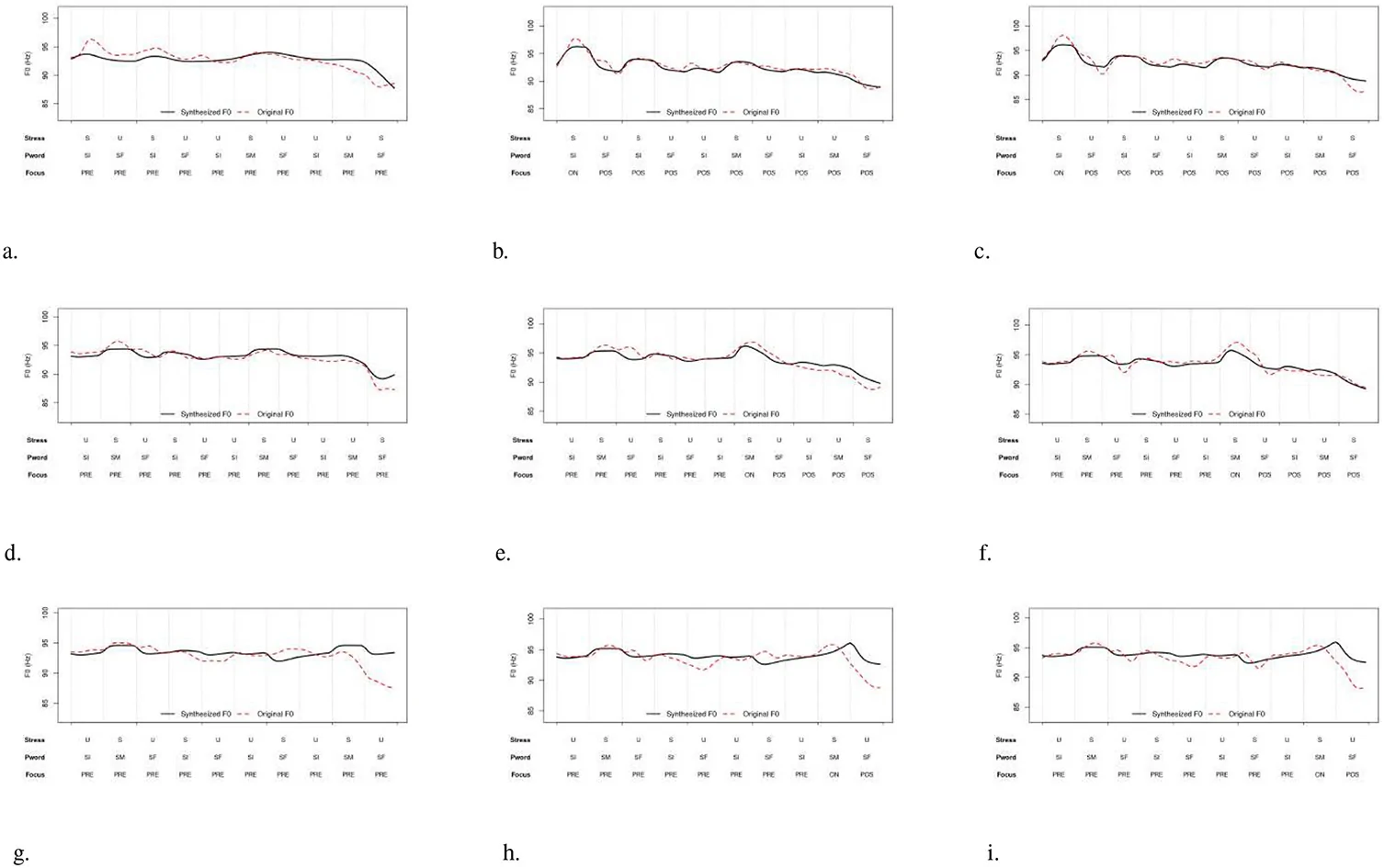
Mean *F*_0_ contours for the original (red dashed line) and synthesis (black solid line) are shown for **(a)** NF, **(b)** IF, and **(c)** CF. In each panel, the contour is averaged across 50 utterances, representing 10 speakers with five repetitions each for the relevant target sentence. The synthesized contour was produced from the learned PENTAtrainer parameters. The vertical dashed lines mark interval boundaries, and the tiers below the contours display the functional annotations used in synthesis: Stress, Pword = SPword, and Focus. **(a)** NF: /Dī.ma ḥa.mat May.mū.nah min Na.wāl/. **(b)** IF: /Dī.ma ḥa.mat May.mū.nah min Na.wāl/. **(c)** CF: /Dī.ma ḥa.mat May.mū.nah min Na.wāl/. **(d)** NF: /May.mū.nah ja.bat Mi.nī.rah li Na.wāl/. **(e)** IF: /May.mū.nah ja.bat Mi.nī.rah li Na.wāl/. **(f)** CF: /May.mū.nah ja.bat Mi.nī.rah li Na.wāl/. **(g)** NF: /May.mū.nah wad.dat Na.wāl li Lī.na/. **(h)** IF: /May.mū.nah wad.dat Na.wāl li Lī.na/. **(i)** CF: /May.mū.nah wad.dat Na.wāl li Lī.na/.

Figure 10 shows the synthesized contours after excluding both Focus and SPword (RMSE = 2.9683, *r* = 0.3129). This condition produces the clearest overall deterioration relative to the full-function model, with both higher RMSE and substantially lower correlation. The synthesized contours remain at a relatively uniform pitch level and differ mainly in local rises and falls associated with stressed syllables. The most noticeable mismatches occur in the focus regions. In several focus conditions, prominent peaks associated with the focused constituents are strongly reduced or absent, as seen in Figures 10b, c, h, i. Differences are also visible in the post-focus portions of some contours, for example in Figures 10e, f. These substantial mismatches are consistent with the highest RMSE and lowest correlation observed among the evaluated ablation conditions.

Figure 11 shows the synthesized contours after excluding both Stress and Sposition (RMSE = 2.8705, *r* = 0.3830). The synthesized contours preserve the general shape of the original contours and reproduce many of the major rises and falls across the utterances. The most noticeable differences occur in local regions associated with stressed syllables, where several peaks are reduced or absent. Relative to the Focus+SPword ablation, the overall contour structure remains more intact, although correlation is lower than in the full-function model.

Figure 12 shows the synthesized contours after excluding both Focus and Stress (RMSE = 2.8687, *r* = 0.3896). In neutral-focus utterances, the synthesized contour generally follows the overall shape of the original contour, although it remains higher toward the end of some utterances and does not always reproduce the final fall, as seen in Figures 12a, g. In these examples, peaks associated with stressed syllables in the sentence-initial word are also lower in the synthesized contours. In the focused conditions, the broad contour shapes remain similar to the originals, but peaks on the focused words are frequently reduced. The post-focus and pre-focus portions generally preserve the broad trajectory of the original contours, although local differences remain visible in Figures 12b, c, e, f, h, i.

Figure 13 shows the synthesized contours after excluding SPword (RMSE = 2.8566, *r* = 0.3885). Noticeable mismatches occur in several utterances. In neutral focus, peaks associated with stressed syllables are often lower in the synthesized contours, and the sentence-final portion sometimes ends at a higher *F*_0_ than in the original contour. In the focused conditions, peaks on the focused words are likewise reduced in several examples. The broad trajectories of the pre-focus and post-focus regions are nevertheless often similar to those of the corresponding original contours. Although this ablation yields a slightly lower RMSE than the full-function model, its substantially lower correlation indicates poorer reproduction of contour shape.

Figure 14 shows the synthesized contours after excluding Sposition (RMSE = 2.8502, *r* = 0.4082). The synthesized contours generally reproduce the major rises, peaks, and phrase-final falls of the original contours. In neutral-focus utterances, several local peaks are lower in the synthesized contours, and the utterance-final *F*_0_ is sometimes higher than in the original contours, as seen in Figures 14a, d, g. In focused conditions, peaks associated with the focused words are also somewhat reduced. The pre-focus and post-focus portions of the synthesized contours remain broadly similar to the original trajectories. This condition produces a slightly lower RMSE than the full model but also a lower correlation, indicating mixed effects on predictive performance.

Figure 15 shows the synthesized contours after excluding Focus (RMSE = 2.8428, *r* = 0.4150). In neutral-focus utterances, mismatches are generally limited. In focused conditions, however, peaks associated with focused constituents are often lower in the synthesized contours than in the originals, as seen in Figures 15b, c, e, f, h, i. Some post-focus portions also remain at a higher *F*_0_ level than their original counterparts, whereas the pre-focus portions are generally similar. As with the Sposition ablation, RMSE is slightly lower than in the full-function model, but correlation is also lower, so the effect of removing Focus is not unambiguously captured by a single performance metric.

Figure 16 shows the synthesized contours after excluding both Stress and Weight (RMSE = 2.8330, *r* = 0.4273). The synthesized contours generally preserve the broad shape of the original contours, including major peaks, broad post-focus contour trajectories, and overall declination. The most noticeable differences occur in the magnitude and timing of local pitch movements. In several cases, the synthesized contours show smoother trajectories or pitch excursions that differ from those of the original contours. Nevertheless, overall correspondence remains relatively close, and the correlation is almost identical to that of the full-function model.

Figure 17 shows the synthesized contours after excluding both Weight and Sposition (RMSE = 2.8281, *r* = 0.4165). The synthesized contours reproduce much of the overall shape of the original contours, including major peaks, valleys, and broad pitch movements associated with the different focus conditions. Differences are most apparent in the magnitude and timing of several excursions, particularly around prominent peaks and rapid transitions. The lower RMSE but lower correlation relative to the full-function model again indicates that the two evaluation measures do not yield a single, unambiguous ordering of model quality.

Figure 18 shows the synthesized contours after excluding Stress (RMSE = 2.8067, *r* = 0.4322). The synthesized contours generally follow the overall shape of the original contours in both neutral-focus and focused conditions. Some differences remain, particularly in the final portion of the utterance, where the synthesized contour may remain higher than the original contour. Importantly, this ablation produces both a lower RMSE and a slightly higher correlation than the full-function model. The present evaluation therefore does not provide evidence that including Stress as an independent functional tier improves predictive performance. Its information may be partly redundant with that encoded by other tiers, although the present analysis does not establish the source of this effect.

**Figure 18:**
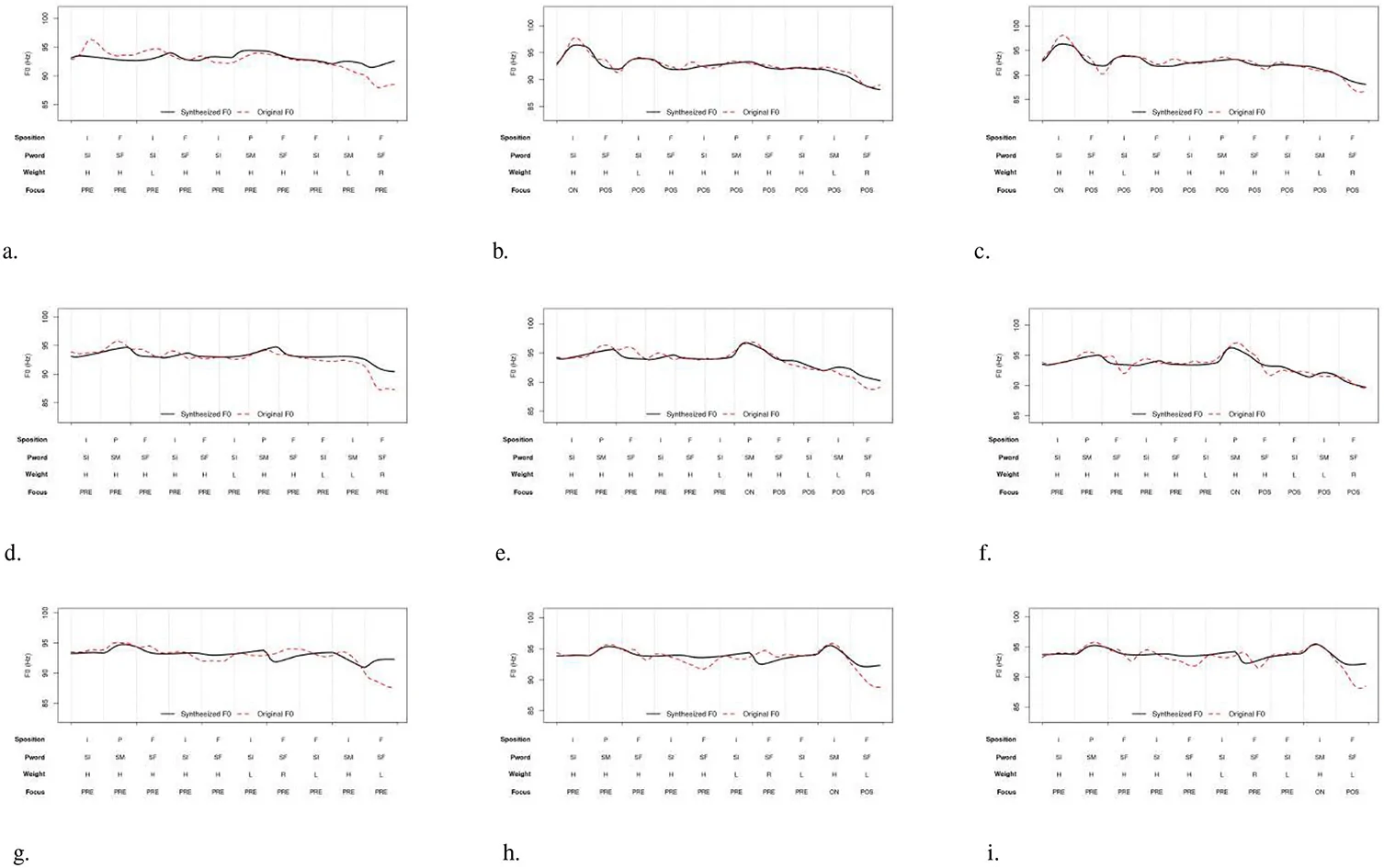
Mean *F*_0_ contours for the original (red dashed line) and synthesis (black solid line) are shown for **(a)** NF, **(b)** IF, and **(c)** CF. In each panel, the contour is averaged across 50 utterances, representing 10 speakers with five repetitions each for the relevant target sentence. The synthesized contour was produced from the learned PENTAtrainer parameters. The vertical dashed lines mark interval boundaries, and the tiers below the contours display the functional annotations used in synthesis: Sposition, Pword = SPword, Weight, and Focus. **(a)** NF: /Dī.ma ḥa.mat May.mū.nah min Na.wāl/. **(b)** IF: /Dī.ma ḥa.mat May.mū.nah min Na.wāl/. **(c)** CF: /Dī.ma ḥa.mat May.mū.nah min Na.wāl/. **(d)** NF: /May.mū.nah ja.bat Mi.nī.rah li Na.wāl/. **(e)** IF: /May.mū.nah ja.bat Mi.nī.rah li Na.wāl/. **(f)** CF: /May.mū.nah ja.bat Mi.nī.rah li Na.wāl/. **(g)** NF: /May.mū.nah wad.dat Na.wāl li Lī.na/. **(h)** IF: /May.mū.nah wad.dat Na.wāl li Lī.na/. **(i)** CF: /May.mū.nah wad.dat Na.wāl li Lī.na/.

Figure 19 shows the synthesized contours after excluding Weight (RMSE = 2.8023, *r* = 0.4432). The synthesized contours closely match the originals across the illustrated utterances. Some differences occur in utterance-final *F*_0_, where the synthesized contours are occasionally higher than their original counterparts, but the broad contour shapes remain highly similar. This condition yields the lowest RMSE and highest correlation among the models evaluated. Accordingly, the present results do not support interpreting Weight as a positive contributor to predictive performance in this modeling configuration. Its inclusion may encode information already represented by other functional tiers or increase model complexity without improving generalization, although this possibility would require additional testing to establish formally.

**Figure 19:**
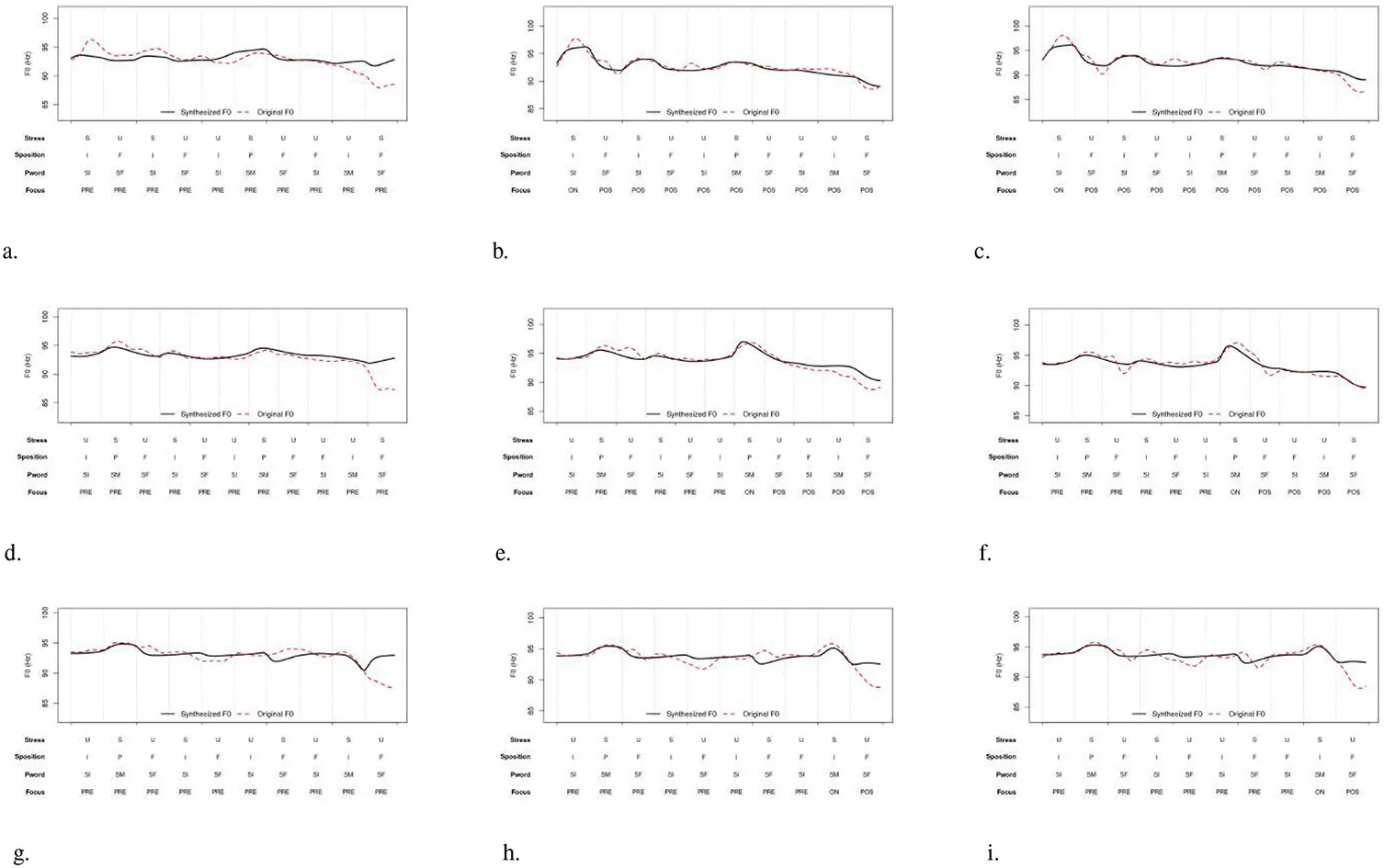
Mean *F*_0_ contours for the original (red dashed line) and synthesis (black solid line) are shown for **(a)** NF, **(b)** IF, and **(c)** CF. In each panel, the contour is averaged across 50 utterances, representing 10 speakers with five repetitions each for the relevant target sentence. The synthesized contour was produced from the learned PENTAtrainer parameters. The vertical dashed lines mark interval boundaries, and the tiers below the contours display the functional annotations used in synthesis: Stress, Sposition, Pword = SPword, and Focus. **(a)** NF: /Dī.ma ḥa.mat May.mū.nah min Na.wāl/. **(b)** IF: /Dī.ma ḥa.mat May.mū.nah min Na.wāl/. **(c)** CF: /Dī.ma ḥa.mat May.mū.nah min Na.wāl/. **(d)** NF: /May.mū.nah ja.bat Mi.nī.rah li Na.wāl/. **(e)** IF: /May.mū.nah ja.bat Mi.nī.rah li Na.wāl/. **(f)** CF: /May.mū.nah ja.bat Mi.nī.rah li Na.wāl/. **(g)** NF: /May.mū.nah wad.dat Na.wāl li Lī.na/. **(h)** IF: /May.mū.nah wad.dat Na.wāl li Lī.na/. **(i)** CF: /May.mū.nah wad.dat Na.wāl li Lī.na/.

From a PENTAtrainer perspective, the ablation results do not support a simple ordinal hierarchy in which every annotated tier makes a positive and independently measurable contribution to *F*_0_ generation. The clearest and most consistent deterioration occurs when Focus and SPword are removed together, yielding both the highest RMSE and the lowest correlation. This indicates that information jointly represented by these two tiers is particularly important for reconstructing the observed contours. Removal of SPword, Focus, Sposition, and several two-function combinations produces mixed effects, typically involving slightly lower RMSE but lower correlation than the full-function model. Their relative contributions therefore cannot be ordered unambiguously from the two evaluation metrics alone. Importantly, removing Stress or Weight slightly improves both RMSE and correlation relative to the full-function model. These results indicate that the present data do not support treating Stress or Weight as positive contributors to predictive performance in this configuration; instead, their information may be partly redundant with that encoded by other functional layers or may increase model complexity without improving generalization. The ablation analysis therefore provides the strongest evidence for the joint importance of Focus and SPword, while the contributions of the remaining tiers are more conditional and metric-dependent.

### 3.4 Discussion

The full-function PENTAtrainer model reproduced the main characteristics of the original *F*_0_ contours. In all focus conditions, the synthesized contours captured the location of the focused syllable, the associated *F*_0_ rise, and the broad contour patterns in the post-focus regions. Although the synthesized contours were generally smoother and showed smaller pitch excursions than the original contours, they closely followed the overall contour shape. The learned parameter inventory was also highly uneven across focus regions. Only three parameter combinations were required in the on-focus region, whereas twelve and nine combinations were learned for the pre- and post-focus regions, respectively. This difference reflects the more restricted phonological composition of the focused syllables in the corpus.

The ablation analyses did not support a simple ordinal hierarchy of functional contribution. The clearest and most consistent deterioration occurred when both Focus and SPword were excluded, resulting in the highest RMSE and the lowest correlation among the evaluated conditions. By contrast, excluding SPword, Focus, or Sposition individually produced mixed effects, with slightly lower RMSE but lower correlation relative to the full-function model. Removing Stress or Weight slightly improved both evaluation measures, with the No Weight condition yielding the lowest RMSE and highest correlation. These results therefore indicate that the effects of the individual functional tiers are conditional and metric-dependent. The strongest evidence concerns the joint contribution of the information encoded by Focus and SPword, with SPword representing syllable position within the prosodic word. The visual contour comparisons are consistent with this interpretation: the most substantial deviations occur when Focus and SPword are removed together, whereas the contours obtained after removing Weight remain particularly close to the originals.

## 4 Overall discussion

The present study addressed two main aims. The first was to determine whether focus in Qassimi Arabic is associated with systematic prosodic variation across on-focus, pre-focus, and post-focus regions in terms of *F*_0_, intensity, and duration. The second aim was to identify the functional factors that contribute to the formation of intonation within the PENTA framework and to determine their relative importance.

With respect to the first aim, the results show that Qassimi Arabic marks focus primarily through prosodic enhancement of the focused word, accompanied by post-focus intensity attenuation and only limited modification of the post-focus *F*_0_ contour. This general pattern is broadly consistent with previous work showing that Arabic dialects differ substantially in the realization of focus across on-focus, pre-focus, and post-focus regions (Chahal, 2001, 2003; Benkirane, 1998; Yeou, 2004; Yeou et al., 2007; Norlin, 1989; Hellmuth, 2011; Al Hasan and Mahanta, 2022; Alzaidi et al., 2019; Alzaidi, 2022; Alzaidi et al., 2023, 2025; Alzamil and Hellmuth, 2022). In the present study, the broader post-focus analyses reveal significant attenuation of mean intensity following both information focus and contrastive focus, but no significant broad effects on excursion size or maximum, mean, or minimum *F*_0_. The localized analysis of the sentence-penultimate post-focus word additionally reveals a significant reduction in excursion size following contrastive focus relative to both neutral focus and information focus, whereas information focus does not differ significantly from neutral focus. The Qassimi Arabic pattern should therefore be characterized as cue-specific and position-specific post-focus modification rather than as general post-focus *F*_0_ compression. Accordingly, the present findings do not support an unqualified classification of Qassimi Arabic as a +PFC variety. Compared with Arabic dialects in which broader post-focus *F*_0_ compression has been reported, the evidence in Qassimi Arabic is more restricted; nevertheless, Qassimi Arabic shows reliable post-focus intensity attenuation and a localized contrastive-focus-specific reduction in excursion size, whereas Makkan Arabic has been reported to show little evidence of systematic post-focus compression (Alzaidi, 2022).

The analyses did not detect a consistent acoustic distinction between information focus and contrastive focus across the measures and sentence positions examined. Most direct comparisons between the two focus conditions did not reach statistical significance, although contrastive focus showed a significantly higher maximum *F*_0_ in the sentence-penultimate on-focus position and a significantly smaller excursion size in the localized sentence-penultimate post-focus word. The absence of significance in the remaining comparisons should not be interpreted as evidence that the two focus types are acoustically equivalent. Rather, the present findings indicate that reliable differentiation was limited to particular measures and positions under the controlled experimental conditions employed here. Related studies of Makkan, Iraqi, Najdi, and Jizani Arabic have likewise reported limited or measure-specific differences between information focus and contrastive focus (Alzaidi, 2022; Alzaidi et al., 2025; Alzamil and Hellmuth, 2022). By contrast, stronger differentiation has been reported for Hijazi and Emirati Arabic, where contrastive focus exhibits greater on-focus prominence or stronger post-focus modification (Alzaidi et al., 2019, 2023).

As for the second aim, the full-function PENTAtrainer model successfully reproduced the major properties of the original *F*_0_ contours in Qassimi Arabic. The synthesized contours captured focus-induced prominence, declination, phrase-final lowering, and condition-dependent variation in the post-focus contour, and closely matched the major characteristics of the original contours across the focus conditions. These findings are consistent with those of Xu and Prom-On (2014), who demonstrated that PENTAtrainer accurately predicts original *F*_0_ contours in Thai, Mandarin Chinese, and American English, and with those of Alzaidi et al. (2023), who showed that the model successfully captures the major intonational characteristics of Emirati Arabic. Together, these studies demonstrate that the PENTAtrainer framework generalizes well across typologically different languages, including Arabic dialects.

The function-removal analyses did not support a simple ordinal hierarchy of positive functional contribution. The clearest and most consistent deterioration occurred when both Focus and SPword were excluded, producing the highest RMSE and the lowest correlation among the evaluated conditions. Removing SPword, Focus, or Sposition individually produced mixed effects, with slightly lower RMSE but lower correlation relative to the full-function model. By contrast, removing Stress or Weight slightly improved both RMSE and correlation. Unlike Xu and Prom-On (2014), who evaluated functional importance mainly through hypothesis-testing by manipulating annotation layers, and Alzaidi et al. (2023), who examined the contribution of individual functions through single-function exclusion, the present study compares a broader set of single- and paired-function ablations under cross-validation. These results indicate that the effects of the annotated functional tiers are conditional and metric-dependent rather than forming a single hierarchy of importance. The strongest and most consistent evidence concerns the information made available jointly by Focus and SPword, with SPword representing the position of the syllable within the prosodic word. The improved performance following removal of Stress or Weight may reflect redundancy with information encoded by other tiers or additional model complexity, although the present analysis does not establish the source of these effects. Overall, the findings extend previous PENTAtrainer research by showing that the contribution of individual functional layers to contour reconstruction cannot necessarily be characterized by a simple additive ranking.

Although the present study demonstrates that PENTAtrainer successfully learned the major prosodic properties of focus in Qassimi Arabic, model performance based on RMSE and Pearson's correlation was lower than that reported by Alzaidi et al. (2023). One important methodological difference between the two studies concerns the evaluation design. In Alzaidi et al. (2023), model generalization was evaluated using speakers who were excluded from the training data, whereas the present study employed leave-one-sentence-group-out cross-validation, with each held-out sentence group excluded entirely from model training. Thus, the two studies evaluated different dimensions of generalization: generalization across speakers in the former and generalization across unseen sentence forms in the present study. The present results do not establish that one evaluation procedure is inherently more demanding than the other. It is possible that generalization across unseen sentence and prosodic contexts introduces sources of variability that differ from those involved in generalization across speakers, but this remains a hypothesis. Future research directly comparing speaker-held-out and sentence-held-out evaluation within the same dataset and modeling configuration is needed to determine their relative effects on predictive performance.

## 5 Conclusion

The present study demonstrates that focus prosody in Qassimi Arabic is systematically organized and can be investigated effectively through complementary acoustic and computational approaches within the PENTA framework. The acoustic analyses show that focus is realized primarily through enhancement of the focused constituent, especially through raising of the *F*_0_ register and, in some sentence positions, expansion of pitch excursion. The pre-focus region remains comparatively stable, while the broader post-focus domain shows reliable intensity attenuation following both information focus and contrastive focus. Evidence for post-focus *F*_0_ modification is more restricted: broad post-focus measures of excursion size and maximum, mean, and minimum *F*_0_ do not differ significantly across focus conditions, while a localized reduction in excursion size is found following contrastive focus at the sentence-penultimate post-focus word. The findings therefore support a characterization of Qassimi Arabic as exhibiting cue-specific and position-specific post-focus modification rather than general post-focus *F*_0_ compression, and they do not justify an unqualified classification of the dialect as a +PFC variety.

The computational analyses further show that PENTAtrainer can reproduce major characteristics of the observed *F*_0_ contours, including on-focus prominence, declination, phrase-final lowering, and condition-dependent variation in the post-focus contour. Function-removal analyses show that the clearest deterioration in predictive performance occurs when Focus and SPword, the tier encoding syllable position within the prosodic word, are removed jointly, whereas the effects of removing the remaining functional tiers are conditional and metric-dependent. More broadly, the findings reinforce the substantial cross-dialectal variability found in Arabic focus prosody while demonstrating the value of computational modeling for examining how multiple communicative functions jointly shape intonation. Taken together, the acoustic and computational results are consistent with the central PENTA assumption that intonation emerges through the coordinated interaction of communicative functions encoded in target space.

## Data Availability

The raw data supporting the conclusions of this article will be made available by the authors, without undue reservation.

## Footnotes

1 In the results tables, NF refers to neutral focus, IF to information focus, and CF to contrastive focus.

2 (Alzaidi 2022) followed a similar approach by analyzing all post-focus content words and then examining the stressed syllable of the sentence-penultimate word, where post-focus compression was expected to be strongest.

## References

[B1] Al Hasan M. Mahanta S. (2022). “The intonational phonology of Syrian Arabic: a preliminary analysis,” in Proceedings of speech prosody 2022 (Lisbon: International Speech Communication Association (ISCA)), 190–194. doi: 10.21437/SpeechProsody.2022-39

[B2] Al Motairi S. (2015). An Optimality-Theoretic Analysis of Syllable Structure in Qassimi Arabic (Ph.D. thesis). Ypsilanti, MI: Eastern Michigan University.

[B3] Alnuqaydan A. (2023). The Interaction of Stress and Phonological Variation in Qassimi Arabic (Ph.D. thesis). Salt Lake City, UT: The University of Utah.

[B4] Alqahtani M. S. M. (2014). Syllable Structure and Related Processes in Optimality Theory: An Examination of Najdi Arabic (Ph.D. thesis). Newcastle University. Available online at: http://theses.ncl.ac.uk/jspui/handle/10443/2757 (Accessed September 26, 2026).

[B5] Alrashed A. S. (2018). Descriptive analysis of Qassimi Arabic: Phonemic vowels, syllable structure and epenthetic vowels, and affrication. Long Beach, CA: California State University.

[B6] Alzaidi M. Saeed S. Bani Younes M. (2025). Phonetic realizations of focus in declarative intonation in Iraqi Arabic. Speech Commun. 170:103203. doi: 10.1016/j.specom.2025.103203

[B7] Alzaidi M. S. (2022). Makkan Arabic does not have post-focus compression: a production and perception study. Phonetica 79, 247–308. doi: 10.1515/phon-2022-2021 35918784

[B8] Alzaidi M. S. Xu Y. Xu A. (2019). Prosodic encoding of focus in Hijazi Arabic. Speech Commun. 106, 127–149. doi: 10.1016/j.specom.2018.12.006

[B9] Alzaidi M. S. A. (2014). Information Structure and Intonation in Hijazi Arabic (Ph.D. thesis). Colchester: University of Essex.

[B10] Alzaidi M. S. A. (2024). Syntax, phonology, or both? Jordan J. Modern Lang. Literat. 16, 627–650. doi: 10.47012/jjmll.16.3.4

[B11] Alzaidi M. S. A. Alshammari A. Alahmari S. Al Sultan H. Hassan A. Q. A. Salama A. S. (2024). Harnessing Corpus Linguistics and Data-Driven Learning Approach for Arabic Multi-Class Dialect Detection and Classification. Fractals, 2540007. Available online at: https://www.worldscientific.com/doi/abs/10.1142/S0218348X25400079 (Accessed September 26, 2026).

[B12] Alzaidi M. S. A. Bani Younes M. A. S. Saaed S. A. AlAbabneh B. (in press). Investigation of focus in Bedouin Hijazi Arabic. Jordan J. Modern Lang. Literat. Accepted for publication.

[B13] Alzaidi M. S. A. Xu Y. Xu A. Szreder M. (2023). Analysis and computational modelling of Emirati Arabic intonation-A preliminary study. J. Phon. 98:101236. doi: 10.1016/j.wocn.2023.101236

[B14] Alzamil A. Hellmuth S. (2022). “The realization of different structural focus conditions in Saudi Arabic dialects,” in Proceedings of the 1st international conference on tone and intonation (TAI2021) (Sønderborg: ISCA-INST Speech Communication Association), 195–199. doi: 10.21437/TAI.2021-40

[B15] Ardali M. T. Xu Y. (2012). “Phonetic realization of prosodic focus in Persian,” in Proceedings of the 6th international conference on speech prosody (SP2012) (Shanghai: Tongji University Press), 326–329. doi: 10.21437/SpeechProsody.2012-83

[B16] Audacity Team (2022). Audacity(R): Free Audio Editor and Recorder [Computer application]. Available online at: https://audacityteam.org (Accessed September 26, 2026).

[B17] Bates D. Mächler M. Bolker B. Walker S. (2015). Fitting linear mixed-effects models using lme4. J. Stat. Softw. 67, 1–48. doi: 10.18637/jss.v067.i01

[B18] Benkirane T. (1998). Intonation in Western Arabic (Morocco). Inton. Syst. 345–359.

[B19] Boersma P. Weenink D. (2023). PRAAT: Doing Phonetics by Computer (Amsterdam: University of Amsterdam).

[B20] Breen M. Fedorenko E. Wagner M. Gibson E. (2010). Acoustic correlates of information structure. Lang. Cogn. Process. 25(7-9):1044–1098. doi: 10.1080/01690965.2010.504378

[B21] Brown G. Hellmuth S. (2022). Computational modelling of segmental and prosodic levels of analysis for capturing variation across Arabic dialects. Speech Commun. 141, 80–92. doi: 10.1016/j.specom.2022.05.003

[B22] Chafe W. (1976). Givenness, contrastiveness, definiteness, subjects, topics and point of view. Subject Topic/Acad. 30, 25–55.

[B23] Chahal D. (2001). Modeling the Intonation of Lebanese Arabic Using the Autosegmental-Metrical Framework: A Comparison with English (Ph.D. thesis). Melbourne: University of Melbourne.

[B24] Chahal D. (2003). “Phonetic cues to prominence in Lebanese Arabic,” in Proceedings of the 15th international congress of phonetic sciences (Barcelona: Universitat Autònoma de Barcelona), 2067–2070.

[B25] Chahal D. Hellmuth S. (2014). “The intonation of Lebanese and Egyptian Arabic,” in Prosodic Typology II, 1st Edn., ed. S.-A. Jun (Oxford: Oxford University Press), 365–404. doi: 10.1093/acprof:oso/9780199567300.003.0013

[B26] Chen L. Li X. Yang Y. (2012). Focus, newness and their combination: processing of information structure in discourse. PLoS ONE 7:e42533. doi: 10.1371/journal.pone.0042533 PMC3422350 22912708

[B27] Chen L. Wang L. Yang Y. (2014). Distinguish between focus and newness: an erp study. J. Neurolinguistics 31, 28–41. doi: 10.1016/j.jneuroling.2014.06.002

[B28] Chen S.-,w. Wang B. Xu Y. (2009). “Closely related languages, different ways of realizing focus,” in Interspeech 2009 (Brighton: ISCA), 1007–1010. doi: 10.21437/Interspeech.2009-298

[B29] Chen Y. Xu Y. (2025). Sequential perception of tone and focus in parallel-a computational simulation. Speech Commun. 168:103173. doi: 10.1016/j.specom.2024.103173

[B30] Cooper W. E. Eady S. J. Mueller P. R. (1985). Acoustical aspects of contrastive stress in question-answer contexts. J. Acoust. Soc. Am. 77, 2142–2156. doi: 10.1121/1.392372 4019901

[B31] Diamond J. Bellwood P. (2003). Farmers and their languages: the first expansions. Science 300, 597–603. doi: 10.1126/science.1078208 12714734

[B32] Ditters E. (2013). “Issues in Arabic computational linguistics,” in The Oxford Handbook of Arabic Linguistics. (Oxford: Oxford University Press), 213. doi: 10.1093/oxfordhb/9780199764136.013.010_update_001

[B33] Downing L. J. Rialland A. (2016). Intonation in African Tone Languages. Berlin: Walter de Gruyter GmbH & Co KG. doi: 10.1515/9783110503524

[B34] Eady S. J. Cooper W. E. (1986). Speech intonation and focus location in matched statements and questions. J. Acoust. Soc. Am. 80, 402–415. doi: 10.1121/1.394091 3745672

[B35] Elfardy H. Diab M. (2013). “Sentence level dialect identification in Arabic,” in Proceedings of the 51st annual meeting of the association for computational linguistics (volume 2: short papers) (Sofia: Association for Computational Linguistics (ACL)), 456–461.

[B36] Féry C. Ishihara S. (2016). The Oxford Handbook of Information Structure. Oxford: Oxford University Press.

[B37] Gussenhoven C. (1984). On the Grammar and Semantics of Sentence Accents. Berlin; Boston, MA: De Gruyter Mouton. doi: 10.1515/9783110859263

[B38] Gussenhoven C. (2002). Intonation and Biology. Liber Amicorum Bernard Bichakjian (Festschrift for Bernard Bichakjian), 59–82. Available online at: http://gep.ruhosting.nl/carlos/bbichakjian.pdf (Accessed September 26, 2026).

[B39] Habash N. Sadat F. (2006). “Arabic preprocessing schemes for statistical machine translation,” in Proceedings of the human language technology conference of the NAACL, companion volume: short papers (New York, NY: Association for Computational Linguistics (ACL)), 49–52. doi: 10.3115/1614049.1614062

[B40] Habash N. Y. (2010). Introduction to Arabic Natural Language Processing. San Rafael, CA: Morgan & Claypool Publishers. doi: 10.1007/978-3-031-02139-8

[B41] Halliday M. a. K.. (1967). Notes on transitivity and theme in English: part 2. J. Linguist. 3, 199–244. doi: 10.1017/S0022226700016613

[B42] Hanssen J. E. G. Peters J. Gussenhoven C. (2008). “Prosodic effects of focus in Dutch declaratives,” in Proceedings of speech prosody (Campinas: International Speech Communication Association (ISCA)), 609–612. doi: 10.21437/SpeechProsody.2008-138

[B43] He X. Hanssen J. van Heuven V. J. Gussenhoven C. (2011). “Phonetic implementation must be learnt: native versus chinese realization of focus accent in Dutch,” in Proceedings of the 17th international congress of phonetic sciences (ICPhS XVII) (Hong Kong: University of Hong Kong), 843–846.

[B44] Hellmuth S. (2011). “Acoustic cues to focus and givenness in Egyptian Arabic,” in Instrumental studies in Arabic phonetics (Amsterdam: John Benjamins Publishing Company), 299–324. doi: 10.1075/cilt.319.14hel

[B45] Holes C. (1995). Modern Arabic: Structures, Functions, and Varieties. London; New York, NY: Longman.

[B46] Hothorn T. Bretz F. Westfall P. (2008). Simultaneous inference in general parametric models. Biom. J. 50, 346–363. doi: 10.1002/bimj.200810425 18481363

[B47] Hwang H. L. (2012). “Asymmetries between production, perception and comprehension of focus types in Japanese,” in Proceedings of speech prosody 2012 (Shanghai: Tongji University Press), 326–329. doi: 10.21437/SpeechProsody.2012-160

[B48] Ingham B. (1994). Najdi Arabic: Central Arabian. Amsterdam: John Benjamins Publishing. doi: 10.1075/loall.1

[B49] Ipek C. (2011). “Phonetic realization of focus with no on-focus pitch range expansion in Turkish,” in ICPhS (Hong Kong: City University of Hong Kong), 140–143.

[B50] Johnstone T. M. (1967). Aspects of syllabication in the spoken Arabic of'Anaiza. Bull. School Oriental Afr. Stud. 30, 1–16. doi: 10.1017/S0041977X00099067

[B51] Jun S.-A. Lee H.-J. (1998). “Phonetic and phonological markers of contrastive focus in Korean,” in ICSLP (Sydney: Australian Speech Science and Technology Association (ASSTA)). doi: 10.21437/ICSLP.1998-151

[B52] Khalifa S. Habash N. Abdulrahim D. Hassan S. (2016). “A large scale corpus of Gulf Arabic,” in Proceedings of the tenth international conference on language resources and evaluation (LREC'16) (Portorož: European Language Resources Association (ELRA)), 4282–4289. doi: 10.63317/4h6puu8bogss

[B53] Kirkpatrick S. Gelatt Jr C. D. Vecchi M. P. (1983). Optimization by simulated annealing. Science 220, 671–680. doi: 10.1126/science.220.4598.671 17813860

[B54] Kristensen L. B. Wang L. Petersson K. M. Hagoort P. (2013). The interface between language and attention: prosodic focus marking recruits a general attention network in spoken language comprehension. Cereb. Cortex 23, 1836–1848. doi: 10.1093/cercor/bhs164 22763170

[B55] Ladd R. (2008). Intonational Phonology, 2nd Edn. Cambridge: Cambridge University Press. doi: 10.1017/CBO9780511808814

[B56] Lambrecht K. (1994). Information Structure and Sentence Form: Topic, Focus, and the Mental Representations of Discourse Referents. Cambridge Studies in Linguistics. Cambridge: Cambridge University Press. doi: 10.1017/CBO9780511620607

[B57] Norlin K. (1989). A preliminary description of Cairo Arabic intonation of statements and questions. Speech Transm. Quart. Progress Status Rep. 1, 47–49.

[B58] O'Rourke E. (2012). The realization of contrastive focus in Peruvian Spanish intonation. Lingua 122, 494–510. doi: 10.1016/j.lingua.2011.10.002

[B59] Pasha A. Al-Badrashiny M. Diab M. T. El Kholy A. Eskander R. Habash N. . (2014). “MADAMIRA: a fast, comprehensive tool for morphological analysis and disambiguation of Arabic,” in LREC (Reykjavik: European Language Resources Association (ELRA)), vol. 14, 1094–1101. doi: 10.63317/3piegahzyagx

[B60] Pierrehumbert J. Hirschberg J. B. (1990). “The meaning of intonational contours in the interpretation of discourse,” in Intentions in Communication, eds. P. R. Cohen, J. Morgan, and M. E. Pollack (Cambridge, MA: MIT Press), 271–311. doi: 10.7551/mitpress/3839.003.0016

[B61] Pierrehumbert J. B. (1980). The Phonology and Phonetics of English Intonation (Ph.D. thesis). Cambridge, MA: Massachusetts Institute of Technology.

[B62] Prom-on S. Liu F. Xu Y. (2012). Post-low bouncing in Mandarin Chinese: acoustic analysis and computational modeling. J. Acoust. Soc. Am. 132, 421–432. doi: 10.1121/1.4725762 22779489

[B63] Prom-On S. Xu Y. Thipakorn B. (2009). Modeling tone and intonation in Mandarin and English as a process of target approximation. J. Acoust. Soc. Am. 125, 405–424. doi: 10.1121/1.3037222 19173427

[B64] R Core Team (2019). R: A Language and Environment for Statistical Computing. Vienna: R Core Team.

[B65] Sahkai H. Kalvik M.-L. Mihkla M. (2013). “Prosody of contrastive focus in Estonian,” in Proceedings of interspeech 2013 (Lyon: International Speech Communication Association (ISCA)), 315–319. doi: 10.21437/Interspeech.2013-91

[B66] Sityaev D. House J. (2003). “Phonetic and phonological correlates of broad, narrow and contrastive focus in English,” in Proceedings of the 15th international congress of phonetic sciences (Barcelona: Universitat Aut2noma de Barcelona), 1819–1822.

[B67] Wang B. Xu Y. Ding Q. (2017). Interactive prosodic marking of focus, boundary and newness in Mandarin. Phonetica 75, 24–56. doi: 10.1159/000453082 28595174

[B68] Xu Y. (1999). Effects of tone and focus on the formation and alignment of F0 contour. J. Phon. 27, 55–105. doi: 10.1006/jpho.1999.0086

[B69] Xu Y. (2004). “The PENTA model of speech melody: transmitting multiple communicative functions in parallel,” in Proceedings of from sound to sense: 50+years of discoveries in speech communication (MA: Massachusetts Institute of Technology (MIT)).

[B70] Xu Y. (2005). Speech melody as articulatorily implemented communicative functions. Speech Commun. 46, 220–251. doi: 10.1016/j.specom.2005.02.014

[B71] Xu Y. (2011a). “Post-focus compression: cross-linguistic distribution and historical origin,” in The 17th international congress of phonetic sciences (Hong Kong: University of Hong Kong), 152–155.

[B72] Xu Y. (2011b). Speech prosody: a methodological review. J. Speech Sci. 1, 85–115. doi: 10.20396/joss.v1i1.15014

[B73] Xu Y. (2013). “ProsodyPro–a tool for large-scale systematic prosody analysis,” in Proceedings of tools and resources for the analysis of speech prosody (Aix-en-Provence: Laboratoire Parole et Langage), 7–10.

[B74] Xu Y. (2019). “Prosody, tone, and intonation,” in The Routledge Handbook of Phonetics eds. William F. Katz and Peter F. Assmann (Abingdon: Routledge), 314–356. doi: 10.4324/9780429056253-13

[B75] Xu Y. (2024). Syllable as a synchronization mechanism that makes human speech possible. Brain Sci. 15:33. doi: 10.3390/brainsci15010033 PMC11763374 39851401

[B76] Xu Y. Chen S.-,w. Wang B. (2010). “Prosodic focus with post-focus compression: single or multiple origin?,” in Proceedings of The 2nd symposium on evolutionary linguistics (Tianjin: Nankai University).

[B77] Xu Y. Liu F. (2006). Tonal alignment, syllable structure and coarticulation: t oward an integrated model. Italian J. Linguist 18, 125–159.

[B78] Xu Y. Liu F. (2012). “Intrinsic coherence of prosodic and segmental aspects of speech,” in Understanding prosody – *the role of context, function, and communication* (Berlin: De Gruyter), 1–16. doi: 10.1515/9783110301465.1

[B79] Xu Y. Prom-On S. (2014). Toward invariant functional representations of variable surface fundamental frequency contours: synthesizing speech melody via model-based stochastic learning. Speech Commun. 57, 181–208. doi: 10.1016/j.specom.2013.09.013

[B80] Xu Y. Szu-wei Chen Wang B. (2012). Prosodic focus with and without post-focus compression: a typological divide within the same language family? Linguist. Rev. 29, 131–147. doi: 10.1515/tlr-2012-0006

[B81] Xu Y. Wang Q. E. (2001). Pitch targets and their realization: evidence from Mandarin Chinese. Speech Commun. 33, 319–337. doi: 10.1016/S0167-6393(00)00063-7

[B82] Xu Y. Xu C. X. (2005). Phonetic realization of focus in English declarative intonation. J. Phon. 33, 159–197. doi: 10.1016/j.wocn.2004.11.001

[B83] Yeou M. (2004). Effects of focus, position and syllable structure on f0 alignment patterns in Arabic. JEP-TALN, pages 19–22.

[B84] Yeou M. Embarki M. Al-Maqtari S. (2007). Contrastive focus and F0 patterns in three Arabic dialects. Nouveaux Cahiers Linguist. Fran. 28, 317–326.

[B85] Zerbian S. Genzel S. Kügler F. (2010). “Experimental work on prosodically-marked information structure in selected African languages (Afroasiatic and Niger-Congo),” in Speech prosody 2010-fifth international conference (Chicago, IL: International Speech Communication Association (ISCA)). doi: 10.21437/SpeechProsody.2010-86

