# Supplementary material for: Focus prosody in Qassimi Arabic: acoustic analysis and computational modeling
Source: Front Hum Neurosci. 2026 Oct 7;20:1928391. doi: 10.3389/fnhum.2026.1928391 (PMC13683374; doi:10.3389/fnhum.2026.1928391)
Supplement: Supplementary file 1 [file Data_Sheet_2.zip › Figure/map2026.pdf]

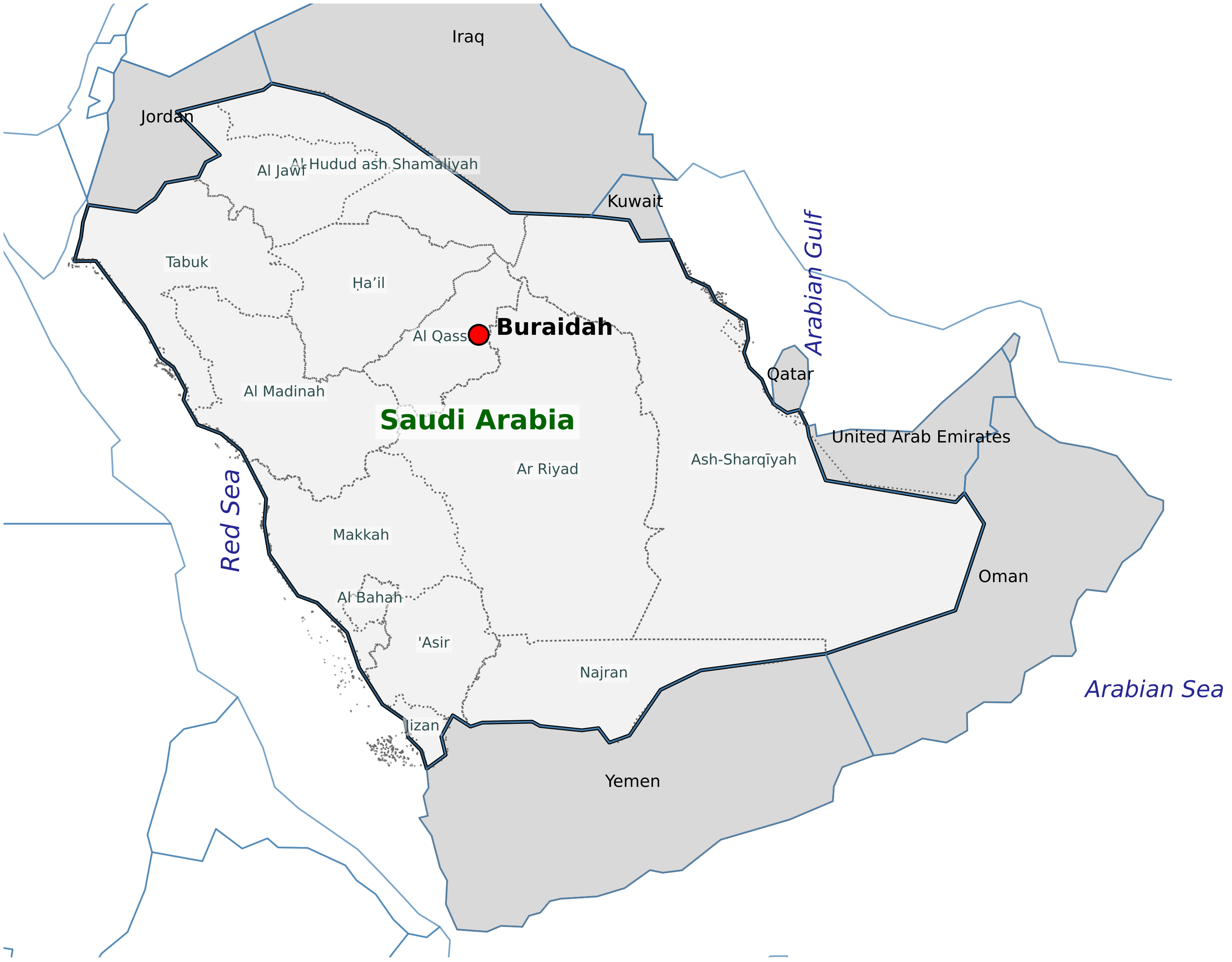
Iraq
Jordan
Al Jawf
Al Hudud ash Shamaliyah
Kuwait
Arabian Gulf
Tabuk
Ha’il
Buraidah
Al Qass
Qatar
Al Madinah
Saudi Arabia
United Arab Emirates
Ash-Sharqīyah
Ar Riyad
Red Sea
Makkah
Oman
Al Bahah
'Asir
Najran
Arabian Sea
Jizan
Yemen
