# Supplementary material for: Focus prosody in Qassimi Arabic: acoustic analysis and computational modeling
Source: Front Hum Neurosci. 2026 Oct 7;20:1928391. doi: 10.3389/fnhum.2026.1928391 (PMC13683374; doi:10.3389/fnhum.2026.1928391)
Supplement: Supplementary file 2 [file Supplementary_file_1.zip › Appendix.pdf]

Supplementary Material

Focus Prosody in Qassimi Arabic: Acoustic Analysis and Computational Modeling

Muhammad S. A. Alzaidi et al.

Frontiers in Human Neuroscience

Supplementary Appendix

Appendix A: R code for the mixed-effects analyses

The placeholder variable *AcousticMeasurement* in the models below was replaced with the acoustic parameter under investigation, namely excursion size, maximum F_0, mean F_0, minimum F_0, mean intensity, or duration. Separate models were fitted for each acoustic measure, sentence position, and prosodic region.

The null model included crossed random intercepts and by-focus random slopes for speaker and sentence but did not include focus condition as a fixed effect:

```
model_null <- lmer(
AcousticMeasurement ~ 1 +
(Focus | Subject) +
(Focus | Sentence),
data = analysis_data,
REML = FALSE,
control = lmerControl(
optimizer = "nloptwrap",
calc.derivs = TRUE
)
)
```

The full model had the same random-effects structure and additionally included focus condition as a fixed effect:

```
model_focus <- lmer(
AcousticMeasurement ~ Focus +
(Focus | Subject) +
(Focus | Sentence),
data = analysis_data,
REML = FALSE,
control = lmerControl(
optimizer = "nloptwrap",
calc.derivs = TRUE
```

```
)
)
```

The overall effect of focus condition was evaluated by comparing the null and full models using a likelihood-ratio test:

```
anova(model_null, model_focus)
```

Model singularity was examined using:

```
isSingular(model_focus, tol = 1e-4)
```

Convergence messages were inspected using:

```
model_focus@optinfo$conv$lme4$messages
```

When the likelihood-ratio test showed a significant omnibus effect of focus condition, Tukey-adjusted pairwise comparisons were conducted using the `glht` function from the `multcomp` package:

```
posthoc_model <- glht(
model_focus,
linfct = mcp(Focus = "Tukey")
)

summary(
posthoc_model,
test = adjusted("single-step")
)

confint(
posthoc_model,
level = 0.95
)
```

The pairwise comparisons therefore report unstandardized estimated differences, standard errors, simultaneously adjusted 95% confidence intervals, and single-step-adjusted p-values.

Appendix B: Distribution of acoustic measures

This appendix presents box plots illustrating the distribution of acoustic features across focus conditions (Neutral, Information, and Contrastive) for different sentential positions and prosodic regions. For each feature, the box represents the interquartile range (IQR), the horizontal line indicates the median, and whiskers extend to 1.5×IQR.

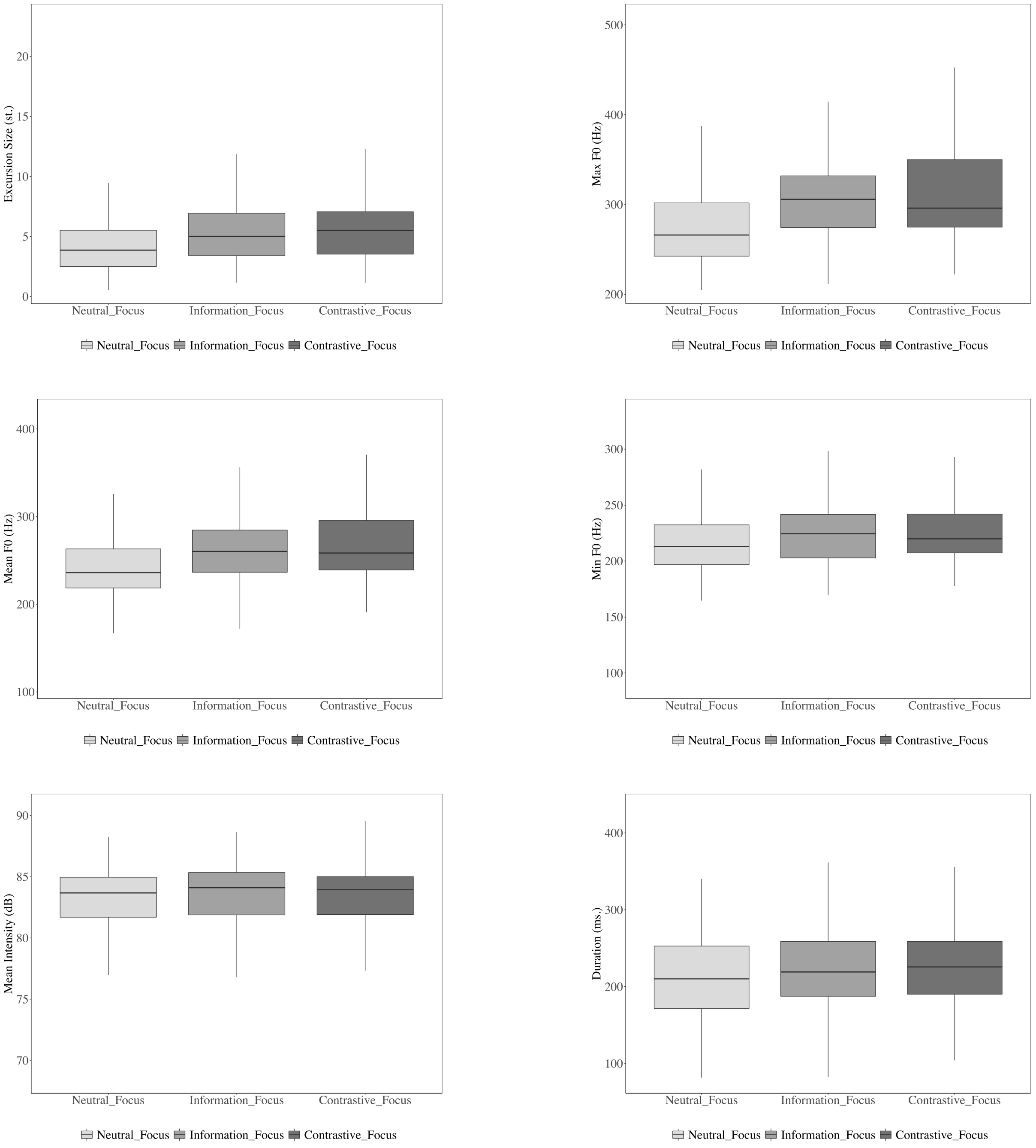

Figure 1: Initial focus — On-focus region.

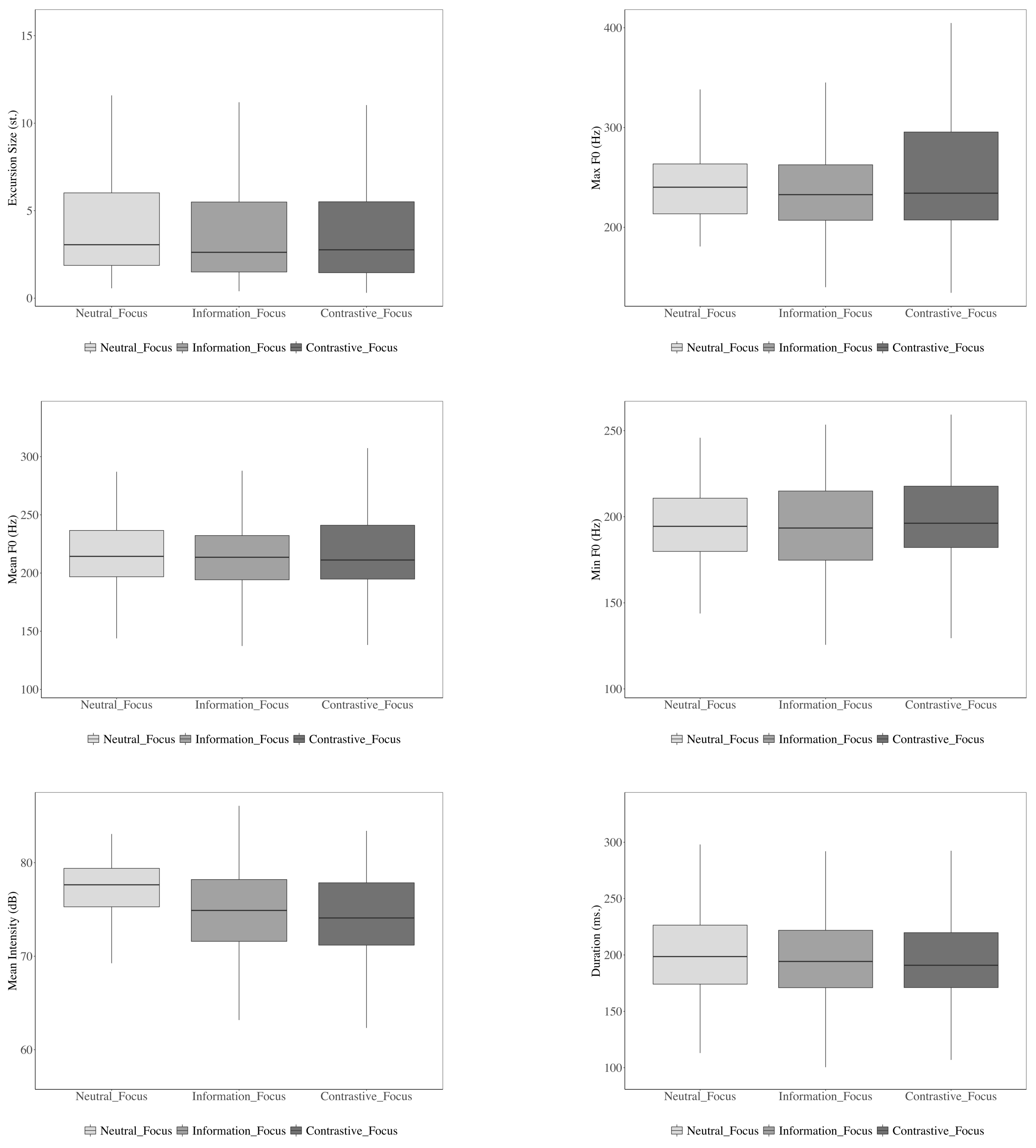

Figure 2: Initial focus — Post-focus region.

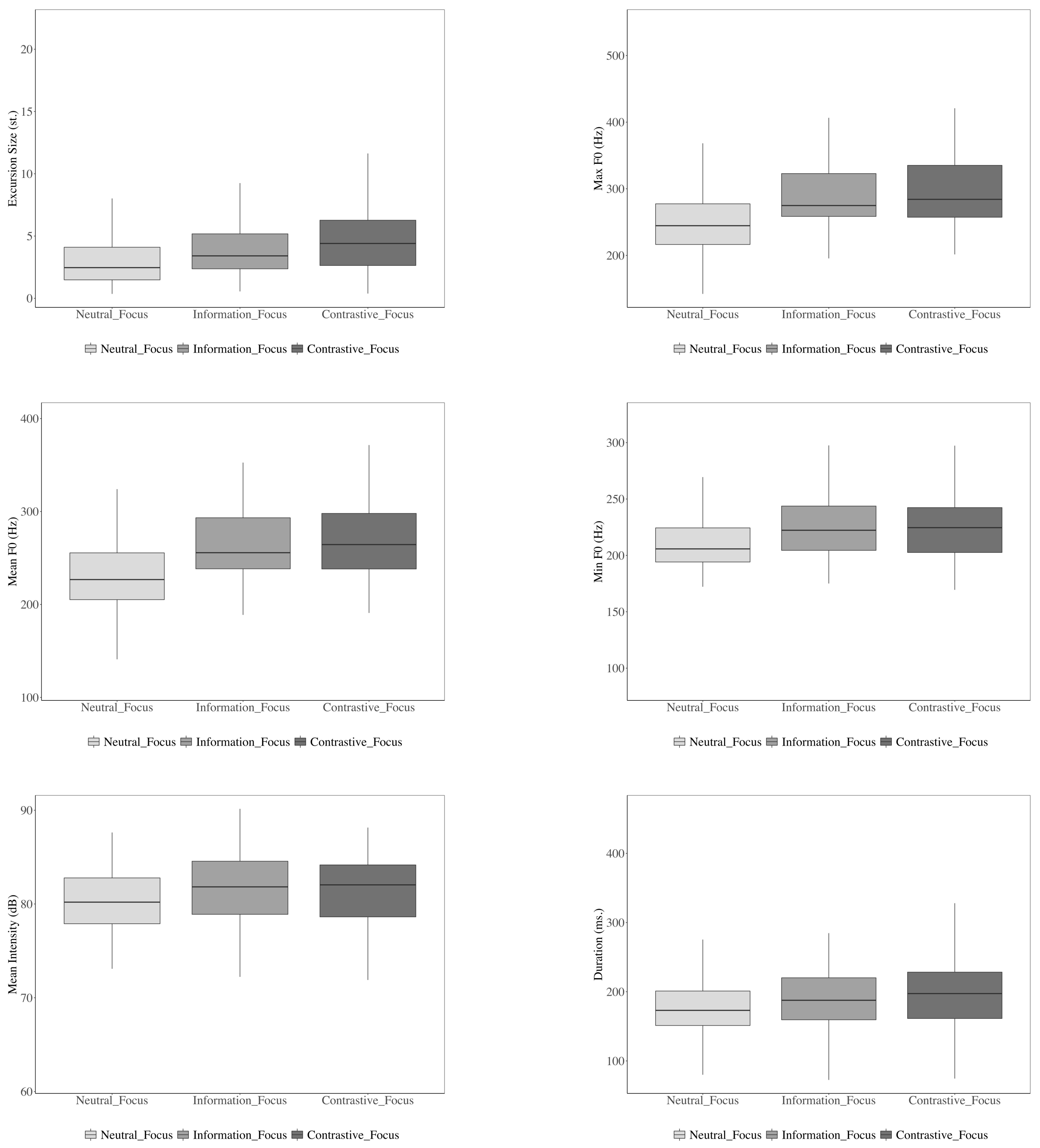

Figure 3: Penultimate focus — On-focus region.

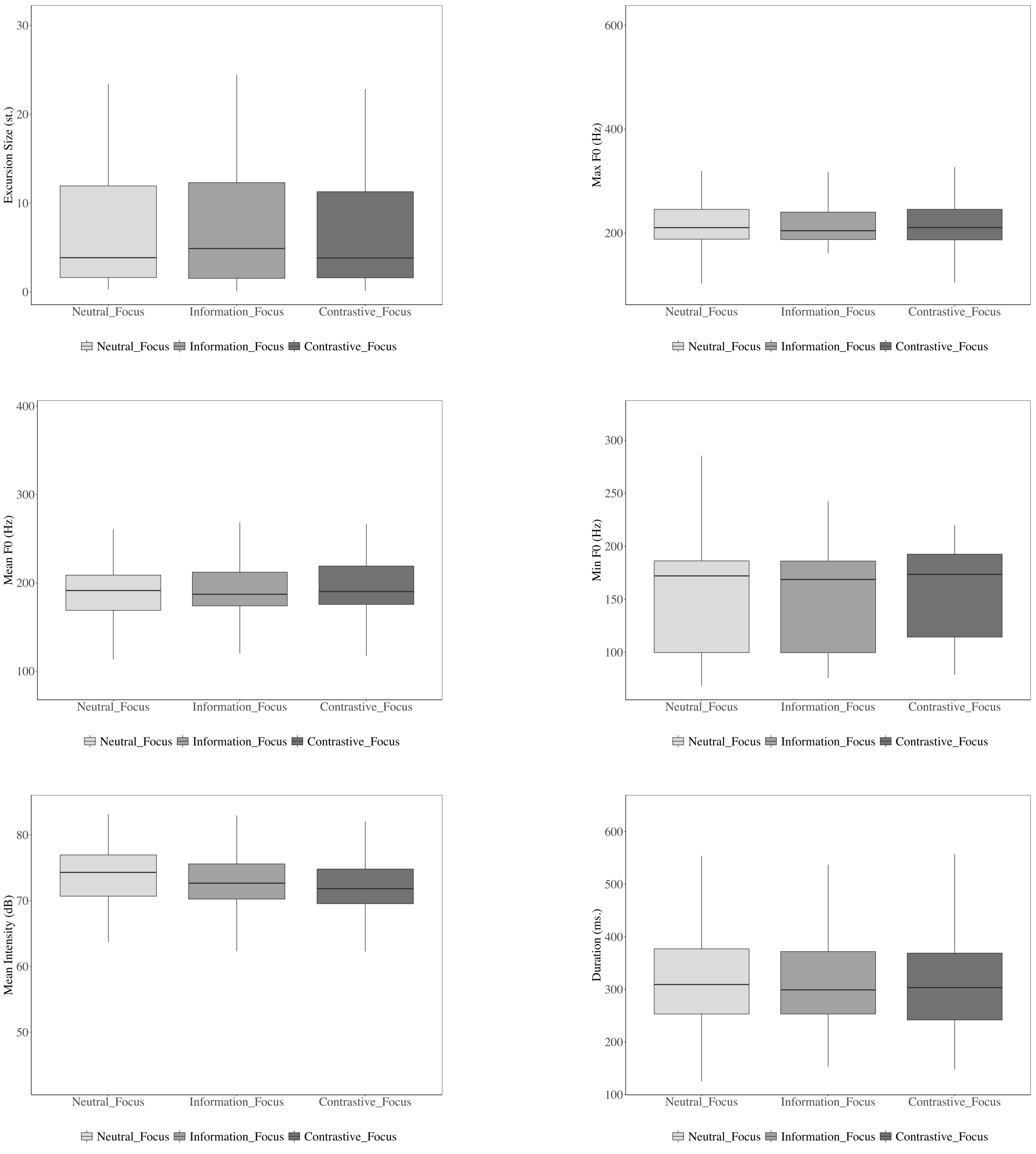

Figure 4: Penultimate focus — Post-focus region.

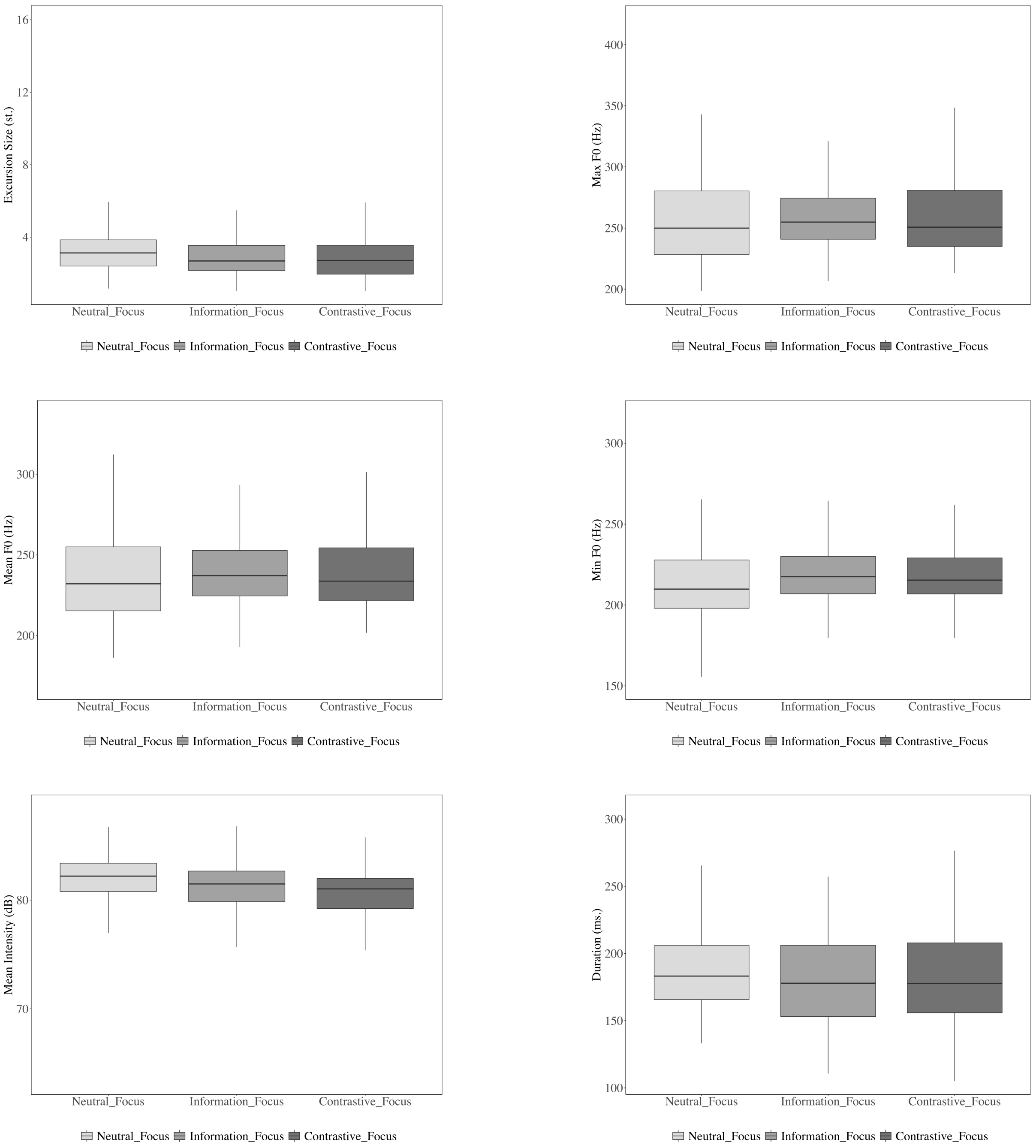

Figure 5: Penultimate focus — Pre-focus region.

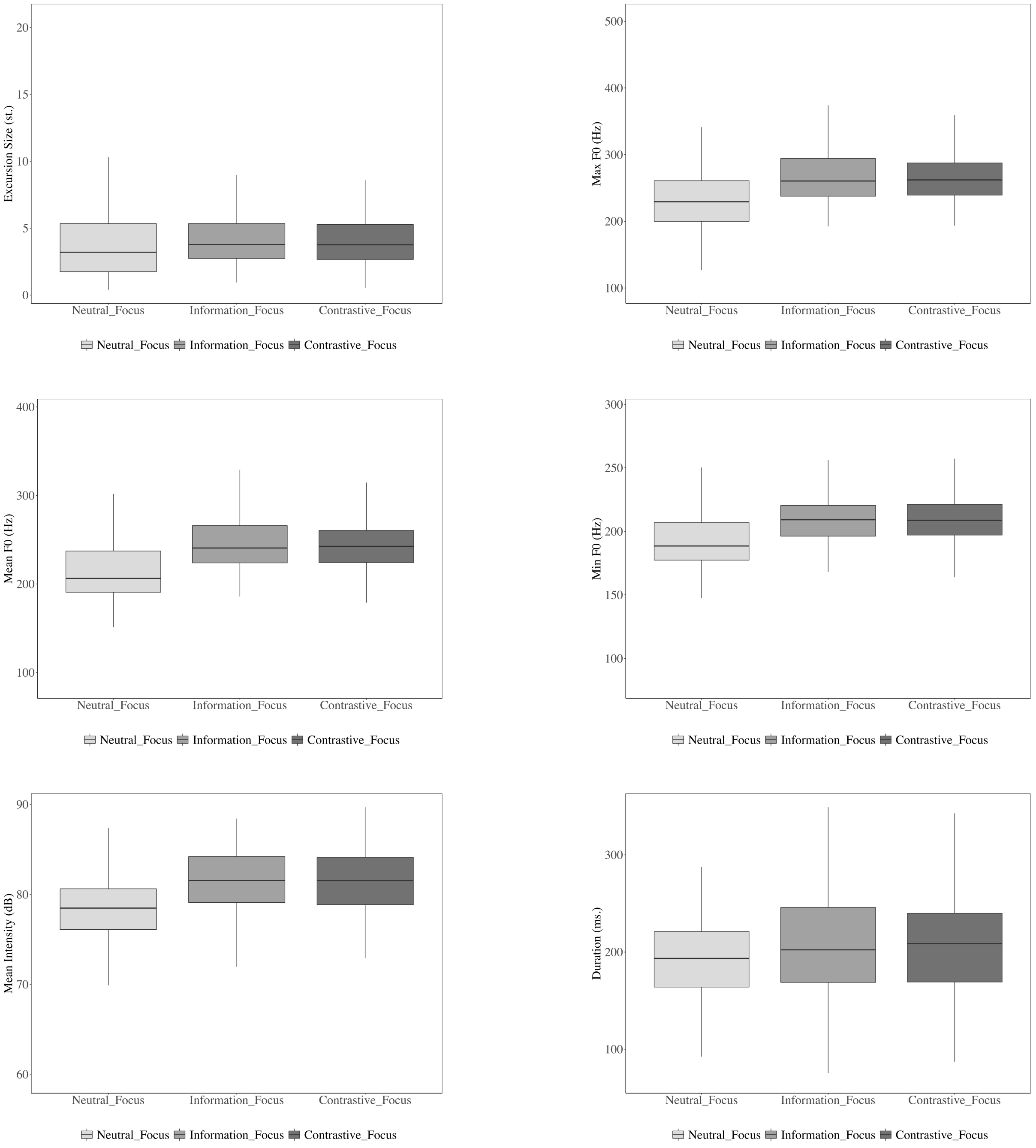

Figure 6: Final focus — On-focus region.

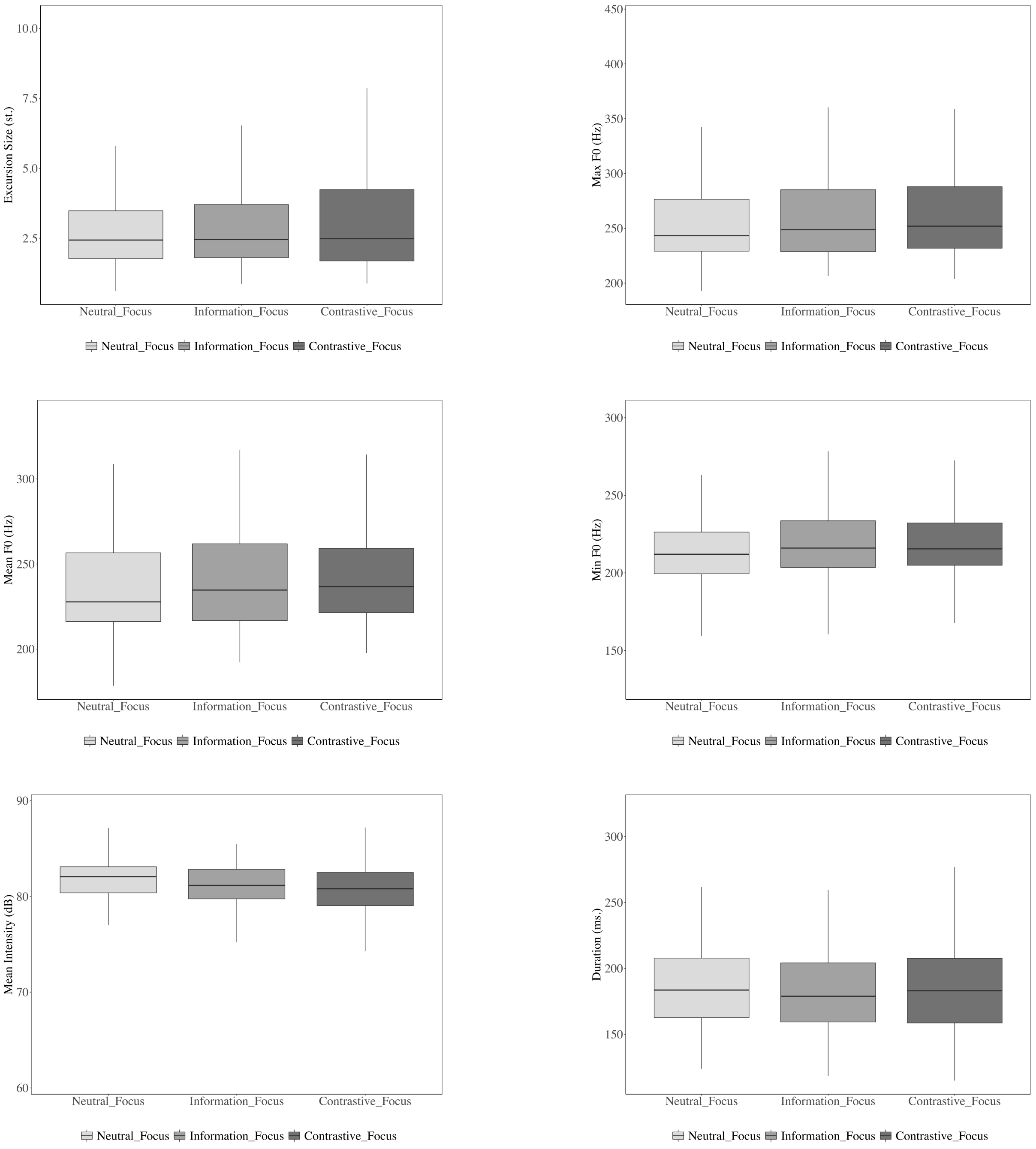

Figure 7: Final focus — Pre-focus region.
